# Emergence of Xin Demarcates a Key Innovation in Heart Evolution

**DOI:** 10.1371/journal.pone.0002857

**Published:** 2008-08-06

**Authors:** Shaun E. Grosskurth, Debashish Bhattacharya, Qinchuan Wang, Jim Jung-Ching Lin

**Affiliations:** Department of Biology, University of Iowa, Iowa City, Iowa, United States of America; Ecole Normale Supérieure de Lyon, France

## Abstract

The mouse Xin repeat-containing proteins (mXinα and mXinβ) localize to the intercalated disc in the heart. mXinα is able to bundle actin filaments and to interact with β-catenin, suggesting a role in linking the actin cytoskeleton to N-cadherin/β-catenin adhesion. *mXinα*-null mouse hearts display progressively ultrastructural alterations at the intercalated discs, and develop cardiac hypertrophy and cardiomyopathy with conduction defects. The up-regulation of mXinβ in mXinα-deficient mice suggests a partial compensation for the loss of mXinα. To elucidate the evolutionary relationship between these proteins and to identify the origin of Xin, a phylogenetic analysis was done with 40 vertebrate Xins. Our results show that the ancestral Xin originated prior to the emergence of lamprey and subsequently underwent gene duplication early in the vertebrate lineage. A subsequent teleost-specific genome duplication resulted in most teleosts encoding at least three genes. All Xins contain a highly conserved β-catenin-binding domain within the Xin repeat region. Similar to mouse Xins, chicken, frog and zebrafish Xins also co-localized with β-catenin to structures that appear to be the intercalated disc. A putative DNA-binding domain in the N-terminus of all Xins is strongly conserved, whereas the previously characterized Mena/VASP-binding domain is a derived trait found only in Xinαs from placental mammals. In the C-terminus, Xinαs and Xinβs are more divergent relative to each other but each isoform from mammals shows a high degree of within-isoform sequence identity. This suggests different but conserved functions for mammalian Xinα and Xinβ. Interestingly, the origin of Xin ca. 550 million years ago coincides with the genesis of heart chambers with complete endothelial and myocardial layers. We postulate that the emergence of the Xin paralogs and their functional differentiation may have played a key role in the evolutionary development of the heart.

## Introduction

The striated muscle-specific *Xin* gene was first identified in a differential mRNA display screen in chicken embryos and later shown to be necessary for proper cardiac morphogenesis by Xin antisense experiments [1], [2]. Since then, two genes homologous to chicken *Xin* (*cXin*) have been identified in mammals; *mXinα* and *mXinβ* (also known as *myomaxin*) in mouse [1], [3]–[5] as well as *hXinα* (also known as *cardiomyopathy associated 1*, *Cmya1*) and *hXinβ* (also known as *Cmya3*, or *Xin related protein 2*, *XIRP2*) in human [3], [6]. The word Xin was derived from the Chinese character for heart, center, or core in pronunciation. Xins localize to the adherens junctions of the intercalated discs of the heart and to the myotendinous junctions in skeletal muscle [1], [5]–[7]. Structurally, the Xin proteins are modular in nature and are defined by the presence of many copies of a 16-amino acid (aa) repeating unit (called the Xin repeat) [1], [3], [6]. The Xin repeat is known to further define an actin binding domain and a minimum of 3 Xin repeats is required to bind with actin filaments [6], 8. Recently, the full-length mXinα with 15 Xin repeats was shown to bundle actin filaments [9]. In the N-terminal sequence upstream of the Xin repeats, a Mena/VASP-binding domain in hXinα [10] and a putative DNA-binding domain in cXin and mXin [1] have been identified. The C-terminal sequence downstream of the Xin repeats contains many proline-rich regions and a filamin c-binding region was found in the last 158 aa residues of a minor and largest isoform of hXinα [1], [5], [10]. Co-localization of mXinα with both β-catenin and N-cadherin is observed throughout mouse embryogenesis and into adulthood [7]. The β-catenin binding domain is mapped to amino acid residues 535 to 636 of mXinα, which overlaps with the actin binding domains; i.e., the Xin repeats [9]. The in vitro actin binding and bundling activities of mXinα are significantly enhanced by the presence of β-catenin [9]. These results together suggest that through direct interaction, β-catenin likely recruits mXinα to the intercalated disc and activates its actin binding and bundling activities. Unlike the *cXin* antisense experiments which result in abnormal cardiac morphogenesis [1], *mXinα*-null mice are viable and fertile. However, *mXinα*-null mice exhibit numerous cardiac defects such as cardiac hypertrophy, cardiomyopathy, ultrastructural defects and conduction defects [5]. Interestingly, there is an upregulated expression of *mXinβ* at both message and protein levels in *mXinα*-null mouse hearts, suggesting mXinβ may be partially compensating for the loss of mXinα [5].

In this study, we elucidated the evolutionary history of the Xin-repeat-containing proteins. Furthermore, we identified the origin of a gene encoding Xin prior to the emergence of the Craniata. In all Craniata examined thus far, there are multiple Xin repeat-containing genes with the exception of lamprey and chicken. Data from this study revealed regions of sequence conservation and divergence among Xinαs and Xinβs. Consistent with the presence of the β-catenin-binding domain, immunofluorescence studies also revealed co-localization of Xin with β-catenin in chicken, frog and zebrafish hearts. Taken together, these results suggest that the role of Xin in linking actin with β-catenin through the conserved repeat region is one of the novel innovations that aided in the evolution of a true multi-chambered heart.

## Results

### Origin and evolution of the Xin repeats

Searching the genome databases at NCBI, e!Ensembl, Department of Energy Joint Genome Institute (DOE JGI) (http://genome.jgi-psf.org/euk_cur1.html) and Genoscope (www.cns.fr) with the conserved Xin repeat regions of mXinα, mXinβ, cXin and the zebrafish Xins, showed that the lamprey (*P. marinus*) was the earliest diverging lineage to contain a single copy of the Xin-repeat containing gene. Searches of the Cephalochordate amphioxus (*Branchiostoma floridae*) genome at DOE JGI and the Urochordate tunicate (*Ciona savignyi*) genome at NCBI with the Xin repeat regions from mXinα and lamprey Xin failed to detect a homolog. These results provisionally place the origin of Xin repeat-containing proteins within the chordate lineage after the divergence of vertebrates and Cephalochordates ca. 550 million years ago (Ma) [11].

**Figure 1:**
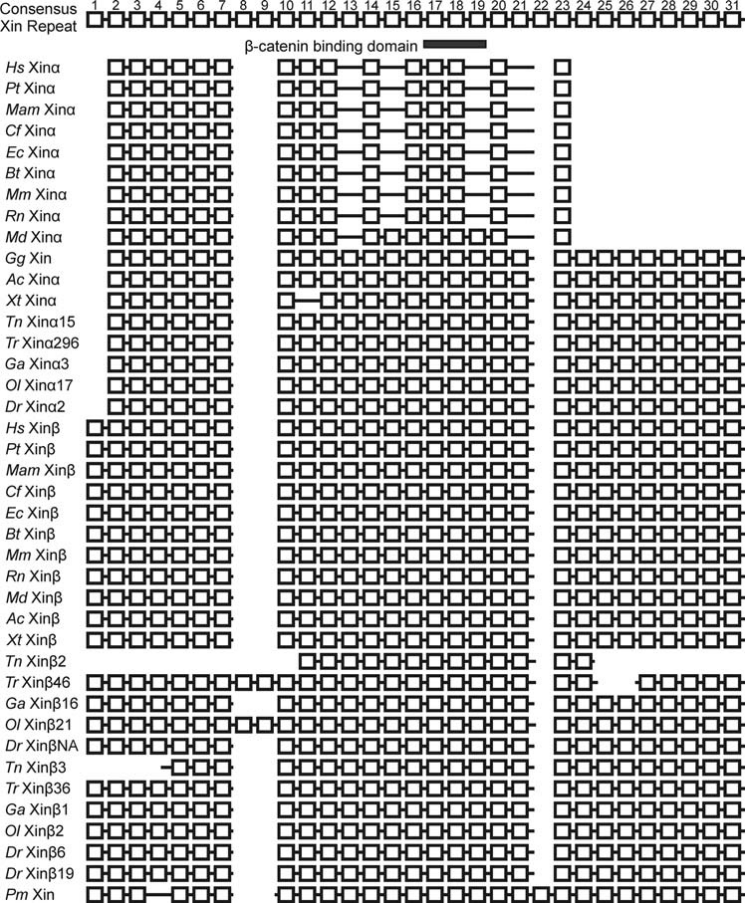
Schematic diagram of the Xin repeat alignment. The conserved Xin repeat unit is represented as a square for the 40 Xin proteins and this conserved Xin repeat region (from aa#89–742 of mXinα and aa#308–1306 of mXinβ) was used as proxies for the complete Xin sequences in this diagram [3]. The consensus Xin repreat region is comprised of 31 Xin repeat units. Large Xin repeat sequence gaps are represented as spaces in the alignment, while solid lines represent amino acids not part of a Xin repeat. The β-catenin binding domain indicated by a grey rectangle is located from the 17^th^ to the 19^th^ Xin repeat within the consensus diagram [9]. Species: *Hs*, *Homo sapien* (human); *Pt*, *Pan troglodytes* (chimpanzee); *Mam*, *Macaca mulatta* (rhesus monkey); *Cf*, *Canis lupus familiaris* (dog); *Ec*, *Equus caballus* (horse); *Bt*, *Bos taurus* (cow); *Mm*, *Mus musculus* (mouse); *Rn*, *Rattus norvegicus* (rat); *Md*, *Monodelphis domestica* (opossum); *Gg*, *Gallus gallus* (chicken); *Ac*, *Anolis carolinensis* (green anole); *Xt*, *Xenopus tropicalis* (western clawed frog); *Tn*, *Tetraodon nigroviridis* (freshwater pufferfish); *Tr*, *Takifugu rubripes* (Japanese pufferfish); *Ga*, *Gasterosteus aculeatus* (stickleback); *Ol*, *Oryzias latipes* (Japanese medaka); *Dr*, *Danio rerio* (zebrafish); *Pm*, *Petromyzon marinus* (lamprey).

A sequence alignment of the Xin repeat region from all 40 Xins demonstrated a high degree of conservation of the Xin repeat region (Figure S1). This high degree of conservation can be most readily seen in a schematic alignment of the 31 consensus Xin repeat units in the 40 Xin proteins (Fig. 1). As a conservative estimate, most Xins contain about 26∼28 repeat units within this region suggesting that this number may be important for protein function. However, a strong reduction in repeat number was observed in mammalian Xinα proteins, suggesting that this duplicated gene may have acquired a new function in mammals that is correlated with the loss of repeats in the coding region. This idea was tested by applying Tajima's relative rate test for 3 sequences using MEGA4 [12] and the Xin repeat alignment (1,201 aa). For this analysis, we used the lamprey (*P. marinus*) as the outgroup taxon and measured rate differences between the ingroup pairs of Xinα and Xinβ in human, the western clawed frog *Xenopus tropicalis*, and the opossum *Monodelphis domestica*. This analysis showed that whereas a significant rate difference was not found between the frog Xin sequences (P = 0.062), both human and opossum Xinα show a significant rate acceleration in comparison to Xinβ (P<0.000 for both cases).

**Figure 2:**
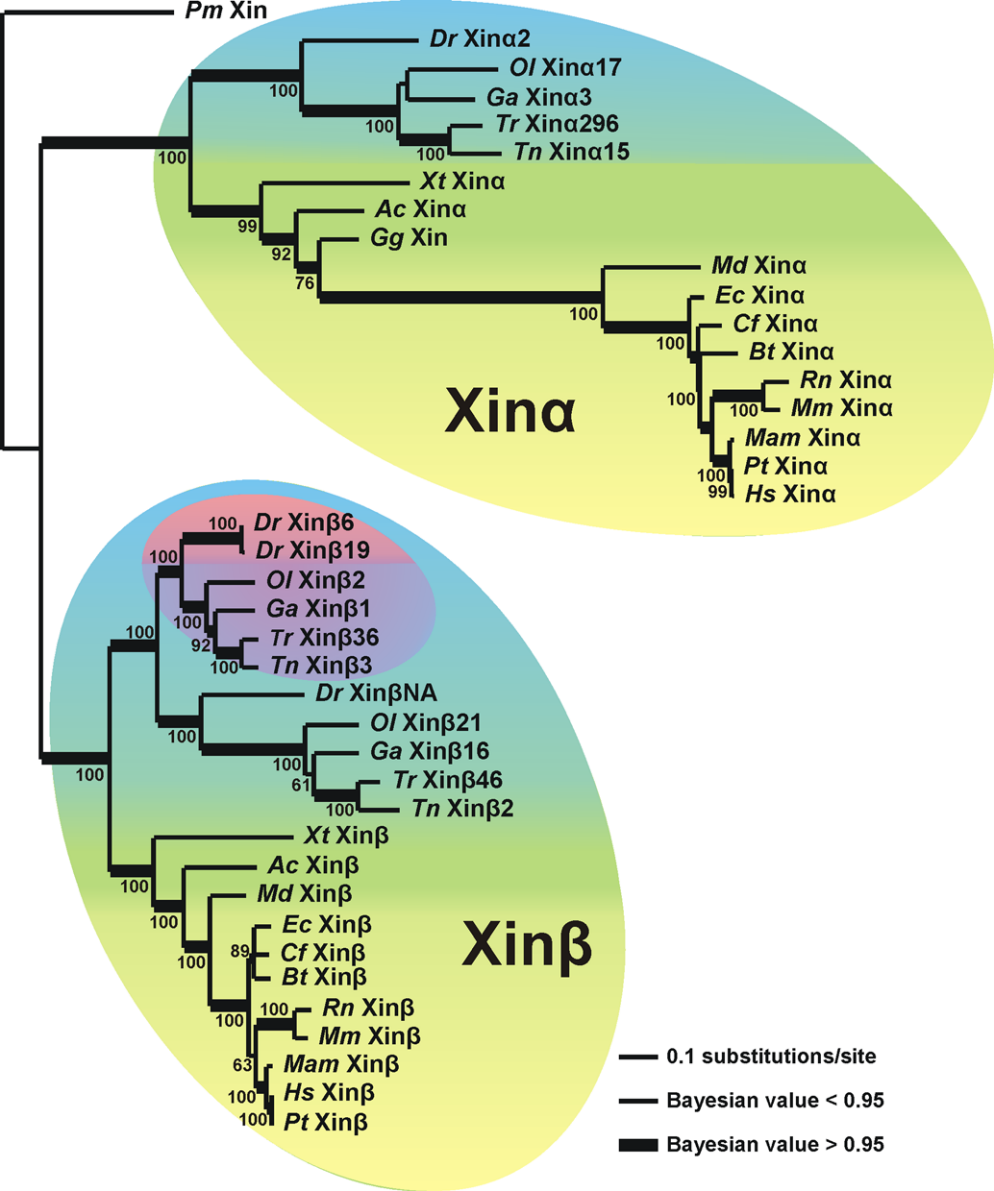
Evolutionary relationships of the Xin repeat region. The CLUSTAL W multiple sequence alignment [42] shown in Figure S1 was analyzed in maximum likelihood (ML) and Bayesian analyses. Bootstrap values are shown near each node for the ML analysis. Bayesian values greater than 0.95 are represented with thicker lines. Lamprey Xin repeat region (*Pm* Xin) was used as the outgroup. The tree replicates the phylogeny of these taxa, with the mammal, other land vertebrates and teleost (bony fish) phylum-level groups highlighted in yellow, green and blue respectively. The original gene duplication that produced Xinα and Xinβ proteins is illustrated by their monophyletic groupings that are respectively labeled. The additional multiplication of Xinβ in teleosts is highlighted in purple and the additional *Xinβ* gene duplication in zebrafish is highlighted in pink. Scale bar: 0.1 substitutions per site.

In order to clarify the phylogenetic history of the Xin repeats, the aligned sequence data was analyzed using maximum likelihood (ML) and Bayesian analyses (Fig. 2) with the lamprey Xin repeat region (*Pm* Xin) as the outgroup. The relationship of the Xin repeats within the ML tree is consistent with the broadly accepted organismal phylogeny; i.e., the teleost and mammal Xins form separate clades. A key feature of the tree is a gene duplication that produced Xinα and Xinβ. This duplication is consistent with the whole genome duplication event that occurred during early vertebrate evolution when the teleost lineage diverged from lamprey [11], [13]–[15]. In addition, the teleosts have at least three genes encoding Xin which suggests a teleost-specific genome duplication that occurred after their divergence from tetrapods [16], [17]. The subsequent loss of the presumed second Xinα in the teleost group and the loss of chicken Xinβ is explained by persistent loss of anciently duplicated genes in both the teleost and avian lineages [13], [18]. Furthermore, the presence of a zebrafish gene duplication for one of the genes encoding Xinβ (*Dr* Xinβ6 and *Dr* Xinβ19) is congruent with the finding that the zebrafish genome has undergone partial genome expansion about 140 Ma [13] resulting in the accumulation of duplicated genes after the divergence of zebrafish from *Tetraodon*.

Because no lineage prior to the lamprey apparently encodes a Xin repeat containing protein, the sequences of mXinα, mXinβ and lamprey Xin were used as queries in BLASTP searches to identify proteins that may share a common ancestry with Xin. The searches only had hits within C-terminal region of the Xins and the proteins identified with E values ranging from 3e-12 to 0.002 could be classified into one of the following groups: 1) cytoskeletal or actin interacting proteins, 2) transcription factor or nucleotide binding proteins, 3) proteins involved with G protein signaling and 4) unknown function including proline-rich protein 2 and SH3 binding protein CR16. Although these results only suggest that Xin proteins share ancestry with these proteins, the results do indicate that Xin may be derived from a protein(s) that interacted with the cytoskeleton and possibly nucleic acids.

### Conserved β-catenin binding domain within the Xin repeat region

**Figure 3:**
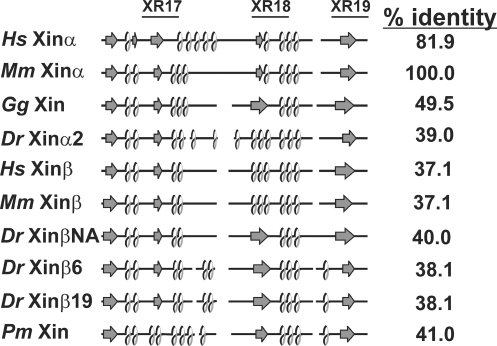
The conserved secondary structure of the β-catenin binding domain in Xin proteins. The β-catenin binding domain was previously mapped to aa#535–636 of mXinα [9] and the multiple sequence alignment of this domain from all Xins was shown in Figure S2. The secondary structure of the β-catenin binding domain of Xin proteins, based on predictions by PSIPRED [48], is schematically shown; α helices are indicated with loops, β-sheets (strands) are indicated with arrows and all other features are indicated with straight solid lines. Positions of the 17^th^∼19^th^ Xin repeats (XR) are provided with their consensus number and are indicated by a solid line.

The β-catenin-binding domain has been mapped to amino acid residues 535–636 of mXinα, which overlaps with the known actin binding domains composed of Xin repeats (XR17∼XR19) [1], [5], [7], [9]. To identify whether the β-catenin-binding domain is conserved in all Xins or alternatively is a derived trait specific to mXinα, the homologous region to the mXinα's β-catenin binding domain for the other 39 Xins were identified and aligned (Figure S2). There is significant sequence identity between various Xins within the β-catenin binding domain (from 37.1% for hXinβ or mXinβ to 81.9% for hXinα when compared to mXinα) (Fig. 3). The β-catenin binding domains of human, mouse, chicken, zebrafish, and lamprey Xin were further analyzed with PSIPRED to examine whether the secondary structure is conserved. As shown in Fig. 3, the predicted secondary structure consists of a conserved β-sheet (arrow)-α-helix (loop) repeating motif throughout this region for all Xins. Where as the armadillo repeats of β-catenin and other armadillo-containing proteins form a highly elongated structure with a positively charged groove [19], [20], the β-catenin binding domains found on various known β-catenin-interacting proteins such as N-cadherin, APC, and Tcf usually form an extended peptide with an acidic isoelectric point (pI) [21], [22]. The charge interaction appears to play an important role for the binding. The predicted pI for the β-catenin binding domain for all Xins is also acidic with a pI ranging from 4.5–5.9 for the Xinαs and 4.2–5.8 for the Xinβs, suggesting that this region is a good candidate for interactions with armadillo repeat-containing proteins.

**Figure 4:**
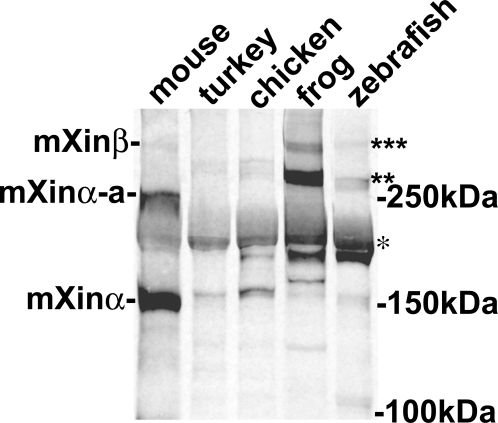
Western blot analysis of protein extracts prepared from mouse, turkey, chicken, frog and zebrafish hearts with polyclonal U1013 anti-Xin antibody. As previously reported [5], the U1013 antibody generated against the N-terminal fragment of mXinα including the Xin repeat region specifically reacts with mXinα (155 kDa), mXinα-a (250 kDa) and mXinβ (∼340 kDa) from mouse heart. Similarly, this antibody recognizes a 217 kDa band (indicated by *), and 280–295 kDa bands (indicated by**) from turkey, chicken, frog and zebrafish heart extracts. These bands may represent Xinα and its splicing variants Xinα-a. Many degraded fragments were also detected by this antibody. In frog and zebrafish but not turkey and chicken heart extracts, this antibody also reacts with a 335 kDa band (indicated by ***), which may represent Xinβ isoform.

**Figure 5:**
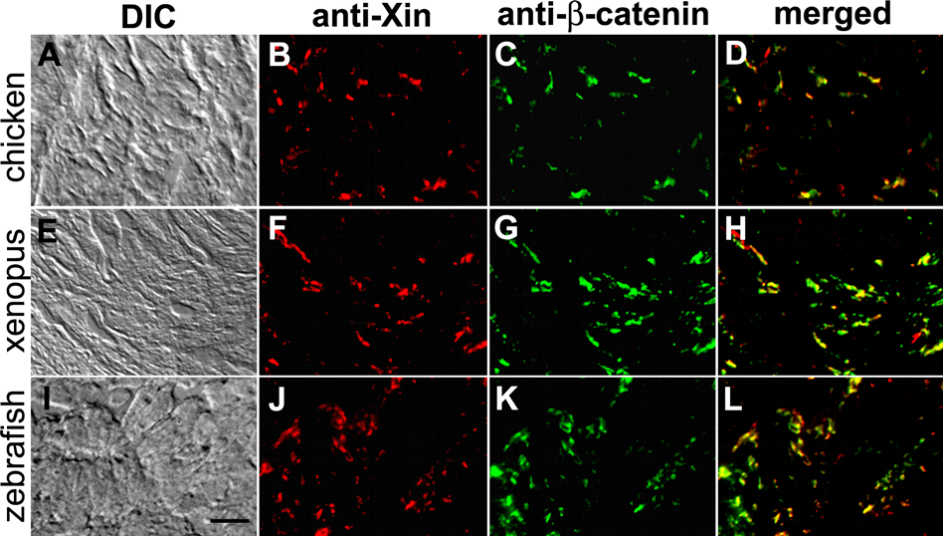
Co-localization of Xin proteins and β-catenin in chicken, frog and zebrafish hearts. Double-label indirect immunofluorescence microscopy was performed on frozen sections of chicken (A–D), frog (E–H) and zebrafish (I–L) hearts with mouse monoclonal anti-β-catenin (C, G, K) and rabbit polyclonal U1013 anti-mXin (B, F, J) antibodies, and subsequently with a mixture of rhodamine-conjugated goat anti-rabbit IgG and fluorescein-conjugated goat anti-mouse IgG. Merged images (D, H, L) indicate co-localization of the two proteins. The differential interference-contrast (DIC) images correspond to the fluorescent images shown in respectively. Scale bar: 10 µm.

**Figure 6:**
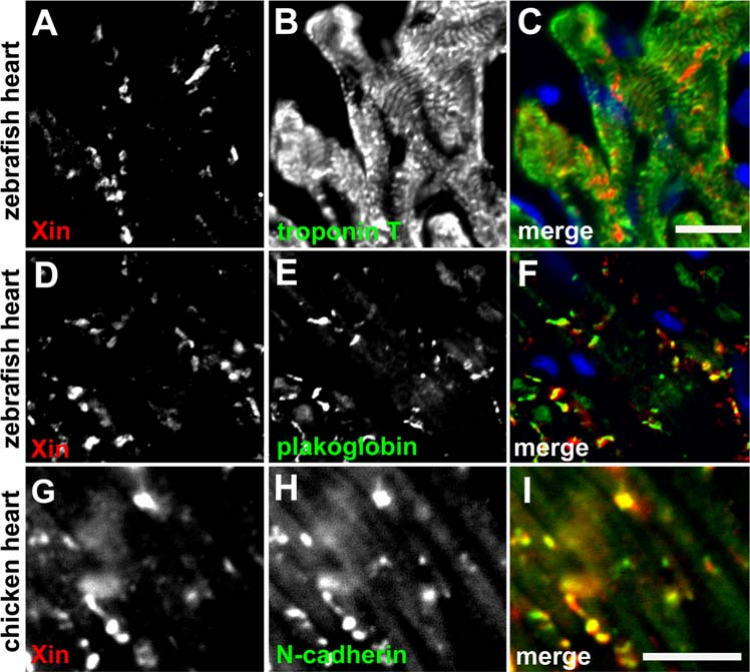
Immunofluorescence microscopy of zebrafish and chicken heart sections. (A–F) Double-label indirect immunofluorescence was performed on frozen sections of zebrafish heart with monoclonal anti-troponin T or anti-plakoglobin (green, visualized by fluorescein-conjugated 2^nd^ antibody) and polyclonal anti-mXin (red, visualized by rhodamine-conjugated 2^nd^ antibody). Before mounting, the sections were treated with DAPI to stain nuclei (blue). Scale bar: 10 µm. (G–I) Double-label indirect immunofluorescence was performed on chicken heart section with monoclonal anti-N-cadherin (green) and polyclonal anti-mXin antibody (red). Scale bar: 10 µm.

Immunofluorescence microscopy was carried out on frozen sections of chicken, frog (*Xenopus laevis*) and zebrafish (*Danio rerio*) hearts to determine whether Xin co-localization with β-catenin at the intercalated discs is evolutionarily conserved. To determine the antibody specificity, Western blot analysis was first performed on protein extracts prepared from mouse, turkey, chicken, frog, and zebrafish hearts (Fig. 4). As previously reported [5], U1013 anti-mXin antibody recognizes a major band mXinα (155 kDa) and its splicing variant mXinα-a (250 kDa) as well as a 340 kDa mXinβ band in mouse heart extract (Fig. 4). Similarly, the U1013 antibody recognizes a major band Xinα of size 217 kDa (* in Fig. 4), its splicing variant of size 282∼295 kDa (** in Fig. 4) and some degraded fragments (160∼198 kDa) from turkey, chicken, frog and zebrafish heart extracts. In addition, the antibody cross-reacts with a minor band of size 335 kDa that appears to be Xinβ from frog and zebrafish heart extracts but not from turkey and chicken heart extracts (*** in Fig. 4). As previously demonstrated in the mouse heart [7], double-label immunofluorescence microscopy also revealed a high degree of colocalization of Xin with β-catenin to the structure that appears to be intercalated discs in chicken, frog, and zebrafish hearts (Fig. 5). Unlike that seen in the mouse heart section, the fluorescence intensity of Xin at each location in chicken, frog and zebrafish heart sections was not totally equal to that of β-catenin. Thus, only portion of colocalizations in merged images shows yellow color (Fig. 5D, H and L); however, the overall staining patterns for Xin and β-catenin are very similar. Specificity for the anti-Xin antibody to structures of the intercalated disc was demonstrated by the lack of colocalization of Xin with a thin filament protein, cardiac troponinT, in the chicken (data not shown), frog (data not shown) and zebrafish (Fig. 6B and C) hearts at higher magnification. A high degree of colocalization of Xin with N-cadherin, a known β-catenin-binding protein, to the intercalated disc was also detected in frog (data not shown) and chicken heart sections (Fig. 6G–I). Certain colocalization of Xin with plakoglobin, other intercalated disc marker found in both adherens junctions and desmosomes, to the intercalated disc could be observed in zebrafish heart section (Fig. 6D–F). Unfortunately, we could not determine the colcalization of Xin with gap junction components, since the anti-connexin43 antibody that we used did not cross react with connexin 43 from chicken, frog and zebrafish hearts. This co-localization of Xin with β-catenin across evolutionarily distantly related lineages further supports the existence of a highly conserved β-catenin-binding domain.

### Evolution of other binding domains in Xin proteins

**Figure 7:**
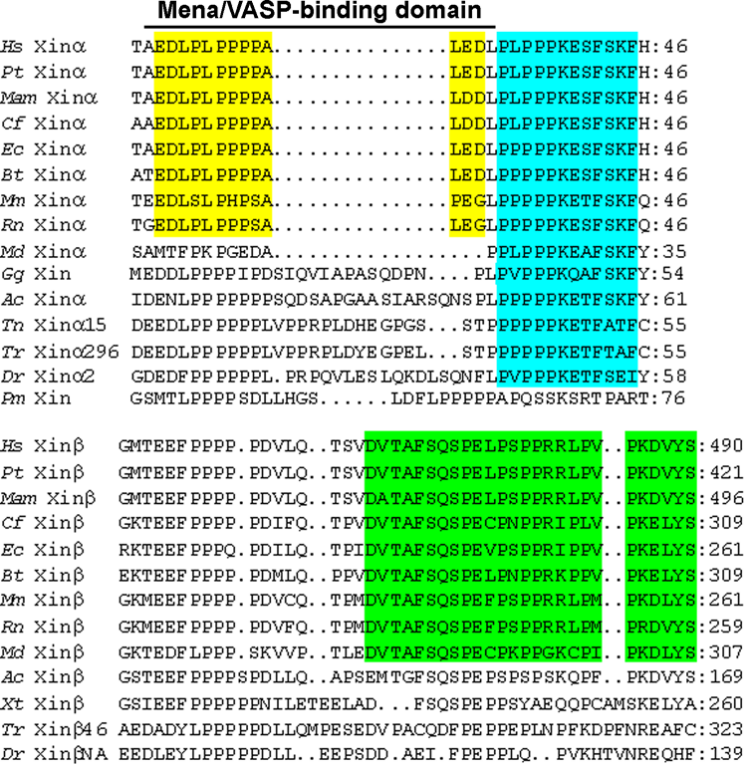
Multiple sequence alignment of the Mena/VASP-binding domain of the Xin proteins. The previously identified Mena/VASP-binding domain and its immediately downstream sequence including putative DNA binding domain from aa#18–69 of hXinα was used to align all Xin proteins (Figure S3). Only aa#18–46 sequence of hXinα and its homologous sequences from some of Xin proteins are shown. The Mena/VASP binding domain (AEDLPLPPPPALEDL) only conserves in the placental mammal Xinα proteins. Interestingly, the sequences downstream of the Mena/VASP-binding domain are highly conserved among all mammalian Xin proteins (indicated by light blue and green boxes) including opossum Xin, however, these sequences are very divergent between Xinα and Xinβ, representing unidentified functional domains unique to Xin isoforms.

Other than the Xin repeat region and the β-catenin binding domain, there is a previously characterized Mena/VASP-binding domain [10] and a putative DNA binding domain [1] in the N-terminal region upstream of the Xin repeats. The N-terminal regions were aligned and then examined for the homologous sequences for these domains. Most (36/40) of the Xins share significant identity in the putative DNA binding domain and its immediate upstream region (Figure S3). The Mena/VASP-binding domain (EDLPLPPPPALED) in hXinα was previously shown to directly interact with the EVH1 domain of Mena and VASP family proteins, actin cytoskeleton modulators [10]. Interestingly, the deletion of two acidic residues from either the N- or C-terminus of this domain greatly reduced or completely abolished the binding ability, respectively. Based on these criteria and sequence alignment, only Xinα proteins from the placental mammals but not from opossum (*Md* Xinα) contain this consensus Mena/VASP-binding domain (yellow box in Fig. 7). In the Xinβ proteins of the placental mammals, there is a proline-rich region similar to that in the Mena/VASP-binding domain; however, absence of the C-terminal two acidic residues may suggest a lack of function. Therefore, the Mena/VASP-binding domain appears to be a novel feature of Xinα proteins in placental mammals. It should be noted that the sequences downstream of this Mena/VASP-binding domain of Xinα are also highly similar (light blue and green boxes in Fig. 7), although there is currently no assigned function for this region.

The variable C-terminal regions immediately downstream of the Xin repeats were aligned and examined for the conserved regions (Figure S4), the proline-rich regions [1] (Figure S5) and the previously characterized filamin c-binding region [10] (Figure S6). Overall, these alignments revealed that Xinαs and Xinβs are highly diverged at their C-termini, suggesting that each gene may play a unique function in the striated muscle.

### The origin of Xin coincides with the genesis of heart chamber

**Figure 8:**
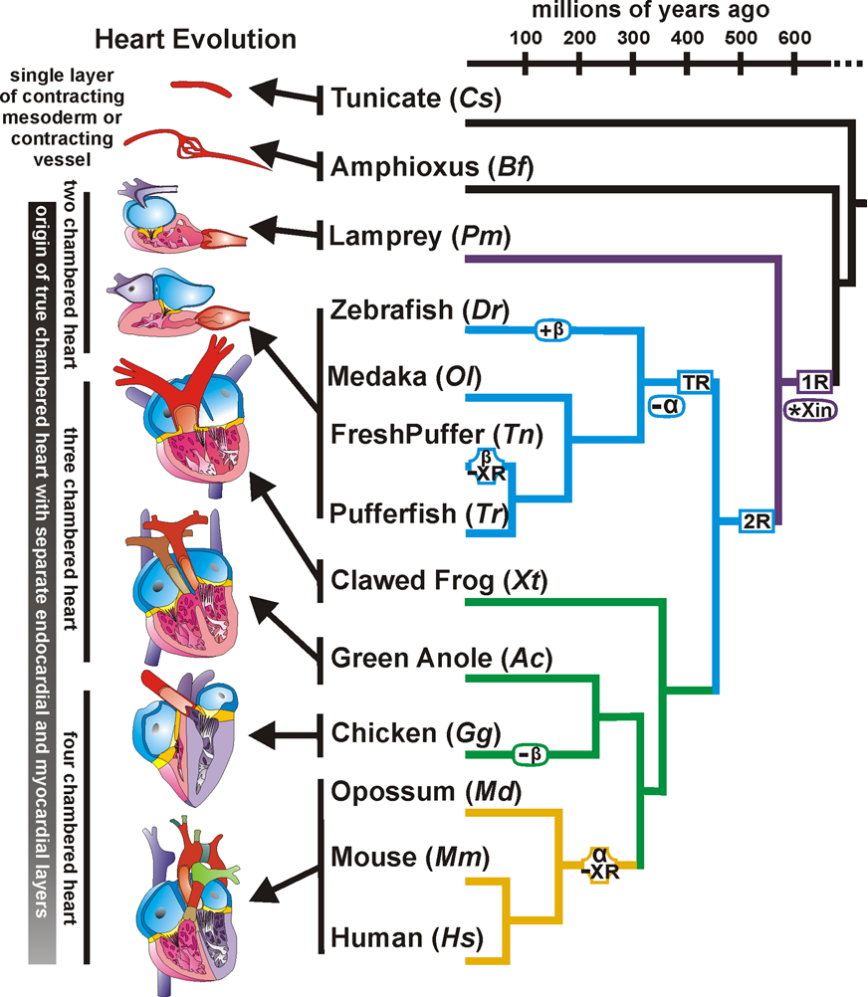
Phylogenetic tree of vertebrates showing the evolutionary relationships of the Xin proteins and heart. A phylogeny representing the evolutionary time-scale of Urochordata to Craniata with the lamprey, teleost, other land vertebrates and mammal branches colored in purple, blue, green and beige respectively. This phylogeny also illustrates (i) the two rounds of vertebrate genome duplications (1R and 2R boxed in purple and blue respectively) as well as the teleost-specific genome duplication (TR also boxed in blue), (ii) the emergence of Xin after the first round of vertebrate genome duplications (*Xin in purple oval), (iii) loss of the duplicate Xinα in the teleost lineage (−α in blue oval), (iv) single Xinβ duplication in zebrafish (+β in blue oval), (v) loss of Xin repeats in the freshwater pufferfish Xinβ (β -XR in blue octagon), (vi) loss of Xinβ in chicken (−β in green oval) and (vii) loss of Xin repeats in the mammalian Xinα (α -XR in beige octagon). No Xin proteins were identified in the Urochordate tunicate (*Cs*, *Ciona savignyi*) or the Cephalochordate amphioxus (*Bf*, *Branchiostoma floridae*), which contains a single layer of contracting mesoderm or contracting vessel with incomplete endothelial cell layer. A Xin protein is first identified in the Craniate lamprey (*Pm*, *Petromyzon marinus*) which contains true chambered heart with endothelial and myocardial layers.

A phylogenetic tree was adapted from previous work [23], [24] to illustrate the evolutionary relationships of various vertebrates and their Xin proteins over a broad evolutionary time-scale (Fig. 8). Using this phylogenetic framework, the origin of the Xin repeat-containing proteins is placed roughly 550 Ma prior to the emergence of lamprey and between the two rounds (1R and 2R in Fig. 8) of vertebrate genome duplication [11], [15]. This assignment of the origin of Xin is consistent with the apparent absence of Xin in either the Urochordate tunicate (*Cs*, *Ciona savignyi*) or the Cephalochordate amphioxus (*Bf*, *Branchiostoma floridae*). BLAST searches using genome data from *Saccharomyces cerevisiae* (baker's yeast), *Candida albicans* (fungal yeast pathogen), *Arabidopsis thaliana* (mouse-ear cress plant), *Dictyostelium discoideum* (amoeba), *Caenorhabditis elegans* (worm), *Anopheles gambiae* (mosquito) and *Drosophila melanogaster* (fruit fly) also failed to detect Xin in these more distantly related taxa. Our hypothesis for the origin of Xin is also supported by the finding of a single gene encoding Xin in lamprey. Uniquely, *Pm* Xin lacks XR4 but has XR22, which likely represents the original Xin repeat organization and number (Fig. 1). Thereafter, all Xins except *Tn* Xinβ2 and *Tn* Xinβ3 gained XR4 and deleted XR22.

Given this interesting and clear pattern of gene and genome duplication in vertebrates it is tempting to speculate how Xin proteins may have played a role in the evolution of the heart. The tunicate and amphioxus models provide an important comparative tool for understanding pre-Xin heart evolution. In the tunicate, the heart is a tubular structure that does not coordinate directional blood flow and the heart tube is composed of a single layer of myoepithelium surrounded by a single layer of pericardial coelom [25], [26]. In amphioxus, the heart consists of several contractile vessels composed of a myoepithelial layer which are not continuously lined with an endothelium [27], [28]. However, after the origin of Xin many novel features are observed in the heart. In the lamprey, these include a S-shaped heart consisting of four regions: a sinus venosus, a contracting atrium, a contracting ventricle and conus arteriosus [29]; the presence of valves to promote unidirectional blood flow; the origin of true heart chambers (atrium and ventricle) with both endothelial and myocardial layers [30] (Fig. 8).

The heart in zebrafish and lamprey are similar in organization and consist of two chambers with complete endothelial and myocardial layers which are also hallmark features of the heart in all later-diverging taxa [30], [31]. The presence of one Xinα and multiple copies of Xinβ in teleost is rather perplexing because one would assume that one *Xin* gene which is sufficient in lamprey would also be sufficient in the teleost lineage. However, vertebrates have evolved a nerve system to modulate cardiac activity in a sophisticated fashion [32]. These activities include heart rates, contractile force, action potential and conduction velocity. In this regard, it is known that both vagus nerve and sympathetic nerve fibers innervate the mammalian and teleost hearts, whereas only the vagus nerve functions in the adult lamprey heart [33], [34]. Therefore, multiple *Xin* genes may be required for responding to different cardiac outputs in different animals and/or in different regions of the heart.

In tetrapods, two unique events occurred: 1) in the avian lineage there is a loss of Xinβ, and 2) in the mammalian lineage, Xinα contains a reduced number of Xin repeat units with 17 repeats in marsupials and 15 in placental mammals. Whereas there are dramatic morphological changes in the organization of the heart from a two chambered heart with an atrium and a ventricle in lamprey, to a three chambered heart with two atria and a ventricle in the amphibian and reptile, and finally to a four chambered heart with two atria and two ventricles in the avian and mammal (Fig. 8); the heart chambers still consist of an endothelial layer and a myocardial layer [35]. The morphological changes in the heart cannot be a plausible explanation for the loss of Xinβ in the avian lineage or the loss of repeats in the mammal Xinα. It is likely that physiological and functional changes in the heart are responsible for these evolutionary adaptations.

## Discussion

Xin originated prior to the emergence of lamprey roughly 550 Ma after which the vertebrate lineage experienced a whole genome duplication [11], [13]–[15] resulting in the appearance of Xinα and Xinβ. Prior to the teleost lineage, a teleost-specific whole genome duplication [16], [17] resulted in the duplication of the genes encoding Xinα and Xinβ. However, all of the teleost species examined have lost one of the genes encoding Xinα, suggesting that Xinα loss occurred prior to the teleost radiation. Interestingly, zebrafish has 3 genes encoding Xinβ indicating that a recent single gene duplication of one of their *Xinβ* genes derived the genes encoding Xinβ6 and Xinβ19. Most of the tetrapods which include the amphibians, reptiles, and mammals have two *Xin* genes. However, the avian species (chicken and probably turkey) do not have a gene encoding Xinβ. BLAST searches of the zebra finch (*Taeniopygia guttata*) genome at NCBI with the chicken Xin sequence did not return any significant hits, which may indicate incomplete annotation of this genome. Thus, whether the *Xinβ* gene was lost in only chicken or a larger subset of avian species remains unclear.

The ancestral lamprey Xin repeat region contains 28 Xin repeats that are highly conserved in most other vertebrate Xins (Fig. 1). However, there are two instances in which Xin proteins deviate from this number: 1) Xinβ in the freshwater pufferfish and 2) Xinα in the mammal lineage. The freshwater pufferfish Xinβ2 and Xinβ3 contain 13 and 24 Xin repeat units, respectively. A possible explanation for the fewer repeats could be a consequence of the reduced genome size within this species since the freshwater pufferfish genome has been completely annotated [16] and no other teleost Xin repeat regions contain fewer than the consensus number of repeats. The marsupial and all placental mammal Xinα proteins contain 17 and 15 Xin repeats, respectively. This strongly suggests that there was selective pressure within the mammalian lineage that resulted in the reduction of Xin repeats. The number of Xin repeats appears to be important for the function of the Xin proteins for a variety of reasons. Three Xin repeats are sufficient for binding actin [6], [8] and six repeats are sufficient for binding and cross-linking actin filaments [6], [8]. Recently, the full-length mouse Xinα with fifteen Xin repeats has been shown to bundle actin filaments [9].

Both the β-catenin-binding domain and putative DNA binding domain appear to be ancestral domains found in all Xinαs and Xinβs. The secondary structure of the β-catenin binding domain is evolutionarily conserved. More importantly, there is strong co-localization of Xin with known intercalated disc components, such as β-catenin, N-cadherin and plakoglobin in the mouse, chicken, frog and fish hearts. This suggests that Xin localization to the intercalated disc in the heart is evolutionarily conserved and may also indicate that the direct interaction between the two proteins is also conserved [9]. Unfortunately, the function of the highly conserved, putative DNA binding domain is unknown. In contrast, the Mena/VASP-binding domain is a derived trait found only in placental mammal Xinαs. In the C-terminus downstream of the Xin repeats, there exist many conserved regions among either Xinαs or Xinβs but divergent between Xinα and Xinβ even within the same species. For example, the proline-rich regions are strongly conserved in most Xins but do not appear to be homologous regions when comparing the Xinα proteins to the Xinβ proteins or when comparing the fish Xins to the tetrapod Xins. Although these analyses did not directly identify where or how the Xin repeat region originated, they did provide us with a clearer picture of how the regions within these proteins evolved.

Because the early diverging lamprey contains a single copy *Xin* gene and almost all other vertebrates have more than one, there should be a mechanism for the maintenance of these duplicated genes. Duplicated genes are believed to have four possible fates. The first is nonfunctionalization, whereby one gene is lost to mutation (i.e., becomes a pseudogene). The second is neofunctionalization, whereby one duplicated gene retains the ancestral function and the second gains a novel related function. The third is subfunctionalization or ‘duplication-degeneration-complementation’ whereby the original ancestral functions are partitioned between the duplicate genes. And finally duplicate genes may be maintained as multiple copies of nearly identical sequence due to positive selection for the dosage of the gene products [36]–[39]. It appears that duplicate Xin proteins may be retained for dosage or potentially neofunctionalization in the mammalian lineage. The significant protein divergence rate acceleration that is evident in mammalian Xinα is consistent with the latter view. Both mXinα and mXinβ however localize to the adherens junctions at the intercalated discs in the heart [5], [7]. The localization at the intercalated disc is presumably through the interaction between the conserved β-catenin binding domain and β-catenin [9]. This suggests that both Xinα and Xinβ have a similar (i.e., but not necessary identical) role at the intercalated discs. Interestingly, loss of *Xinα* in mice results in the up-regulation of the homologous *Xinβ* at both message and protein levels [5], suggesting that a particular dosage of Xin is required for proper heart development and function. This also suggests that the two proteins can partially compensate for each other because the disruption of *Xin* expression in chicken results in abnormal cardiac morphogenesis, whereas the *Xinα*-null mouse heart still develops normally. However, defects are observed in adult *Xinα*-null mouse heart [5] which demonstrates that Xinβ function cannot fully compensate for Xinα in the adult heart. Presumably, the novel and likely derived functional roles of mammalian Xinα reside in the derived features such as the Mena/VASP-binding domain, the proline-rich regions, and other unidentified regions that are exemplified by the rate acceleration evident in the Xinα repeat region. Functional studies of these derived features will provide the strongest evidence for neofunctionalization in Xinα.

Regardless of the underlying mechanism by which the duplicated *Xin* genes and the proteins they encoded have been maintained, all of the Xin proteins contain the highly conserved Xin repeat region and appear to localize to structures resembling the myocardial cell-cell contacts referred to as intercalated discs. The origin of the Xin proteins that coincides with the origin of a true cardiac chamber with separate endothelial and myocardial layers introduces the possibility that Xin is one of many proteins co-opted for the development and maintenance of the myocardium. Because disruption of the chicken *Xin* gene results in abnormal cardiac morphogenesis [1] and *mXinα*-null mice exhibit cardiac hypertrophy, cardiomyopathy, ultrastructural defects, as well as conduction defects [5], the Xin proteins are clearly key players in heart development and maintenance. Defects in a large set of sarcomeric proteins such as cardiac β myosin heavy chain, α-tropomyosin, titin, and cardiac α-actin have been demonstrated to induce cardiac hypertrophy [40], [41]. This evidence suggests that diseases like cardiac hypertrophy may indeed arise from mutations from independent genes whose proteins have coevolved for the development and/or maintenance of specific cellular or tissue structures.

## Materials and Methods

### Search for Xin repeat-containing DNA sequences and Xin ancestral proteins

Sequences for human (*Hs*, *Homo sapien*), chimpanzee (*Pt*, *Pan troglodytes*), rhesus monkey (*Mam*, *Macaca mulatta*), dog (*Cf*, *Canis familiaris*), horse (*Ec*, *Equus caballus*), cow (*Bt*, *Bos Taurus*), mouse (*Mm*, *Mus musculus*), rat (*Rn*, *Rattus norvegicus*), opossum (*Md*, *Monodelphis domestica*), chicken (*Gg*, *Gallus gallus*), green anole (*Ac*, *Anolis carolinensis*) and zebrafish (*Dr*, *Danio rerio*) were downloaded from NCBI (http://www.ncbi.nlm.nih.gov) using the Xin repeat region from mXinα and cXin as query sequences in protein BLAST searches. The sequences for western clawed frog (*Xt*, *Xenopus tropicalis*), Japanese pufferfish (*Tr*, *Takifugu rubripes*), stickleback (*Ga*, *Gasterosteus aculeatus*), Japanese medaka (*Ol*, *Oerzias latipes*) and lamprey (*Pm*, *Petromyzon marinus*) were downloaded from the e!Ensembl or Pre!Ensembl databases (http://www.ensembl.org/index.html) using the Xin repeat regions from cXin or the zebrafish Xins as query sequences in protein BLAST searches. The freshwater pufferfish (*Tn*, *Tetraodon nigroviridis*) sequences were downloaded from the Genoscope Tetraodon Genome Browser (http://www.genoscope.cns.fr/externe/tetranew/) using the Xin repeat regions from the zebrafish Xins as query sequences in protein BLAST searches. The green anole (*Ac*) Xinα and Xinβ, western clawed frog (*Xt*) Xinα, freshwater pufferfish (*Tn*) Xinβ2 and lamprey (*Pm*) Xin sequences were predicted from genomic contig sequences using GENSCAN (http://genes.mit.edu/GENSCAN.html) with the default parameters. In general, the larger Xin protein isoform sequences were used for this study, such as the characterized large hXinα-A in human [10] and large mXinα-a in mouse [5], [9], and then the sequences were examined for consistency since many of the protein sequences are predictions from genomic DNA. For this reason, the green anole Xinα and freshwater pufferfish Xinα15 sequences were shortened at the C-terminus to be more consistent with the terrestrial Xinα and fish Xinα, respectively. Most of the sequences obtained were of exceptional quality, since they were obtained from completed or nearly completed genome builds. For the sequences that were from incompleted genome builds, the obtained contig sequence regions used were sufficiently long reads that had no unresolved nucleotides. Table 1 contains a summary of the identification information, source and editing for all the sequences used in this study.

**Table 1:** The DNA sequences used for analysis are listed with their respective species, GenBank accession number or other identifier, Xin isoform as well as a brief description for the nature of the sequence.

Species	Accession number	Xin isoform	Sequence description
*Homo sapiens* (*Hs*)	AJ626900	Xinα	cDNA
*Homo sapiens*	NM_152381	Xinβ	cDNA
*Pan troglodytes* (*Pt*)	XR_025190	Xinα	Genomic DNA
*Pan troglodytes*	XR_025597	Xinβ	Genomic DNA
*Macaca mulatta* (*Mam*)	XM_001084767	Xinα	Genomic DNA
*Macaca mulatta*	XR_011865	Xinβ	Genomic DNA
*Canis familiaris* (*Cf*)	XM_846492	Xinα	Genomic DNA
*Canis familiaris*	XM_535943	Xinβ	Genomic DNA
*Equus caballus* (*Ec*)	XM_001501989	Xinα	Genomic DNA
*Equus caballus*	XM_001497011	Xinβ	Genomic DNA
*Bos taurus* (*Bt*)	XM_871103	Xinα	Genomic DNA
*Bos taurus*	XR_027420	Xinβ	Genomic DNA
*Mus musculus* (*Mm*)	NM_00181339	Xinα	cDNA
*Mus musculus*	AY775570-775571 and EU286528-286531	Xinβ	cDNA
*Rattus norvegicus* (*Rn*)	XM_236702	Xinα	Genomic DNA
*Rattus norvegicus*	NM_201989	Xinβ	cDNA
*Monodelphis domestica* (*Md*)	XM_001362457	Xinα	Genomic DNA
*Monodelphis domestica*	AAFR03008876	Xinβ	Genomic DNA* cont3.008875
*Gallus gallus* (*Gg*)	AF051944	Xin	cDNA
*Anolis carolinensis (Ac)*	AAWZ01008626	Xinα	Genomic DNA* + cont1.8625
*Anolis carolinensis*	AAWZ01028693	Xinβ	Genomic DNA* cont1.28692
*Xenopus tropicalis* (*Xt*)	Scaffold 28, 50–150 Kb	Xinα	Genomic DNA*
*Xenopus tropicalis*	ENSXETT00000036439	Xinβ	Genomic DNA
*Tetraodon nigroviridis* (*Tn*)	GSTENT00015114001	Xinα15	Genomic DNA+
*Tetraodon nigroviridis*	Chromosome 2	Xinβ2	Genomic DNA*
*Tetraodon nigroviridis*	GSTENT00024833001	Xinβ3	Genomic DNA
*Takifugu rubripes* (*Tr*)	GENSCAN00000003601	Xinα296	Genomic DNA
*Takifugu rubripes*	GENSCAN00000018589	Xinβ36	Genomic DNA
*Takifugu rubripes*	GENSCAN00000017305	Xinβ46	Genomic DNA
*Gasterosteus aculeatus* (*Ga*)	ENSGACP00000016735	Xinα3	Genomic DNA
*Gasterosteus aculeatus*	ENSGACP00000018393	Xinβ1	Genomic DNA
*Gasterosteus aculeatus*	ENSGACP00000007099	Xinβ16	Genomic DNA
*Oryzias latipes* (*Ol*)	ENSORLP00000014709	Xinα17	Genomic DNA
*Oryzias latipes*	ENSORLP00000004267	Xinβ2	Genomic DNA
*Oryzias latipes*	chromosome 21 scaffold	Xinβ21	Genomic DNA*
*Danio rerio* (*Dr*)	NM_001012377	Xinα2	cDNA
*Danio rerio*	XM_001343751	Xinβ6	Genomic DNA
*Danio rerio*	XM_001332521	Xinβ19	Genomic DNA
*Danio rerio*	XM_001337295	XinβNA	Genomic DNA
*Petromyzon marinus* (*Pm*)	contig 20990, 1–14,558 bp	Xin20990	Genomic DNA*

* indicates sequence that was annotated manually from the genomic DNA sequence with Genscan.

+ indicates protein sequence was manually annotated to specify consistent translational start site with other known protein sequences.

The full-length and Xin repeat region amino acid sequences of mXinα, mXinβ as well as lamprey Xin were used as BLAST queries at NCBI to identify proteins with which Xin may share a common ancestry. BLASTP was used to search the mouse build protein database with the full-length Xin sequences, using no filter and an expect value = 1. Because no hits were made in the Xin repeat region, BLASTP was also used to search the mouse RefSeq protein database with the Xin repeat region sequences, using no filter and expect value = 10. Those hits with gene names were then investigated for their annotated function through database searching at the PANTHER (Protein ANalysis THrough Evolutionary Relationships, http://www.pantherdb.org) classification system.

### Sequence alignment and phylogenetic reconstruction

The various Xin protein sequences were assembled in FASTA format in YooEdit V1.71 (http://www2s.biglobe.ne.jp/yex/YooEdit/). Multiple sequence alignments were performed using ClustalWPPC: CLUSTAL W version 1.7 [42] software with default parameters as well as using servers at EBI Tools (http://www.ebi.ac.uk/Tools/clustalw/index.html) or at Pôle Bioinformatique Lyonnais (http://npsa-pbil.ibcp.fr). The final alignments were manually inspected and edited using the sequence alignment editor SE-Al version 1.d1 software [43]. In particular, we focused our attention on the Xin repeat alignment. Prior to the phylogenetic analysis of these data, we used ProtTest V1.3 [44] to identify the best-fit model using the Akaike Information Criterion (AIC) criterion (i.e., JTT+Γ+I). This model was then used in a RAxML (RAxML-VI-HPC, v2.2.1) [45] maximum likelihood analysis. This analysis used a random starting tree (one round of taxon addition) and the rapid hill-climbing algorithm (i.e., option -f d in RAxML). To generate bootstrap values, we did a RAxML bootstrap analysis (100 replicates) as described above and under PHYML (V2.4.3, [46] 200 replicates) with tree optimization using the JTT+Γ+I model. We used Bayesian inference (MrBayes V3.0b4, [47] with the Xin repeat alignment using the JTT+Γ+I model to calculate posterior probabilities for nodes in the RAxML tree. Metropolis-coupled Markov chain Monte Carlo from a random starting tree was used in this analysis with two independent runs (i.e., nrun = 2 command) and 1 cold and 3 heated chains. The Bayesian analyses were run for 600,000 generations with trees sampled every 100th generation. To increase the probability of chain convergence, we sampled trees after the standard deviation values of the two runs were <0.01 to calculate the posterior probabilities (i.e., after 94,200 generations). The remaining phylogenies were discarded as burn-in. In all of these phylogenetic analyses, the branch leading to the lamprey (*Petromyzon marinus*, *Pm* Xin) was used to root the tree. Sequences were also aligned using Clustal W with the MegAlign program within the Lasergene 6 software suite (DNASTAR, Inc., Madison, WI) and used to calculate the percent identity between two sequences. The secondary structure of the β-catenin-binding domains on Xins were analyzed with the software program PSIPRED (http://bioinf.cs.ucl.ac.uk/psipred) [48]. The isoelectric points of the β-catenin binding domain and Xin repeat region for all the Xins was determined from the primary amino acid sequence using the protein characteristics feature of the Editseq program within the Lasergene 6 software suite.

### Immunofluorescence and Western blot analysis

Frozen sections of chicken, frog (*Xenopus laevis*) and zebrafish hearts were used in immunofluorescence microscopy as previously described [7]. The primary antibodies used included the rabbit polyclonal U1013 anti-mXin antibody [7] and mouse monoclonal anti-β-catenin antibody (Invitrogen-Zymed, Carlsbad, CA), anti-N-cadherin (Invitrogen-Zymed), anti-plakoglobin (BD Biosciences Pharmingen, San Diego, CA) and anti-troponin T (CT3) [1] antibody. Frozen sections from two adult hearts for each organism were immunostained with a mixture of anti-mXin and anti-β-catenin primary antibodies, and subsequently reacted with a mixture of rhodamine-conjugated anti-rabbit IgG and fluorescein-conjugated anti-mouse IgG secondary antibodies. The fluorescent staining was visualized using a Leica DMIRE2 microscope and imaged with a cooled CCD camera (QImaging, Burnaby, BC, Canada) and Openlab software. After immnuostaining, some sections were treated with 0.3% 4′,6′-diamidino-2-phenylindole (DAPI; Sigma, St Louis, MO) for 15 min, washed and mounted for microscopy.

Western blot analysis of protein extracts prepared from mouse, turkey, chicken, frog (*Xenopus laevis*) and zebrafish hearts was carried out with rabbit polyclonal U1013 anti-mXin antibody as previously described [5], except that the Odyssey Infrared Imaging System (LI-COR, Inc., Lincoln, NE) with Alexa 680-labeled secondary antibody was used for fluorescence detection of the bound primary antibody.

## Supporting Information

Figure S1 Multiple sequence alignment of the Xin repeat region. The 31 conserved GDV(K/Q/R/S)XX(R/K/T)WLFET(Q/R/K/T)PLD Xin repeat units (XR) are individually boxed and numbered within the Xin repeat consensus sequence region for the 40 Xins.
(0.21 MB DOC)

Figure S2 Multiple sequence alignment of the beta-catenin binding domain of Xin proteins. Located within the conserved Xin repeat region, the beta-catenin binding domain previously characterized from aa#535–636 of mXinalpha was used to identify the homologous region in other Xin proteins and aligned with CLUSTAL W. The positions of the 17th–19th Xin repeat units (XR17–XR19) are highlighted above the aligned sequences. Identical residues are highlighted in black with white letters, conserved residues are highlighted in dark grey with white letters, and similar residues are highlighted in light grey with black letters.
(0.05 MB DOC)

Figure S3 Multiple sequence alignment of the Mena/VASP-binding domain and putative DNA-binding domain sequences. The Mena/VASP-binding domain (EDLPLPPPPALED) was previously mapped to aa#18–46 of hXinalpha and two acidic residues each at the N- and C-termini were essential for its binding activity. Based on this characteristic, the Mena/VASP-binding domain is a derived trait found only in the placental mammal Xinα lineage that seems to have arisen from a deletion within the terrestrial Xinα N-terminal proline-rich region. Meanwhile, the putative DNA-binding domain (#47–69 of mXinalpha) identified by the high similarity with the Myb-A and Myb-B DNA-binding domains [1] appears to be an ancestral trait that is highly conserved in most Xin proteins, except Xt Xinalpha28, Tn Xinbeta2, Tn Xinbeta3 and Tr Xinbeta36. Identical residues are highlighted in black with white letters, conserved residues are highlighted in dark grey with white letters, and similar residues are highlighted in light grey with black letters.
(0.05 MB DOC)

Figure S4 Multiple sequence alignment of the C-terminal region immediately downstrean of the Xin repeat region. The entire C-terminal region of the Xin proteins (sequence immediately after the Xin repeats) was aligned to identify regions of high conservation. From this alignment, the sequence region containing aa#743–1,083 of mXinalpha and aa#1,307–1,705 of mXinbeta and their homologous regions from all Xins revealed a high conservation among all the Xinβ proteins and the putative ancestral Pm Xin. There is a high degree of similarity in this region among mammalian Xinalpha proteins, which is much divergent from the same region of all Xinbeta proteins. Interestingly, there is a G(D/N)(V/I/L) motif repeated 10 and 13 times within the conserved C-terminus of all Xinβ proteins and the teleost Xinβ respectively (indicated by * below the alignment). Unexpectedly, this G(D/N)(V/I/L) motif is very similar to the first three amino acids of the Xin repeat unit.
(0.22 MB DOC)

Figure S5 Multiple sequence alignment of the C-terminal proline-rich regions of the Xins. The amino acids residues from #1,245 to 1,334 of mXinalpha and from #1,961 to 2,069 of mXinbeta are highly enriched in proline and then defined as the C-terminal proline-rich regions. Although both Xinalphas and Xinbetas contain this C-terminal proline-rich sequence, the two regions are highly divergent. (A) Sequence alignment of the C-terminal proline-rich region of Xinalphas. A high sequence homology was observed among all mammalian Xinalphas. (B) Sequence alignment of the C-terminal proline-rich region of Xinbetas. A high sequence homology was observed among all mammalian Xinbetas. The conserved proline residues are indicated with a star (*) at the bottom of the alignments.
(0.09 MB DOC)

Figure S6 Multiple sequence alignment of the filamin c-binding region of Xinalpha. The filamin c-binding region was previously mapped to the last 158 amino acid residues (aa#1,685–1,843) of one of spliced variant from hXinalpha gene [10], which is the largest but minor Xinalpha in the human heart, equivalent to mXinalpha-a isoform in the mouse heart. Although the major isoform from hXinalpha and mXinalpha did not contain this filamin c-binding region, we have recently shown by yeast 2-hybrid assay that mXinalpha is able to interact with a more ubiquitous isoform of filamin, filamin b. An alignment of all Xins putative filamin c-binding region did not result in overall conservation (alignment not shown). However, the strongest identity to each other was observed throughout the entire filamin c-binding region of all mammal Xinalphas. Thus, the filamin c-binding region appears to be a derived trait in the mammal Xinalpha.
(0.05 MB DOC)
