# Supplementary material for: Emergence of Xin Demarcates a Key Innovation in Heart Evolution
Source: PLoS One. 2008 Aug 6;3(8):e2857. doi: 10.1371/journal.pone.0002857 (PMC2478706; doi:10.1371/journal.pone.0002857)
Supplement: Figure S1 — Multiple sequence alignment of the Xin repeat region. The 31 conserved GDV(K/Q/R/S)XX(R/K/T)WLFET(Q/R/K/T)PLD Xin repeat units (XR) are individually boxed and numbered within the Xin repeat consensus sequence region for the 40 Xins. (0.21 MB DOC) [file pone.0002857.s001.doc]

**XR3**

**XR2**

**XR1**

*Hs* Xinα .....................................GDVQCMRWIFENWRLDAIGEHERPA............AKEPVLCGDVQATSRKFEEGSFAN.......STDQEPT.RPQPGGG:151

*Pt* Xinα .....................................GDVQCMRWIFENWRLDAIGDHERPA............AKEPVLCGDVQATSRKFEEGSFAN.......STDQEPT.RPQPGGG:151

*Mam* Xinα .....................................GDVQCMRWIFENWRLDAIGDHERPA............AKEPVPGGDVQATSRKFEEGSFAN.......STDQEPT.RPQPSGG:151

*Cf* Xinα .....................................GDVQCMRWIFENWRLDAIGDRERPP............AREPVPGGNVQATSRKFEEGSFAN.......STEQEPA.GPPPSGG:151

*Ec* Xinα .....................................GDVQCMRWIFENWRLDAIGDHEKPP............AREPVPGGNVQATSRKFEEGSFAN.......SIDQEPA.GPRPSGG:151

*Bt* Xinα .....................................GDVQCMRWIFENWRLDAIGDHEKPP............AKESVPGGNVQATSRKFEEGSFAN.......SINQEPA.GPRPSGG:151

*Mm* Xinα .....................................GDVQCMRWIFENWRLDAIGDHERPA............AREPVSGGNVQATSRKFEEGSFTN.......SSDQEPE.GLRPSGG:151

*Rn* Xinα .....................................GDVQCMRWIFENWSLDAIGDHERPA............GKEPVSGGNVQATSRKFEEGSFTN.......SSDLEP..GPQLSGG:150

*Md* Xinα .....................................GDVQSMRWIFENWSLDAIGEHELPQR.........LAEAEAVPGGDVRATSRKFEEGSFA........AKDPAPA.KPGPIQG:142

*Gg* Xin .....................................GEVQSMRWIFENWALDSIGDHQ..AT.......KKMMEDEIIPGGDVKSTSLRFENQSVNGDYLSTTAKVSETDL.....ARG:176

*Ac* Xinα .....................................GEVQSMRWIFENWTLDSIGEHQ..AA.......KKLAEEEAIPSGDVKNRSMRFESPLPNGN..GPSAKVSETEQ.....TKG:181

*Xt* Xinα .....................................GEVQSMRWIFENWNLDAIGEHQ..GS.......KKLVDEEGVLGGNVKDTSMFFKGQPSEGG...LPESLPGKDQ.....TKG:89

*Tn* Xinα15 .....................................GEVQSMKWIFENWNLDNIGEPH.ET........KKLLYEEELRGGDVRGTSSMFEHAGNAQQRSAERQASVR..........G:167

*Tr* Xinα296 .....................................GEVQSMKWIFENWNLDNIGEPHET........ KKLLYDEELKGGDVRGTSSKFEHVENAQQRSTERQAS..........VRG:167

*Ga* Xinα3 .....................................VEVHSMRWIFENWTLDNIGDPH.AT........KKLLDGEELKGGNVRDTSSMFESIDSTQHMSAKRQTSVR..........G:169

*Ol* Xinα17 .....................................GEVQSMKWIFENWTLDNIGDPH.ET........RKLLLEEELKGGDVRNTSLVFEGNDSRVLKPVERQTSVM..........G:164

*Dr* Xinα2 .....................................GEVQSMRWIFENWTLDNIGDPH.ET........KKLLCEENPQGGDVKGKSSLFEHSTFDSQHAAGAERAGVVR........G:169

*Hs* Xinβ ADVQQARYVFENTNDSS..QKDLNSEREYLEWDEILKGEVQSIRWIFENQPLDSINNGSPDEGDI....SRGIADQEIIAGGDVKYTTWMFETQPIDTLGAYSSDTVENAEK.IPELARG:649

*Pt* Xinβ ADVQQARYVFENTNDSS..QKDLNSEREYLEWDEILKGEVQSIRWIFENQPLDSINNGSPDEGDI....SRGIADQEIIAGGDVKYTTWMFETQPIDTLGAHSSDTVENAEK.IPELARG:580

*Mam* Xinβ ADVQQARYVFENTNDSS..QKDLNSEREYLEWDEILKGEVQSIRWIFENQPLDSINNGSPDEGDI....SRGVADQEIIAGGDVKYTTWMFETQPIDTLGAHSSDTVENAEK.IPELARG:655

*Cf* Xinβ ADVQQARYVFENTNDSS..QKTLNSEREHLEWDEILKGEVQSMRWIFENQPLDSINNGSPDEDNI....NKGIAGQEIIAGGDVKYTTWMFETQPIDTLGDHSSGTEENAEK.IPELARG:468

*Ec* Xinβ ADVQQARYVFENSNDSS..QKHLNSEREHLEWDEILKGEVQSMRWIFENQPLDSINNGSPDEDHI....SKGIADQEIIAGGDVKYTTWMFETKPIDTLGDHSFATEENAEK.IPELARG:420

*Bt* Xinβ ADVQQARYVFENTNDSS..QKGLNSEREHVEWDEILKGEVQSMRWIFENQPLDSINNGSPDEDNI....SKGIADQEIIAGGDVKYTTWMFETQPIDTLGDHSSGTEKHAEK.IPELARG:468

*Mm* Xinβ ADVQKARYVFENTNDSS..QKDLNSERENLEWDEILKGEVQSIRWIFENQPLDSINHGSTDEGYT....SKGIADQELIAGSDVKYTTWMFETQPIDALGIPSAGTEGNTEK.IPELARG:420

*Rn* Xinβ AGVQQARYVFENTNDSS..QKDLSSERENLEWDEILKGEVQSIRWIFENQPLDSINQGFTDEAYT....SKGIADQELIAGGDVKYTTWMFETQPIDALGVPSAGTEENTEK.IPELAKG:418

*Md* Xinβ ADVQQTRYVFENTGDSQ..QKCLNPEREYLEWDEILKGEVQSMRWIFENQPLDSIKDQSPDEGNG....KKSIADQEIIAGGDVKYTTWMFETQPIDTLGFHSSGSPESNEK.VPELARG:466

*Ac* Xinβ GDVRQARYIFENSESSP..QKCMSPEREYLEWDEILKGEVQSMRWIFENQPLDLIRDESPDPND.....VKSIGDQEIIAGGDVKYTTWMFETQPMDSLGAHTSESPENADG.VPELACG:328

*Xt* Xinβ GDVQQVKYAFENPVGSP..QKCISPEREYLEWDEILKGEVQSMRWVFENQPLDSIKDESPEPSH.....IKRIGEQEIIAGGDVKYATWMFETKPIDALGVDISTATENIEK.VPDLARG:418

*Tn* Xinβ2 ........................................................................................................................:0

*Tr* Xinβ46 NKLHSCEYIYEDDVVH.....DDINYDQCEEWEEILPGDVQSMRWIFENKPLDTIRDESRSEEDD....EGKITQQEIILGKDVKRTAWMFETKPMDELSTRNISMTEYKNK.FNKIDKG:467

*Ga* Xinβ16 NQSESKEFMHEDDAGGP....DDVNDEEYVEWEEILPGEVQAMRWKFENKPLDTIKDEA............QITEQEMILGKDVRRTAWMFETKPMDELTSHNINSTEYKNK.FNKCDKG:382

*Ol* Xinβ21 NQVQRQVCAYEDDGGSP....EDIYDEEYEEWEEIVPGEVQAVRWMFENKPLDAIKDETPEEDEQ....NKRFTEQEMIWGKDVRRTAWMFETKPMDELGSVNTNSTDYKNK.FNKLEKG:268

*Dr* XinβNA GDVQEAAYQFEHTGRNS....NGSPDREYLEWDEILRGEVQSMRWMFENKPLDCIKDDSDNEEE.....SRKIVQQEFVAGGDVRNTALMFEMQPIDEVGQKRDSKRNYL...VNEVGKR:283

*Tn* Xinβ3 ........................................................................................................................:144

*Tr* Xinβ36 GDVQQACYMFENDCN....SPCSSPDRDSVEWEEILKGEVQSVRWMFENKPLDTIKDESPDESE.....GKSIAHQEIIAGKDVKYTAWIFETQPMDALGTDTAGEAEESQK.SAAMARG:664

*Ga* Xinβ1 GDVQQACYLFENDGNGS..SKCSSPDREYVEWDEILKGEVQSMRWMFENKPLDTIKDDNPGEDQ.....VGNIAQQEIIAGKDVRHTAWMFETRPIDALGTDAVDATGQSQKSTADLARG:442

*Ol* Xinβ2 SEVQQTRFRFENDGDDS..SECSSPDNESVEWDEILKGEVQSRRWMFENKPLDTIKDDSLDEIE.....ERNIAQQEIIAGKDVRHTAWMFETQPIDALGTEASETQDQSEK.QNDLARG:391

*Dr* Xinβ6 RDIQQARYVFENSGSTSP.GKCISPEREYMEWDEILKGEVQSMRWMFENKPLDSIKDVTPDEDE.....GKNIAQQEIIAGKDVKYTMWMFETQPIDALGTEKSDSAEHGGK..HELARG:717

*Dr* Xinβ19 RDIQQARYVFENSGSTSP.GKCISPEREYMEWDEILKGEVQSMRWMFENKPLDSIKDVTPDEDE.....GKNIAQQEIIAGKDVKYTMWMFETQPIDALGTEKSDSAEHGGK..HELARG:459

*Pm* Xin GDPQQARWLYEQGLAGPSPPPPPQGDRECLEWDEILRGEVRSMRWMFETQPLDTIREPGTPDPNAGGSGGGGGGGGDPIPCGDVRYTAWKFETQPLDSLAVHSADKATKDAR....AGKG:271

**XR6**

**XR5**

**XR4**

*Hs* Xinα DVRAARWLFETKPLDELT....GQAKELEATVREPAASGDVQGTRMLFETRPLDRLGSRPSLQEQSPLELRSEIQELK.GDVKKTVKLFQTEPLCAIQDAEGAIHEVKAACREEIQS...:263

*Pt* Xinα DVRAARWLFETKPLDELT....GQAKELEATVREPAASGDVQGTRMLFETRPLDRLGSRPSLQEQSPLELRSEIQELK.GDVKKTVKLFQTEPLCAIQDAEGAIHEVKAACREEIQS...:263

*Mam* Xinα DVRAARWLFETKPLDELT....GQAKELEATVREPAASGDVQGTRMLFETRPLDRLGSRPSLQEQSPLELRSEIQELK.GDVKKTVKLFQTEPLCAIQDAEGAIHEVKAACREEIQS...:263

*Cf* Xinα DVRAARQLFETKPLDELT....GQAEALEAAVKEPEASGDVQGTRMLFETRPLDRLGSRPSIQEQSPLELRSEIQELK.GDVKKTVKLFQTEPLCAIQDAEGAIHEVKAACREEIQS...:263

*Ec* Xinα DVRAARWLFETKPLDELT....GQTGAPEATVREPAASGDVRGTRMLFETRPLDRLGSRPSIQEQSPLELRSEIQELK.GDVKKTVKLFQTEPLCAIQDAEGAIHEVKAACREEIQS...:263

*Bt* Xinα DVRAARQLFETKPLDALT....VRAEASEATVREPAASGDVQGTRMLFETRPLDRLGSRPSTQEQSPLELRSEIQELK.GDVKKTVKLFQTEPLCAIQDSEGAIHEVKAAYREEIQS...:263

*Mm* Xinα DVQAARQMFETKPLDALR....GQEEATQTTMREPAATGDVQGTRKLFETRPLDRLGSRPSIQEQSPLELRSEIQELK.GDVKKTVKLFQTEPLCAIQDAEGTIHEVKAACREEIQS...:263

*Rn* Xinα DVRATRQLFETKSLDALT....GQEEATQMTMKEPAVSGDVQGTRKLFETKPLDMLGSCPSIQDQSPLELRSEIQELK.GDVKKTVKLFQTEPLCAIQDAEGAIHEVKAACREEIQS...:262

*Md* Xinα DVQAARWLFETQPMDTLT....GQTDIQKAVLREAPPRGDVRSTRTLFETRPLDSLGRRASAQEQSPLQLRSEIQELK.GDVKKTVKLFQTEPLCAIQDAEGGIHEVKAACREEIQS...:254

*Gg* Xin DVHTARWLFETQPLDSLNKLYSDETEMQEAVLKEPVQGGDVKGAKELFEAQSLDAIGRCCSVEEKSILQLKSEIQELK.GDVKKTIRLFQTEPLCAIRDKTGNIHEIKSVCREEIQS...:292

*Ac* Xinα DVRTARWLFETQPLDALNKMYLDETDLQEAVLKEPVEKGDVKGAAQLFETHSLDALGRCCSVEEQSILQLKSEIQEHK.GDVKKTIKLFQTEPLCAIRDQTGHIHEIKSVCREEIQS...:297

*Xt* Xinα DVKTALWLFETQSLDSMNKIYPEDTEVQEAILKEPVERGDVKSTKHLFETCSLSEVGRCNSVEENSILQLKSEIQELK.GDVRKTVKLFQTEPLCAIRDDNGNIHEIKSICREDTEA...:205

*Tn* Xinα15 DVRTSMWLFETQPLDVLNRRNSEEGEMVEAVLKEPIQLGDVRGTRQLFESRPLSDLGLCDSVDDHHVLKLRSELQEQR.GDIQKTLKVFQAEPHCAIRDNSGNIHEIRSICREEING...:283

*Tr* Xinα296 DVRTSMWLFETQPLDALNLLNREEGEMVEAVLKEPIQPGDVRGTRQLFESRPLSDLGRCGSIEDHSVLKLRSELQEQK.GDIQKTLKVFQSEPRCAIRDNSGNIHEIKSICREEING...:283

*Ga* Xinα3 DVRTSKWLFETQPLDSLNKSKRDEGELVEAVLKEPIQPGDVTGARLLFESKPLSDFGRCDSIEDHSFLKLRSELEEQK.GDVQKNLKLFQADPCCAIRDNSGNIHEIKSICREEINS...:285

*Ol* Xinα17 DVRTSMWLFETQPLDSFNKPKTEEGELVEALLREPIQPGNVRGTRLLFESKPLSDLGQCNSCEDHTFLKLKSELQEQK.GDVQKTVKLFQTDPGCAIRDNSGNIHEIKSICREEINS...:280

*Dr* Xinα2 DVRTATWLFETQPLDSISKSKIEDEEIVEVVLKEPVQKGDVTGARRLFETKPLDSLGRCCSVEDQHFLTLKSELQENK.GDVKKTVKLFQADPCCALRDSNGKIHEIKSICREEIMS...:285

*Hs* Xinβ DVCTARWMFETRPLDSMNKMHQSQ.EESAVTISKDITGGDVKTVRYMFETQHLDQLGQLHSVDEVHLLQLRSELKEIK.GNVKRSIKCFETQPLYVIRDGSGQMLEIKTVHREDVEK...:764

*Pt* Xinβ DVCTARWMFETRPLDSMNKMHQSQ.EESAVTISKDITGGDVKTVRYMFETQHLDQLGQLHSVDEVHLLQLRSELKEIK.GNVKRSIKCFETQPLYVIRDGSGQMLEIKTVHREDVEK...:695

*Mam* Xinβ DVCTARWMFETRPLDSMNKMHQSQ.EESAVTISKDITGGNVKTVRYMFETQHLDQLGQLHSVDEVHLLQLRSELKEIK.GNVKRSIKCFETQPLYVIRDGSGQMLEIKTVHREDVEK...:770

*Cf* Xinβ DVRTARWMFETKPLDSMNKMHQ...EESMVTATKDITGGDVKTVRYMFETQHLDQLGQLHSVDEVHLLQLRSELKEIK.GNVKRSIKCFETQPLYVIRDGLGQMLEIKTVHREDVEK...:581

*Ec* Xinβ DVRTARWMFETNPLDSMNKMHQSQ.EELVVPTIKDITGGDVKAVRYMFETQHLDQLGQLHSVDEVHLLQLRSELKEIK.GNVKRSIKCFETQPLYVIRDGLGQMLEIKTVHREDIEK...:535

*Bt* Xinβ DVHTARWMFETKPLDSMNKLHQSQ.EESIATAIKDITGGDVKTVRYMFETQHLDQLGQLHSVDEMHLLQLRSELKEIK.GNVKRSIKYFETQPLYVIRDGSGQMLEIKTVHREDVEK...:583

*Mm* Xinβ DVYTARWMFETRPLDSMNKMHECQ.EETASTLTKDITGGDVKTVRYMFETQQLDQLGQLHSVDELNLLQLRSELKEIK.GNVKRSIKCFETQPLYVIRDGSGQMLEIKTVQREDIEK...:535

*Rn* Xinβ DVCTARWMFETRPLDSMNKMHEWE.DETASTFIKDITGGDVKTVRYMFETQQLDQLGQLHSVDEMNLLQLRSELKEIK.GNVKRSIKCFETQPLYVIRDGSGQMLEIKTVQREDIEK...:533

*Md* Xinβ DVRTATWMFETQPLDSMNKIYQDQQEGSEGIAIKDITGGDVKTVKYLFETQNLDQLGQLYSVDEANLLQLRSELKEIK.GNVKRSIKHFETQPLYVIKDDLGQILEIKTVHREDIEK...:582

*Ac* Xinβ DVRTATWMFETQPLDSMNKIHHTK.EDSDESCFKEITGGDVKTVKYMFETQHLDSLGQLYSVDETKLLQLRSELKEIK.GDVKRSIRHFETLPMYVIRNNLGQMLEIKTVQREDLER...:443

*Xt* Xinβ DVRTATWLFETQPLDCLNKIYKEDDLSNTLETTEEIKGGDVKLGKHLFESTFQN.........EISSLRLSSELEELK.GDVKTTIKQFETEPKYVLKDSSGKMLEIKTIRREDVEK...:525

*Tn* Xinβ2 ........................................................................................................................:0

*Tr* Xinβ46 DVRAAAWLFETHRMDALNKMHKEEDLTKEILFT....ENDENSTIYMIDNKCMENLGHTETIDESHLLTLRSVLEEIK.GEVKTVTTTFDTQFKCVIMGQSSQLLEIKSVRKTETEL...:579

*Ga* Xinβ16 DVRAAAWLFETQTMDTLNKMHKEEDLTKEIVFT....EEDGNATIYMIDNKYMESLGHTETIDESHLLTLRSVLEEIH.GEVKTVTSTFDTQFKCIIMGQSSQMLEIKSVRKIESEL...:494

*Ol* Xinβ21 DVRAAAWLFETQTMDTLNKMHKEEDLTKEIVFT....EEDGNATIYMIDNRFVEGLGHTETVDESRLLSLRSVLEEIN.EEVKTVTTTFDTQFKCIIMGQSSQMLEVKSVRKIETEL...:380

*Dr* XinβNA DVRAAAWLFETKPMDTLNKLHTDDEQTKEVIFTHEATEGDMKSVRYIFENQEMDSLGDTETMDEKQLLSLKSVLEEIK.SEVKKIIWMFDTQCMCVMREHSGEMVLITSVRREETEK...:399

*Tn* Xinβ3 ............PLDCLNKIYQEDEQVADVVTTRDIAGGDVKTARYLFETQHLDSFGKTETIEESHFLNLKSELEEIK.GDVKTTTRMFETQPMCVIRGDSGEILEVTTISREETEK...:248

*Tr* Xinβ36 DVRTATWLFETQPLDCLNKIYHEDEQEADVVVTRDIAGGDVKTARYLFETQHLDSLGKTETIKESHFLNLKSELEEIK.GDVKTTTRKFETQPMCVIRGDSGEMLEVTTVRREETEK...:780

*Ga* Xinβ1 DVRTATWLFETQPLDYLNKIYQEDEQETAVVVNRSITGGDVKTARYLFETQHLDSLGKTETIEESNFLNLKSELEEVK.GDVKTTTRMFETQPMCVIRGDSGEMLEITTIRREETEK...:538

*Ol* Xinβ2 DVRTATWLFETQPLDYLNKIYQEDEQDSDATN..DITGGDVKTARYLFETQHLDSLGKSETIEESSFLSLKSELEEIK.GDVTTTTRMFETFPMCVIRGDSGEMLEITTIRREETEK...:505

*Dr* Xinβ6 DVQTATWLFETQPLDTLNRIYQEDDQETQVMYTKDIAGGDVKTARYLFETQHLDALGHTETIDENHFLQLKSELEEVK.GEVKKTTKMFETQPMCVIRGDSGEMLEITKVRREETEK...:833

*Dr* Xinβ19 DVQTATWLFETQPLDTLNRIYQEDDQETQVMYTKDIAGGDVKTARYLFETQHLDALGHTETIDENHFLQLKSELEEVK.GEVKKTTKMFETQPMCVIRGDSGEMLEITKVRREETEK...:575

*Pm* Xin DRGRG.....................HEDVRST..VMGGDVSMGRYMFETKPISSLARWEE.EESDLLKLKSELEEILGGDVRRSVQLFESYPLCAIRDSQGHVHRITSVRREEVEECSL:364

**XR8**

**XR7**

**XR9**

*Hs* Xinα ...NAVRSARWLFETRPLDAINQDPSQVRVIRGISLEEG.................................................................................:299

*Pt* Xinα ...NAVRSARWLFETRPLDAINQDPSQVRVIRGISLEEG.................................................................................:299

*Mam* Xinα ...NAVRSARWLFETRPLDAINQDPSQVRVIRGISLEEG.................................................................................:299

*Cf* Xinα ...NAVRSARWLFETQPLDAINRDPSQVRVIRGISLEEG.................................................................................:299

*Ec* Xinα ...NAVRTARWLFETQPLDAINRDPSQVRVIRGISLEEG.................................................................................:299

*Bt* Xinα ...NAVRSGRWLFETKPLDAINRDPSQVRVIRGISLEEA.................................................................................:299

*Mm* Xinα ...NAVRSARWLFETRPLDAFNQDPSQVRVIRGISLEEG.................................................................................:299

*Rn* Xinα ...NAVRSARWLFETRPLDAFNQDPSQVRVIRGISLEEG.................................................................................:298

*Md* Xinα ...NAVRTARWLFETRPLDAISRDPSQVRVIRGISLEEA.................................................................................:290

*Gg* Xin ...NAVRTARWLFETQPLDTINKDTSKVQIIRGISLEEI.................................................................................:328

*Ac* Xinα ...NAVKTARWLFETQPLDTINKDTSKVQVIRGISLEEA.................................................................................:333

*Xt* Xinα ...DNVNRTRWLFETQPLDSIHKNSPKVQIIRGISLEEL.................................................................................:241

*Tn* Xinα15 ...SNFSTARWLFETQPLDLIHEETSGVKIIRGISLEEG.................................................................................:319

*Tr* Xinα296 ...SNFNTARWLFETQPLDLIHEETSGVKIIRGISLEEA.................................................................................:319

*Ga* Xinα3 ...SDISTARWLFETQPLDLINRGTDGVKIIRGISLEEG.................................................................................:321

*Ol* Xinα17 ...SEIHTARWLFETQPLDLINKGVDGVKIIRGISLEEG.................................................................................:316

*Dr* Xinα2 ...SDFKTARWLFETQPLDHINEGAH.VQIIRGISLEEA.................................................................................:320

*Hs* Xinβ ...GDVRTARWMFETQPLDTINKDITEIKVVRGISMEEN.................................................................................:800

*Pt* Xinβ ...GDVRTARWMFETQPLDTINKDITEIKVVRGISMEEN.................................................................................:731

*Mam* Xinβ ...GDVRTARWMFETQPLDTINKDITEIKIVRGISMEEN.................................................................................:806

*Cf* Xinβ ...GDVRTARWMFETQPLDTINKDITEIKVVRGISMEEN.................................................................................:617

*Ec* Xinβ ...GDVRTARWMFETQPLDTINKDITEIKVVRGISMEEN.................................................................................:571

*Bt* Xinβ ...GDVRTARWMFETQPLDTINKDITEIKVVRGISMEEN.................................................................................:619

*Mm* Xinβ ...GDVRTARWMFETQPLDTINKDITEIKVVRGISMEEN.................................................................................:571

*Rn* Xinβ ...GDVRTARWMFETQPLDTIKQDITEIKVVRGISMEEN.................................................................................:569

*Md* Xinβ ...GDVRTARWMFETQPLDMINKDITEVKVVRGISMEEN.................................................................................:618

*Ac* Xinβ ...GDVRTVRWMFETQPLDMINKDSLEIKVVCGISMEES.................................................................................:479

*Xt* Xinβ ...GDVKTARWMFETQPLDTINQDEVEVKVVRGISLEEN.................................................................................:561

*Tn* Xinβ2 ........................................................................................................................:0

*Tr* Xinβ46 ...ENSIASRWLFDTQPLTVTDIGPTPLSLLCSLSMEDSNKGDWGRWLFEIKTLNSLQDLEKPKAENEEVIGADVRKHCLVFETQPMDSLKDDSNARPQTIEDIIGGNVRSARHFFESSP:696

*Ga* Xinβ16 ...ENSIASRWLFDTQPLDTTRRESTSLKLVCSLSME...................................................................................:528

*Ol* Xinβ21 ...ENSIASRWLFDTQPLDTKNRELVPLKLICSLSMEDSSKGDWGRWLFEIKTLNSLSEWESTQLEKTEIIGADVRKHCLVFETQPMDSLKDDSNARPQSLEEIIGGNVRSARNFFESSP:497

*Dr* XinβNA ...GDIKTSRWLFETQSLNDIKMDISQVRLISSVSMEDN.................................................................................:435

*Tn* Xinβ3 ...GDVKTSRWMFETQPLDMINKDPTKIKLICGISVGDN.................................................................................:284

*Tr* Xinβ36 ...GDVKTSRWMFETQPLDMINKDPAKVKLICGISMEDS.................................................................................:816

*Ga* Xinβ1 ...GDVKTSRWMFETQPLDIINKDPAVVKLICGISMEDD.................................................................................:574

*Ol* Xinβ2 ...GDVRTSRWMFETQPLDMINKDPAMVKLICGISQEET.................................................................................:541

*Dr* Xinβ6 ...GDVRTSRWLFETQPLDLISKDPSKVKLICGVSMEEN.................................................................................:869

*Dr* Xinβ19 ...GDVRTSRWLFETPPLDLISKDPSKVKLICGVSMEEN.................................................................................:611

*Pm* Xin GVEGSVKSARWLFETSPLDSINASSSELRIVRGISMEDE.................................................................................:403

**XR12**

**XR11**

**XR10**

*Hs* Xinα .....................ARPDVSATRWIFETQPLDAIREILVDEKDFQP......SPDLIPPGPDVQQQQHLFETRALDTLKGDEEAGAEAPPKEEVVPGDVRSTLWLFETKPLDA:392

*Pt* Xinα .....................ARPDVSATRWIFETQPLDAIREILVDEKDFQP......SPDLIPPGPDVQQQRHLFETRALDTLKGDEEAGAEAPPKEEVVPGDVRSTLWLFETKPLDA:392

*Mam* Xinα .....................SRPDVSATRWIFETQPLDAIREILVDEKDFQP......SPDLIPPGPDVQQQRHLFETRALDTLKGDEEAGAEAPPKEEVVPGDVRSTLWLFETKPLDA:392

*Cf* Xinα .....................ARPDVSATRWIFETQPLDAIREILVDEKDFQP......SPDLIPPGPDVQQQRHLFETRALDTLKGEEESEAQHPPKEEVVPGDVRSTLWLFETKPLDT:392

*Ec* Xinα .....................VRPDVSAARWIFETQPLDAIREILVDEKDFQP......SPDLIPPGPDVQHQRHLFETRALDTLKGEEEAGTEVPPKEEVVPGDVRSTLWLFETKPLDT:392

*Bt* Xinα .....................ARPDVSATRWIFETQPLDAIREILVDEQDFQP......SPDLIPPGPDVQQQRRLFETRALDTLKGEEEAGAEAPPKEAVVPGDVRSTLWLFETKPLDT:392

*Mm* Xinα .....................ALPDVSATRWIFETQPLDAIREIEVDEKDFQP......SPDLIPPGPDVQHQRHLFETCSLDTLKGERETEAEVPPKEEVIPGDVRSTLWLFETKPLDA:392

*Rn* Xinα .....................ALPDVSATRWIFETQPLDAIREILVDEKDFQP......SPDLIPPGPDVQHQRHLFETCALDTLKGERETEAEAPAKEEVIPGDVRSTLWLFETKPLDA:391

*Md* Xinα .....................ARPDVSNTRWLFETQPLDAIREVTIDEKEFQP......SPDLVPPGPDVQQQRMLFETRALDTLKGE.EAPEDGPPKEEVLGGDVRSTLWLFETQALGA:382

*Gg* Xin .....................GRPDVSGARWIFETQPLDAIREITVEEQDFKA.......STDFVTGADVTKQRLLFGTQALDSLKG..EASESVAAKEQVIGGDVKSTLWLFETQPMET:418

*Ac* Xinα .....................AKGNVSGAKWLFETQPLDAIREITVEESDFKA.......SPDLIQGADVSKQCLLFETQPLDALKG..ESSNSIPTREEVVKGDVKSSLWLFETQPMET:423

*Xt* Xinα .....................EKGGVNAKKWIFETQPLDTIKEN.NEEGMFQA.......SLDSHEGADSSS....FENR.........ECSGNINCTDKVLPGDVKSTLWLFESQPMET:319

*Tn* Xinα15 .....................NRGGVDQKRWMFETQSFDTIQEDAG.AGKFEG......AVAECGDEADVANKRRLFEMQPLAALRG..DSSQESWQKEEIIGGDVKTSLWLFETQPLGT:409

*Tr* Xinα296 .....................NRGGVDQKRWMFETQSFDAIQEDVG.ADKFEG......TMAECAEEADVVNKRRLFEMQPLAALKG..DSTQEPLEKEEIIGGDVKTSLWLFETQPMET:409

*Ga* Xinα3 .....................HRGGVDQKRWMFETQSLDRIQEVYG.VDKFEG......TVANCAGEADVVNKKKLFEMQPLAALKG..DSAEKSLEKEEIIAGDVKTSLWLFETQPMES:411

*Ol* Xinα17 .....................QKGGVDQKRWMFETQSFETIQEAIG.ADKLEA......QVAICPGEA.VSNKKKLSERQPLVSLKG..DAAETSVEKEEVIGGDVKTSLWLFETQPMEA:405

*Dr* Xinα2 .....................QRGGVDKKKWMFETQPLDAIHEGVVEEQKFQGT.....AVEGFSGAADVHNKLQLFENQPLSSLKG..DSEGDVLEKEAIVGGNVGSTLWLFETQPMDT:412

*Hs* Xinβ .....................VKGGVSKAKWLFETQPLEKIKE.SEEVII..........EKEKIIGTDVSRKCWMFETQPLDILKE..VPDADSLQREEIIGGDVQTTKHLFETLPIEA:886

*Pt* Xinβ .....................VKGGVSKAKWLFETQPLEKIKE.SEEVII..........EKEKIIGTDVSRKCWMFETQPLDILKE..VPDADPLQREEIIGGDVQTTKHLFETLPIEA:817

*Mam* Xinβ .....................VKGGVSKAKWLFETQPLEKIKE.SEEVII..........EKETIIGTDVSRKCWMFETQPLDILKE..VPDADPLQHEEIIGGDVQTTKHLFETLPIEA:892

*Cf* Xinβ .....................VKGGVSRAKWLFETQPLEKIKEESEEVII..........EKETIIGTDVSRKCWMFETQPLDILKD..VPDTEPLRSEEIIGGDVQTTKHLFETLPIEA:704

*Ec* Xinβ .....................VKGGVSRAKWLFETQPLEKIKEESEEVIS..........EKETIIGTDVSRKCWMFETQPLDILKE..VPDADAVRSEEIIGGDVQTTKRLFETLPIEA:658

*Bt* Xinβ .....................VKGGVHRAKWLFETQPLEKIKEETEEVIV..........EKETVIGTDVSKKCWMFETQPLDILKE..VPDADHLKSEEIIGGDVQTTKQLFETLPIEA:706

*Mm* Xinβ .....................VKGGVSRAKWLFETQPLEKIKEESGEAVL..........KTEAVIGTDVSKKCWMFETQPLDILKD..SPDTDSVSPEERIGGDVKTTKHLFETLPIEA:658

*Rn* Xinβ .....................VKGEVGRARWLFETQPLEKIKEESGEAVL..........KTEAVVGIDVSKKCWMFETQPLDTLKQ..SPDTESVSPEERIGGDVKTTKHLLETLPIEA:656

*Md* Xinβ .....................IRGGVSKAKWLFETQPLESIKEESEVSVI..........EKETIIGTDVSRKCWMFETHPLDTLKE..VTDSNPLPAEEIIGGDVKATKYLFETLPMDI:705

*Ac* Xinβ .....................IKGEVGRAKWLFETQPLDTIKQEYEETVV..........EKEAILGTDVCKKCWLFETQPLDTLKE..SEDANPLPSEEIIGGDVNTTKHLFETLPMDR:566

*Xt* Xinβ .....................LTGGVGKAKWLFETQALDTIKE.VESSVT..........EKETIIGSDVYQKCWVFETIPMDMLKD..NANERPMQGEEIIGGDVSNTKYLFETVPMDE:647

*Tn* Xinβ2 ..........................................................MQENGTSCRADVRKNCWVFETQPMDTLKD..DSNTRPVTKEEIIAGNVRSARHYFETIPAEE:60

*Tr* Xinβ46 QAEGKTGLEVGKLQKVTLAEEAKGDVRHQKWRFESQPLEHIGEQKKEVIRTVNVEEDLTQEDGTSCRADVRKNCWVFETQPMDTLKD..DSNTRPVTKEEIVAGNVSSARHYFETTPAEE:814

*Ga* Xinβ16 ...................EEIKGDVRHQKWRFESQPLEHIREEKK.............EEDGTSCRADVRKNCWVFETRPMDTLKD..DSNTQLLTKEDIIAGNVSSARHYFETAPAEE:614

*Ol* Xinβ21 QVARKNYQEVGKLKKATANEEMKGDVRHQKWRFESQPLDHIREEKKEVVRTVNVDEDLTQEDGSSCRADVRRNCWVFETQPMDTLKD..DSNTRVMTKEEIIAGNVQSARQYFETSPTEE:615

*Dr* XinβNA ....................HMG.DVGHQRWLFENQRLEDIQEEKKVFMKTID.....SEQIDKSYKGDVRRNCWVFETQPMDTLKD..DSNARPASAEEIIGGDVQSARHFFETAPKDE:527

*Tn* Xinβ3 .....................VECGVNRGTWLFETKTLDTIKDEEWECSR.........KQKEEIIGADVRKHCLVFETQPMDTLKD..NANARPVAVEEIVGGNVQSAKHLFETGPMEN:372

*Tr* Xinβ36 .....................VECGVNRGTWLFETKTLDTINDEEWESSR.........KQKEEIIGADVKKHCQVFETQPMDTLKD..NANARPVTTEEIVGGDVRSAKHLFESAPAEN:904

*Ga* Xinβ1 .....................VQVGVNKGRWLFETKTLDTIRDEEWESSR.........KQKEDIIGTDVRKHCLVFETQAMDTLKD..NANARPLPSEEIVGGDVQTAKHLFETVPMEN:662

*Ol* Xinβ2 .....................TQGGVNRGRWLFETKTLDSIKDEEWESSM.........SQRKDIIGADVRKRCLVFETQPMDTLKD..NTNARPLPSEEIVGGDVKSAKHLFETVPMEN:629

*Dr* Xinβ6 .....................SQGGVNRGRWLFETKTLDQIKEEEWETTK.........TQKEEILGADVRRHCMIFETQPMDKLKD..DTNARPLEPENIMGGDVTSARQLFETVPMED:957

*Dr* Xinβ19 .....................SQGGVNRGRWLFETKTLDQIKEEEWETTK.........TQKEEILGADVTRHCMIFETQPMDKLKD..DTNARPLEPENIMGGDVTSARQLFETVPMED:699

*Pm* Xin .....................QKGGVGRARWLFENQSLDTINEDTEGGEYT.......KVQKEVIEGSDVKRHRWLFETHTMDTLKD..NADEDRQQKEEVIGGDVKSSLWLFETRPMDS:493

**XR14**

**XR15**

**XR13**

*Hs* Xinα FRDKVQVGHLQRVDPQDGEG.............HLSSDSSSALPFSQSAPQRDELKGDVKTFKNLFETLPLDSIGQGEVLAHGSPSREEG..TDSAGQAQG.....IGSPVYAMQDSKGR:492

*Pt* Xinα FRDKVQVGHLQRVDPQDGEG.............HLSSDSSSALPFSQSAPQRDGLKGDVKTFKNLFETLPLDSIGQGEVLAHGSPSREEG..TDSAGQAQG.....IGSPVYAMQDSKGR:492

*Mam* Xinα FRDKVQVGHLQRVDPQDGEG.............HLSSDNSSALPFSQSAPQRDGLKGDVKTFKNLFETLPLDSIGQGEVLAHGSPSREEG..TDSAGQAQG.....IGSPVYAMQDSKGR:492

*Cf* Xinα PRDKVQVGHLQRVGPRDHEGL.........ISEHLSRDGSSAASHSQSAAQRDGVKGDVKAFKNLFETLPLDSIGQGEPSAHRSMNRAEG..TDSTGQSQD.....VGSPVYAMQDGKGH:496

*Ec* Xinα PRDKVQVGHLQRVGPRKGEGL.........MYEHPSSDGSSALSLSQSAPQRDGVKGDVKTFKNLFETLPLDSIGQGEPLAHGSVNRAEG..TDSAGQSQD.....IGSPVYAMQDGKGH:496

*Bt* Xinα LRDNVQVGHLQRVGPQEGERF.........TNEHLSNADPSAPTLSQGAPQRDGVKGDVKTFKNLFETLPLDSIGQGEALAPGSICRAEG..TDSAGQSQD.....TGSPVYAMQDGKGH:496

*Mm* Xinα FRDQVQVGHLQRVGHQEGEGL.........VTECLPSNGTSVLPLSQGVPQNDGLKGDVKTFKNLFETLPLDSIGQGEPSAYGNINRGQN..TDSAEQSQG.....SDAPVYAMQDSRGQ:496

*Rn* Xinα FRDQVQVGHLQRVDHQKGEGP.........VIECLSSNGTSALPLSQGVPQNNGPKGDVKTFKNLFETLPLDSIGQGEPSACGNINRGQR..TDSTGLSEG.....ADAPVYAMQDSRGQ:495

*Md* Xinα PSREVQVGRLQQVRQGESENGDVCAP....ELENRASIGASKVALLPFPAGDQGGKGDVKAFKKLFETLPLDSIGQERPPGPAEDRGLQG..VVPAGQVQAGQALFEEWPLYAIQDGHGG:496

*Gg* Xin LKDNVEVGHLKKVELSAEEKGDVKQRKHVFETCPLGSISKAFEEEISAASTEEVVKGDVKSFKTLFETLPLDSIKEVD.....AEPITKEEEKIPPGNVKANQILFETTPLYAIKDSFGN:533

*Ac* Xinα LRDSYEVGHLMKVELTDEEKGAVKQTKHVFETCPLGSIAKSPAGDISTTNIQEVEKGDVKAFKSLFETLPLDSIRQVS.....SEEMVEKSEEIPPGNVKANQILFETTPLYAIKDCFGN:538

*Xt* Xinα LKGNFEVGPLKKVEMLNEEKGDVKQRKHLFETCSLDKISD..KDEEAVRNVTEISKGDVTTFKNLFETLPLESFEKTS.....NVTVSDQ.EDIQVGNVKANQTLFETVPLYAIEDSLGN:431

*Tn* Xinα15 LNDHWEVGHLKKITLSAGEQGEVKDKRQIFESCSSEKKAS........FQHHNVEKGDVKGFKRLFETIPLSKIAQS......DEEAVKA......EKNDGANLMCETTPLYAIQDGSGG:509

*Tr* Xinα296 LKDNYEVGHLKKITLSADEQGEVKGKKQIFESCHTEKNTS........FQQDKIEKGDVKGFKHLFETIPLSKLAHV......NEELVEM......EKVDAANIRCETTPSYVIKDYSGN:509

*Ga* Xinα3 LNDNYEVGRLKKVTLSSDEQGEVKGKKQMFESCTIPKKTS........SKEQEFEKGDVKGFKHLFETLPLSKISQS......DEEFTEGEEVAAAVDVKGTKAMFETTPVYAIKDSSGN:517

*Ol* Xinα17 LGENDEVGRLKKITISAEEQGGVKDRKQIFQNGGMHRDVL........PKKQVIEKGDVKGFKQLFETILLSKSAES......QESFAIMKTIEG.GNVKENKLMFETTPLYAIKDSSGN:510

*Dr* Xinα2 LKDSYEVGRLQKVMVSSDEKGEVQDKRMQFEKSTAGKTAGD.....SGNKVQNDEKGDVKTFKSLFETLPLNVSEKA......QSQIHDITS....GDVKGHCSLFETTPLYAIKDCAGK:517

*Hs* Xinβ LKDSPDIGKLQKITASEEEKGDVRHQKWIFETQPLEDIRKDKKEYTRTVKLEEVDRGDVKNYTHIFESNNLI.KFDAS.....HKIEVEG...VTRGAVELNKSLFETTPLYAIQDPLGK:997

*Pt* Xinβ LKDSPDIGKLQKITASEEEKGDVRHQKWIFETQPLEDIRKDKKEYTRTVKLEEVDRGDVKNYTHIFESNNLI.KFDAS.....HKIEVEG...VTRGAVELNKSLFETTPLYAIQDPLGK:928

*Mam* Xinβ LKDSPDIGKLQKITASEEEKGDVRHQKWIFETQPLEDIRKDKKEYTRTVKLEEVDRGDVKNYTHIFESNNLI.KFDAS.....HKIEVEG...VTRGAVELNKSLFETTPLYAIQDPLGK:1003

*Cf* Xinβ LKDSPDVGKLQKITASEEEKGDVRHQKWIFETQPLEDIREDKKEYIRTVKLEEVDRGNVKNFTHIFESNNLI.KFDAS.....HKIEVEG...VTRGAVELNKSLFETTPLYAIQDHLGK:815

*Ec* Xinβ LKDSPAVGKLHKITASEEEKGDVRHQKWIFETQPLEEIREDKKEYIRTVKLEEVDRGDVRNYTHIFESNNLI.KFDAS.....HKIEVEG...VTRGAVELNKSLFETTPLYAIQDHLGK:769

*Bt* Xinβ LKDSPDVGKLQKITASEEEKGDVRHQKWIFETQPLEEIREDKKEYIRTVKLEEVDRGDVRNYTHIFESNNLI.KFDAS.....HKIEVEG...VTRGAVELNKSLFETTPLYAIQDHLGK:817

*Mm* Xinβ LKDSPDIGKLQKITASEEEKGDVKHQKWVFETQRLEDIREDKKEYTRTVRLEAVDRGHVKNYTHIFESNNLI.KVDAS.....HQIEVEG...VTRGTVELNKSLFETTPLYAIQDHLGK:769

*Rn* Xinβ LKDSPDVGKLQKITASEEEKGDVKHQKWVFETQRLEDIREDKKEYTQTVKLEAVDRGHVKNYTHIFESNNLI.KVDAS.....HQIEVEG...VTRGTVELNKSLFETTPLYAIQDHLGK:767

*Md* Xinβ LKDSPEVGKLQKIAASEEEKGDVRHQKWVFETQPLENIREGEKEYIKTVKLEEIDKGDVRSYTHIFESNNLI.KFDES.....HKIQVEG...VTRGAVEYNKSLFETTPLYAIQDHLGR:816

*Ac* Xinβ LKDSPDVGKLQKMAATEEEKGDVRHQKWIFETKPLEQIREEQKEIIRAVKLEEIDKGDVSSCRHVFETCHLR.KQDDS.....YKIHVEG...VTQGAVKLNKAVFETTPLYAIQDRLGK:677

*Xt* Xinβ LKESVEVGKLKPIVTMEDEKGDVRHQRWVFETKSLGEIGEDHKEHVRTVQLDEIHKGDVISCKNAFEKGDLT.KSELT.....YKIAVED...VDKGAVKLNKKLFETTPLYAIQDRFGH:758

*Tn* Xinβ2 VKELTEVGKLKKSVALEEERGDVRHQKWRFESQTLEEIREEKKDCLRTVDLNETDKVDIQNVKHIFESTDSS.GGEKS.....HKILIEG...VTSGSVKSNKNLFESTPLYAMQDQSGH:171

*Tr* Xinβ46 IKELVEVGKLKKTVALVEERGDVRHQKWRFESQPLEEIREEKKDIIRTIDLEEIDKVTSKNFKHIFESAESS.RGDEP.....QKIQIEG...VTSGSVKSNKNLFESTTLYAMQDHTGH:925

*Ga* Xinβ16 LKELSEVGKLKKAVALNEEKGDVRHQKWRFESQPLEQIRQEKKEVLRTIDLEEIDRVDVSNYKQIFESTDSN.WRDES.....QKILIEG...ATSGSVKANKNLFESTSLYAMQDSSGH:725

*Ol* Xinβ21 LKELEEVGKLKKTVALQEEKGDVTHQKWRFESQPLEQIRDERKEVLRTIDLEEIDKVDVSNYKQIFESAELKEQREDL.....QKILIEG...VTSGSVKSNRDLFESSALYAMQDSSGH:727

*Dr* XinβNA IKELAEVGKLKRMMTS.EEKGDVRHQKWLFESQPLEQIREERKEITKTVNLEDIENVDVRNYKQIFETYDLS.QYDES.....QRIQVEG...VTTGSVRSNKDLFESMPLYALQDSSGH:637

*Tn* Xinβ3 QKELLEVGKLKKMVASEEEKGDVRHQKWVFESQPLENIREEKKEIVRTVNVEALDKGDVTNYKERFESMDLS.KCEGA.....QRIQVEG...VMSGSVKSNKELFESTTMYAMQDSFGR:483

*Tr* Xinβ36 LKDLLEVGKLKKMVTSEEEKGDVRHQKWVFESQPLENIREEKKEIIRTVNVEALDKGDVTNYKERFESMDLS.KCEGT.....QRIHVEG...VTSGSVKSNRELFESTPMYAMQDSSGH:1015

*Ga* Xinβ1 LKELLEVGKLQKMVASEEEKGDVRHQKWVFESEPLENIREEKKEITRTVNLEAVDKIDVTNYKERFESMDLS.KCEGT.....QKIQVEG...VTSGSVRSNRVLFESTPMYAMQDSSGH:773

*Ol* Xinβ2 LKELLQVGKLTKTVASEEEKGDVRHQKWVFESQPLESIREERKEITRTVNIQAQDKGDVTNYKERFETMDLS.KCERT.....EKIQVEG...VTSGSVKSNKDLFESTPLYAIQDSSGY:740

*Dr* Xinβ6 LKELPEVGKLQKMVASEEEKGDVRHQKWVFESKPLEQIREESKEITRTVNLEDLEKGDVTNYKERFETMDLS.KCLDA.....QKIHIEG...VTSGSVKSNKVFFESKPLYAVQDSSGH:1068

*Dr* Xinβ19 LKELPEVGKLQKMVASEEEKGDVRHQKWVFESKPLEQIREESKEITRTVNLEDLEKGDVTNYKERFETMDLS.KCLDA.....QKIHIEG...FTSGSVKSNKVFFESKPLYAVQDSSGH:810

*Pm* Xin FGTLMEVGKLKTVEASADERGDVRHAKWVFESKPLDLIQEEQKEIVKTVQRSGSERGEVKSVKEVFETVPLGSIQDTE.....KITESAG...IIGGDVKTTKSLFESVPLYAIRDSSGY:605

**XR17**

**XR18**

**XR16**

*Hs* Xinα LHALTSVSREQIVG.GDVQGYRWMFETQPLDQLGRS.PSTIDVVRGITRQEVVAGDVGTARWLFETQPLEMIHQREQQERQKEEGKSQGDPQPEAPPKGDVQTIRWLFETCPMSELAEKQ:610

*Pt* Xinα LHALTSVSREQIVG.GDVQGYRWMFETQPLDQLGRS.PSTIDVVRGITRQEVVAGDVGTARWLFETQPLEMIHQREQQERQKEEGKSQGDPQPEAPPKGDVQTIRWLFETCPMSELAEKQ:610

*Mam* Xinα LHALTSVSREQIVG.GDVQGYRWMFETQPLAQLGRS.PSTIDVVRGITRQEVVAGDVGTARWLFETQPLEMIHQREQQERQKEEGKSQGDPQPAAPSKGDVQTIRWLFETCPMSELAEKQ:610

*Cf* Xinα LHALTSVSREQIVG.GDVQGYRWMFETQPLDQLGRS.PSTVDVVRGITRQEVVAGDVGTARWLFETQPLEVIHQRERQGRQEEEGKSQAGSQPEALPKGDVQTIRWLFETCPMSELAEKQ:614

*Ec* Xinα LHALTSVSREQVVG.GDVKGYRWMFETQPLDQLGRN.PSTVDVVRGITRQEVVAGDVGTARWLFETQPLEVIHQREQQERQEEEGKSQAGPEPDAPLKGDVQTIRWLFETCPMSELAEKQ:614

*Bt* Xinα LHALTSVSREQVVG.GDVQGYKWMFETQPLDRLGRS.PSTVDVVRGITQQEVVAGDVGTTRWLFETQPLEVIHQREQQEREEEEGKPQGGPQPEIPHKGDVQTIRWLFETCPMSELAERQ:614

*Mm* Xinα LHALTSVSREQVVG.GDVQGYKWMFETQPLDTLGRS.PSTIDVVRGITRQEVVAGDVGTTRWLFETQPLEMIHQQEQQKPEEEEGKGPGGPPPELPKKGDVQTIRWLFETYPMSELAEKR:614

*Rn* Xinα LHALTSVSREQVVG.GDVQGYKWMFETQPLDRLGRS.PSTVDVVRGITRQEVVAGDVGTTRWLFETQPLEMIHQQEQQKREEEEGRGPGGPPPELPQKGDVQTIRWLFETYPMSELAEKQ:613

*Md* Xinα LHALTSVSREQVVG.GDVQGSRWMFETQPLDQLGQD.PGKVDVIRGITRQEVMAGDVSSAHWLFETQPLEVIHRQKKEPEEKE.......PPEAAPPKCNVQTVRWLFETQPIHALAQEP:607

*Gg* Xin FHEVTSVSREQVIS.GDVKKYKWMFETRPLDQFDES.TKKVDIIRGITKQEVVAGDVRTAKWLFETQPMDVIHHQATQGEE..HPSM....KREISQRGDVKTCRWLFETQPMHTLYEKA:645

*Ac* Xinα FHKVTSVSREEEVTGGDVKNYKWMFETIPLDQFDDS.TQKVALVKGITKQEVVAGDVKTAKWLFETQPIDVIHSQMNPADQ..DISE....KGEATQKGDVKTCRWLFETHPMDTLYEKE:651

*Xt* Xinα FHEVTSISREQVES.ADVRNYKWMFETKPLDQFNES.PQKVDIIRGITKEEIVSGDVGTAKWLFETQPVDIVHQQSDATEK..HSSV....QKQVLQKGDVKKCKWLFETQPIDKLYDKS:543

*Tn* Xinα15 LHRVTSARREEFVK.GKVQNYRWVFETKTLDELTEG.KGSVEMIKGITRQEQEIGDVKMVKWLFETQTIDGVQSRLKQRD......D....GASGGKRGDVQTCKWFFETEPMNILHDET:617

*Tr* Xinα296 LHRVTTVSREEFIK.EKFQYYRWMFETRPLDELTEG.KDSVEIIKGITRQEQETGDVRMAKWLFETQTIAGIQAKFNQRD......D....NASEVEKGDVKTRKWLFETKPMKILHDKT:617

*Ga* Xinα3 LHTVTTVSREELIK.GKVQNYKWMFETKPLDELAER.KANVEVIKGITREEDTMGDVKMAKWHFETQTIDGIHSKFNQTER.....D....ASVEHRKGDVNNCKWLFETQPMNIVDEKS:626

*Ol* Xinα17 LHEVTTVSREEFVK.GKVKNYKWMFETKPLDELADR.NGNVEVIKGITRQEECTGDLNMAKWLFETQTIDGIHCKVNQTEK..TS.S....DREELCKGDVKTCKWLFETQSMDVQCEKD:621

*Dr* Xinα2 FHEVTTVSREECIK.GNVQNYKWMFETRPLDQFEEG.SGKVELIKGITRQEDMTDDTRTAKWMFETQPLDCMSLNSRTDVD.....S....TQKEFKKSNVKTCKWLFETKPMDMLYEKS:626

*Hs* Xinβ YHQVKTVQQEEIVR.GDVRSCRWLFETRPIDQFDES.IHKFQIIRGISAQEIQTGNVKSAKWLFETQPLDSIKYFSDVEETESKTE.....QTRDIVKGDVKTCKWLFETQPMESLYEKV:1110

*Pt* Xinβ YHQVKTVQQEEIVR.GDVRSCRWLFETRPIDQFDES.IHKFQIIRGISAQEIQTGNVKSAKWLFETQPLDSIKYFSDVEETESKTE.....QTRDIVKGDVKTCKWLFETQPMESLYEKV:1041

*Mam* Xinβ YHQVKTVQQEEIIR.GDVRSCRWLFETRPIDQFDES.IHKFQIIRGISAQEIQTGNVKSAKWLFETQPLDSIKYFSDVEETESKTE.....QARDIIKGDVKTCKWLFETQPMESLYEKV:1116

*Cf* Xinβ YHQVKTVQQEEILR.GDVRSCRWLFETRPIDQFDES.IHKFQIIRGISAQEIQTGNVKSAKWLFETQPLDSIKYFSNMEEVESKTE.....QVTDIVKGDVKTCRWLFETQPMESLYEKV:928

*Ec* Xinβ YHQVKTVQQEEILR.GDVRSCRWLFETRPIDQFDES.IHKFQIIRGISAREIQTGNVKSAKWLFETQPLDSIKYFSNMEEVESKTE.....QATDIVKGDVKTCRWLFETQPMESLYEKV:882

*Bt* Xinβ YHQVKTVQQEEILR.GDVRSCRWLFETRPIDQFDES.IHKYQIIRGISAQEIQTGNVKSAKWLFETQPLDSIKHFSNMEEVERKTE.....QVTDIVKGDVKTCRWLFETQPMESLYEKV:930

*Mm* Xinβ YHQVKTVQQEEIVR.GDVRSCRWLFETRPIDQFDES.LHKFQIIRGISAQEIQAGNVKSARWLFETQPLDSIKYFSNVEETDSKTE.....QSTDIVKGDVKTCKWLFETQPMESLYEKA:882

*Rn* Xinβ YHQVKTVQQEEIVR.GDVRSCRWLFETRPIDQFDES.LHKFQIIRGISAQEIQAGNVKSARWLFETQPLDSIKYFSNVEETDSKTE.....QSTDIVKGDVKTCKWLFETQPMESLYEKA:880

*Md* Xinβ YHEVKTVQQEEILR.GDVRSCRWLFETRPIDQFDES.IHKYQIIKGISSREIQSGDVKSAKWLFETQPLDSIKHFNSMEDSESTKQ.....QTMDVVKGDVKAYTWLFETQPMETLYDKT:929

*Ac* Xinβ YHKVKTIRQEEIIR.GDVRNCRWLFETRPIDQFDES.IEKIEIIKGISSQEVQSGDVKTAKWLFETQPLDSIKYFAQMEDEESTEES....QATEVVKGDVKTCRWLFETQPMETLYDKE:791

*Xt* Xinβ FHEVQTIRKEDIVR.GDVQSCQWMFETIPIDQFRERSVENYQIIKGISSEQIQSGDVKTGKWLFETQPLDSIKYFSNAEDEEIITE.....NQTDIVKGDVKMCKWLFETQQMEDLYEKQ:872

*Tn* Xinβ2 FHEVRTVRREEVVK.GDVTTCKWMFETRPIDQFNDS.IDKYHVIKGISKQQIESGDVKTAKWLFETQPLDSIKYFSNIEDEEVVET.....NNIDILKGDVKTCKWLFETKPMDILYQKV:284

*Tr* Xinβ46 FHEVKTVRREEVVK.GDVTTCKWMFETRPIDQFHEC.IDKYQVIKGISKHQIESGDVKTAKWLFETQPLDSIKYFSNIEDDEAVGTS....NNIDIVKGDVKTCKWLFETKPMDILYEKV:1039

*Ga* Xinβ16 FHQVKTVRREEVVK.GDVTTCKWMFETRPIDQFDGS.IDRYQVIQGISKQEIESGDVKTAKWLFETQPLDAIKYFSNIEDEEVTGAK....INLDQVKGDVKTCKWLFETKPMDVLYERA:839

*Ol* Xinβ21 FHEVKTVRREEVVK.GDVTTYKWMFETQPIDQFDES.IEKYQIIKGISKQEIESGDVKTAKWLFETQPLDAIKYFSNIEDEETVGGI....KSLDVVKGDVKNCRWLFETKPMDALYERV:841

*Dr* XinβNA YHKVRTVRREEIVK.GDVRSCQWMFETRPIDQFDES.ISKFQIIKGITQQEVESGDVKTAKWLFETQPLDAIKYFSNIEDDECVTK.....ETADIVKGDVKTCKWLFETKPIDTLYERK:750

*Tn* Xinβ3 YHELRTVRREEIVK.GDVHSCRWMFETRPIDEFDES.INKFQIIKGISKQEIQSGDVKTARWLFETQQLDAIKYFNNFEDEEHKIK.....EGGEIEKGDVNTCRWLFETHPLDVLYEKV:596

*Tr* Xinβ36 YHELKTVRREEIVK.GDVHSCRWMFETRPIDEFDES.IDKFQIIKGISKQEVQSGDVKTAKWLFETQQLDAIKYFNNFEDEEHEIK.....EGTEIEKGDVKTCRWLFETRPLDGLYDKV:1128

*Ga* Xinβ1 YHEVKTVRREEIVK.GDVRSCRWMFETRPIDEFDES.INRFQIIKGISKQEVESGDVKTAKWLFETQALDSIKSFGQCEDEEHACK.....EGIEIEKGDVKTCRWLFETQPMDALYEKM:886

*Ol* Xinβ2 YHEVRTVRREEIVK.GDIHSCRWMFETRPIDQFDES.ISKFQIIKGISKQEIESGDVKTAKWLFETQPLDAIKYFSNAEVKEQKTK.....E..EIEKGDVKTCRWLFETQPMDVLYEKV:851

*Dr* Xinβ6 YHEVKTVRREEVVK.GDVRSCKWMFETRPIDQFDES.ITKFQIIKGISKEEIQSGDVKTAKWLFETQPLDGIKYFN.LEEEDNRKN.....ESIEIQRGDVKTCRWLFETQPMDVLYEKI:1180

*Dr* Xinβ19 YHEVKTVRREEVVK.GDVRSCKWMFETRPIDQFDES.ITKFQIIKGISKEEIQSGDVKTAKWLFETQPLDGIKYFN.LEEEDNRKN.....ESIEIQRGDVKTCRWLFETQPMDVLYEKI:922

*Pm* Xin YHQVQTVRREEVSS.GDVKSCRWMFETKPLDQFDEESLQKIQIIKGISKEELQAGDVKTAKWLFETHPLDTIKNAD....EESVTQ.....QRTEVHRGDVKMCRWLFETRPMDALYEKV:715

**XR20**

**XR19**

**XR21**

*Hs* Xinα GSEVTD...PTAKAEAQSCTWMFKPQPVDRPVG.....SREQHLQVS..QVPAGERQTDRHVFETEPLQASGRP..CGRRP.VRYCSRVEIPSGQVSRQKEVFQA..LEAGKKEEQEPR.:714

*Pt* Xinα GSEVTD...PTAKAEAQSCTWMFKPQPVDRPVG.....SREQHLQVS..QVPAGERQTDRHVFETEPLQASGRP..CGRGP.VRYCSRVEIPSGQVSRQKEVFQA..LEAGKKEEQEPR.:714

*Mam* Xinα GSEVTD...PTAKAEAQSCTWMFKPQPVDRPVG.....SREQHLQVS..QVPAGERQTDRHVFETEPLQASGRP..CGRGP.VRYCSRVEIPSGQVSRQKEVFQA..LEAGKKEEQQPR.:714

*Cf* Xinα GPEVTE...PTAKAKPRSCTWMFAPQPLERPEG.....SREQHLQVS..QIQAGERQTDRHVFETEPLQASGRP..CGRGP.VRYCSRVEIPSGQVSRQKEVFQA..LEAGKREEQGPR.:718

*Ec* Xinα GSEVTD...PTTKAKERSCTWMFTPQALDRPEG.....SREKHLQVS..QVQDGERQTDRHVFETEPLQTSGRP..CGKGP.VRYCSRVEIPSGQVSRQNEVFQA..LEAGKREDQGSR.:718

*Bt* Xinα GSEVTD...LTSKAR..SCTWMFAPQSPDWPEG.....SKEQHLEVS..QVQAGERQTERHVFETEPLQAAGHP..CGRGP.VRYCSRVDIPSGQVSRQKEVFQA..LEAGKREDQGPR.:718

*Mm* Xinα ESEVTD...PVSKAETQSCTWMFGPQSLNPAEG.....SGEQHLQTS..QVPAGDRQTDRHVFETESLPASNQS..SGRKP.VRYCSRVEIPSGQVSRQKEVFQA..LEAGKKEVPETT.:718

*Rn* Xinα ESEVTG...PVSKAEAQSCTWMFGSQSLNQAEG.....SGEQHLQTS..QVQAGDRQTDSHVFETESLSAQGQS..SGRKP.VRYCSRVEIPSGQVSRQKEVFQA..LEAGKRGVQETV.:717

*Md* Xinα RPEVPG...TSPAADVRSCTWMFDMQPPEGPES.....PREQHLQVRPTSLPSRDPQMEAHVFETEPLLPAPGA..GGKDP.VHYCSRVEISVGQVSRRKEAFEAPAVAGGQKGQQGP..:714

*Gg* Xin EKKQEE.DVSVPQADVKSYTWMFETQPLDSLKG.QEEQYLRVSKAYSQDELQGVDVKTVRHLFETEPLGSSVVSEADQKKT.LRYSSRVEIQSGEVSRVKEFFEAKPLDTTTKPTAVIKD:762

*Ac* Xinα GEKQDG.EECVPQGDVKSCTWMFETQPLDSLKG.QEEQYLQVSKVYNQDELQGVDVKTVRHLFETEPLVSDTTNSGDTKKI.IRYSSRVEMQSGEVSRVKEFFEAKPLDSMHKPSIDKKA:768

*Xt* Xinα EQTKDT.EAQV.QGDVKSYTWMFESQPLDSFKD.SEEQYINV.KTTLQDACKGVDVKTSKHLFETEPIEN.ISSKGEFRKI.IRYSSTVQMPSGEVSRVKEIFEATPDKGIGGKGADIPV:657

*Tn* Xinα15 EKRNDN.QATK.GAGDRTTTWLFESQPLDSVRD.GDERRLKLCDTIQGSVKPEVDLQPAKHLFETETLDR.VRKDPSSHSE.VRLVSNVNFQSGDVSRVRELFESQSLDEIGSEMVTTSL:732

*Tr* Xinα296 EKPNDN.EPPK.GADVKSVTWLFESQPFDSIRD.GEEHSLKLCNTIEGSVTSEVDFQTVKHLFETESLDR.IRKDANSERD.VRLVSRVNFQSGDVSRVRELFESQSLDEIGSEMETPSS:732

*Ga* Xinα3 EKMNDK.EATD.NTNVKSITWLFESQPLDSIKD.GEEYNLKLCNTIEDSVKSEVGVQTVKHVFETETVNR.IRKDANPKHD.VRRVSQVNFQSGDVSRVKELFESHSLDEIGSEMGKCD.:740

*Ol* Xinα17 ERLKDQ.DLTD.GTNVRSITWLFESQPLDSIKQ.GEPYSLRLCSTVQDSVRSQVDVQTVKHLFETETLDR.IRKDSDSEQD.LRCISQVNFQSGDVSRVKELFESQSLNEIGSEIVKAT.:735

*Dr* Xinα2 EGKQDV.EPVP.KADVKSHTWLFETQPLDNIKD.KENLGLKLCSTVQEDVKSDVNVKTVKHLFETEPLDR.ITDQADSGQN.VRCVSQVDMQSGDVSRVKEIFESKSL..G.TESSKWS.:737

*Hs* Xinβ SLMTSS..EEIHKGDVKTCTWLFETQPLDTIKDDSET..AVKLQTVKQEEIQGGDVRTACFLFETENLDS.IQG..EEVKE.IK.PVEMDIQAGDVSSMRYKFENQSLDSISSSSEEVLK:1221

*Pt* Xinβ SLMTSS..EEIHKGDVKTCTWLFETQPLDTIKDDSET..AVKLQTVKQEEIQGGDVRTACFLFETENLDS.IQG..EEVKE.IK.PVEMDIQAGDVSSMRYKFENQSLDSISSSSEEVLK:1152

*Mam* Xinβ SLMTSS..EEIHKGDVKTCTWLFETQPLDAIKDDSET..KVKLQTVKQEEIQGGDVRTTCFLFETENLDS.IQG..EEVKE.IK.PVEMDIQAGDVSSMRYKFENQSLDSISSSSEEVLK:1227

*Cf* Xinβ SLMTGS..EEIHKGDVKACTWLFETKPLDTIKDDSEA..TTKLQTVKQEEIQGGDVRTACFLFETENLDS.IQG..EEGKE.IK.AVEMDIQAGDVSSMRHKFENQPLDSISSSSEEVLK:1039

*Ec* Xinβ SLMTGS..EEIHKGDVKACTWLFETQPLDTIKDDSEA..TVKLQTVKQEEIQGGDVRTACFLFETENLDS.IQG..EEGKE.IK.AVEMDIQAGDVSSMRHKFESQPLDSISSSSEEVLK:993

*Bt* Xinβ SLMTGS..KEIHKGDVKACTWLFETQPLDTIKDDSEA..TVKLQTVKQEEIHGGDVRTTCFLFETENLDS.IQG..EEGKE.IK.AIEMDIQAGDVSSMRHKFESQSLDSISSGSEEVLR:1041

*Mm* Xinβ SLMTNS..EDIHKGDVRTCMWLFETQPLDAIKDDSEA..TVKLQTVKQEEIQGGDVQTACFLFETENLDN.IQG..DEGKE.NK.PLEMDIQSGDVSGMKFKFENQSLDSINCSSENVLS:993

*Rn* Xinβ SLMTNS..EDIHKGDVRTCMWLFETQPLDAIKNDSEA..TVKLQTVKQEEIQGGDVRTACLLFETENLDN.IQG..GEGKE.TK.PVEMDIESGDVSGMKYKFENQSLDSISCSSENVLN:991

*Md* Xinβ ELITNN..EEIQKGDVKTCTWLFETQPLDEIRDDSEA..IVKLQTVKQEDIQGRDVRTACFLFETENLDN.IQG..EEGKE.IK.SVEVDIQSGDVSGMKYKFENQSLDSISSSSEEVLK:1040

*Ac* Xinβ TIESDS..EEIHKGDVKTCTWLFETRPLDAMGEDSET..AIKLHTVEQAEIQGSDVRTACFLFETEKLEN.IQG..EESKE.IKRVVEIDIQPGDVSTMKYKFENQSLDSINSSSEEVLK:903

*Xt* Xinβ DKISAD..VDIQRGDVKTCTWLFETQPLDSIHDESE.....KTETTNQKNIQGRDVQRVCFLFETENLDN.IQE..EGKRD.FKRVVEIDIQSGDVSSMKYIFENQPLDKISSSSEEVLQ:981

*Tn* Xinβ2 ELEGEG.STEVQKGDVKTCTWLFETKALDTLHD..ETETVLKTCTVNQEDIKGKDVKTACFLFETEN....VSQ..EESET.FKRVTEIDIASGDVSRMKYIFENQTGNIMTSTSEELSQ:394

*Tr* Xinβ46 KLEDEG.SEEIQKGDVKTCTWLFETQALDTLHD..ETETVLKTCTVNQEDIRGKDVKTACFLFETEN....VSQ..EETGS.FKRVTEIDIASGDVSRMKYIFENQTGDIMTSTSEEVLQ:1149

*Ga* Xinβ16 ELKGENESEEMQKGDVKTSTWLFETQALDTIRD..ET..ILKTCTVNQEDIKGKDVRTACFLFETEKLED.LSR..EETGS.FKRVTEIDVASGDVSGKKYIFENQTSDIMSSTTEELMQ:951

*Ol* Xinβ21 EKESIDETEEVRKGDVKTCTWLFETHTLDTIHD..ETETVLKTCSVQQEDIRGKDVKTARFLFETEKLED.LAG..GETGS.YKRITEIDIASGDVSRMKYIFENQMSDILTSTSKEVMQ:955

*Dr* XinβNA ELESTKDSDEVQKGDVKTCTWLFESQTLDAIQD..NSEKVLQIRTVNQEDIHGKDVRMARFLFETENLEN.IAG..EDREA.FKSVTEIDIQSGDVSRMKYIFETQSSDVVSSTSEDFMR:864

*Tn* Xinβ3 EKNEAD.IKEVEKGDVKTCTWLFETQTLDTIRDHTEAETILKTCTVKEEDIQGKDVRLARFLFETENLEN.ITG..DDSSS.FRKVTEINIQSGDVSRMKYIFENRSSDIMSSTSEETMQ:711

*Tr* Xinβ36 EKNEAD.IEEVAKGDVKTCTWLFETQTLDNIRDHTESETILKTCTVKEEDIQGKDVRLARFLFETENLEN.ITG..EDSSS.FRKVTEINIQSGDVSRMKYIFENRSSDIMSSTSEETMQ:1243

*Ga* Xinβ1 EKSEVD.VEEVQRGDVKTCTWLFETQTLDNIRDHTESETILKTCTVKQDDVQGKDVRLARFLFETENLEN.LTG..EDGGS.FRRVTEIDVQSGDVSRMKYIFENRSSDIMSSSSGETMQ:1001

*Ol* Xinβ2 DRSETD.VKEVHKGDVKTCTWLFETQMLDNIRDHSDMENILKTCTVKQQDIQGKDVGLARILFETENLEN.ITG..EESGS.FRRVTEIDIQSGDVSRMKCIFENRSSDIMSSTSEETMQ:966

*Dr* Xinβ6 ETKTED.TTDIQKGDVKTCTWLFETQTLDSIKD..ESETILKTSTVKQDDVQGKDVRLVRFLFETENLEN.ITD..DEDHSGFKRITEIDINSGDVSRMKYIFENQTSDIMTSTSEETMQ:1294

*Dr* Xinβ19 ETKTED.TTDIQKGDVKTCTWLFETQTLDSIKD..ESETILKTSTVKQDDVQGKDVRLVRFLFETENLEN.ITD..DEDHSGFKRITEIDINSGDVSRMKYIFENQTSDIMTSTSEETMQ:1036

*Pm* Xin EKREGE..EEVMKGDVKTCTWLFENQPLDTLVADGAAH.KLQVRSLRQEDVEGSDVRTRRILFETERLDS.IRGGFEEGQD.VRRITNIDVQSGDVSRVKWRFENHPLDTIVG..ESTGQ:828

**XR23**

**XR22**

**XR24**

*Hs* Xinα ...VI.AGSIPA....................................GSVHKFTWLFENCPMG........................................................:738

*Pt* Xinα ...VI.AGSIPA....................................GSVHKFTWLFENCPMG........................................................:738

*Mam* Xinα ...VI.AGSIPA....................................GSVHKFTWLFENCPMG........................................................:738

*Cf* Xinα ...VL.PEPISV....................................GSVHKFTWLFENCPMG........................................................:742

*Ec* Xinα ...VI.PEPIPE....................................GSVHKFTWLFENCPMG........................................................:742

*Bt* Xinα ...EI.PEPISA....................................GSVHKFTWLFENCPMG........................................................:740

*Mm* Xinα ...IN.LGSIPT....................................GSVHKFTWLFENCPMG........................................................:742

*Rn* Xinα ...LS.PGSIPT....................................GSVHKFTWLFENCPMG........................................................:741

*Md* Xinα ......PEPVPE....................................GSVRKFTWLFENCPMD........................................................:736

*Gg* Xin ......DGTIEA....................................GSVHKFTWLFENYPMDTLKD..SSEGIQEIPPEKDIKGGDVGGKRFIFETYSLDQIHDK....VDETELHKI:834

*Ac* Xinα SETPA.HGDIER....................................GAVHKFTWLFENCPMDSLKS..SMEGIQEVAPQKDIQGGDVGGKRFVFETYSLDQIHDK....ENEMQIKKI:845

*Xt* Xinα ......EENIQK....................................GSVNKFTWLFENRPLDSMSA..STNGFQEVPPEKDVQGGDVVGKRFVFETYSLDQIKQE....ADETEIKKV:729

*Tn* Xinα15 DEQNR.GEQLEK....................................GSVHKFTWMFENCPMNLINK..D.KTDEDVQEICVIESGDVQNKRFIFETSSLDRIHDK....LLDKKFITV:808

*Tr* Xinα296 DEQNG.EERVEK....................................GSVHKFTWMFENCPMNLINK..D.KNEGDIQEICVIESGDVQSKKFIFETSSLDSIHDR....PLDEKLITV:808

*Ga* Xinα3 QQQNQ.EEELIK....................................GAVHKCTWMFENCPMNQMNK..D.IEGANIQRVSGEEIGDVQNKKFVFETSSLDKIQDG....PLEQTSDPV:816

*Ol* Xinα17 ESQSQ.DEEIEK....................................GSVHKVTWMFENCPMNLINK..D.SDKSNTQRLS.QESGDVQNKKFIFETSSLDKIQKE....PLKQVSASV:810

*Dr* Xinα2 ..EEQ.KNEIQS....................................GSVHKFTWLFENQPIGDIN......EKEERIVSCDVEAGDVGGKKFIFETLSLDKIKDKDE..LLEHPSMII:810

*Hs* Xinβ KIKTLKTEDIQK....................................GNVLNCRWLFENQPIDKIK..ESQEGDECVKTVTDIQGGDVRKGCFIFETFSLDEIKEES...DYISTKKTI:1300

*Pt* Xinβ KIKTLKTEDIQK....................................GNVLNCRWLFENQPIDKIK..ESQEGDECVKTVTDIQGGDVRKGCFIFETFSLDEIKEES...DYISTKKTI:1231

*Mam* Xinβ KIKTLKTEDIQK....................................GNVLNCRWLFENQPIDKIK..ESQEGDECVKTVTDIQGGDVRKGCFIFETFSLDEIKEES...DYISTKKTI:1306

*Cf* Xinβ KIKALKTEDIQK....................................GNVLNCRWLFENQPIDMIK..ENHEGDELVKTVTDVQGGDVRKGCFIFETFSLDEIKEES...DCISTKKTI:1118

*Ec* Xinβ KIKTLKTEDIQK....................................GNVLNCRWLFENQPIDMIK..ERQGGDELVKTVTDIQGGDVGKGCFIFETFSLDEIKEES...DYISTKKIT:1072

*Bt* Xinβ KIKTLKAEDIQK....................................GNVLNCRWLFENQPIDMIK..ESQEGDELVKTVTDIQGGNVRKGCFIFETFSLDEIKEES...DYISTKKTI:1120

*Mm* Xinβ KIKTLKAEDIQK....................................GNVLKCRWLFENQPIDMIK..ESQECDGLVKTVTDVQGGDVRKGCFIFETFSLDEIKDES...DGISMRETN:1072

*Rn* Xinβ KIKTLKIEDIQK....................................GNVLNCRWLFENQPIDMIK..ENQEGDGLVKTVTDIQGGDVRKGCFIFETFSLDEIKDES...DVISTRQTN:1070

*Md* Xinβ KIKTLQAEDIQR....................................GDVLNCRWLFENQPIDMIK..EIQEGDESLKTVTDIQGGNVRKGCFIFETFSLDQIKDKS...EEISIEKTF:1119

*Ac* Xinβ KIKTLQYEDIQK....................................GDVLRCCWLFENQSLDEIA..EHEEDKTSVKTVTDIQGGNVRKGCFIFETFSLDQIKEESS..EDISAIKTV:983

*Xt* Xinβ KIKTMNQEDLQN....................................GNVLSCKWLFENHFIDEIN..EDHVKNKSEFAISDVQGGNVRKGCFIFETFSLDQITEKN...SEESFTRTI:1060

*Tn* Xinβ2 KLKKVWTEDAHK....................................GDVVNCKWLFENQHNDGP......EDSMCNRTVNDVQGGDVDKGRFIFETRRML..................:454

*Tr* Xinβ46 KLKNVWTEDAHK....................................GDVVNCKWLFENRHVDGP......EESTCNRTVNDVQGGDVDKGRFIFETYSLDEIKSSSETDEELIKMQKI:1227

*Ga* Xinβ16 NLKRVQSEDIQR....................................GNVVNCKWLFENQSMDTI..HNSHEEYTSSRTVNDVQGGDVDKGRFIFETYSLDEIQETD...KELMKMRKI:1030

*Ol* Xinβ21 NLKRVQTEDIQR....................................GNVVNCKWLFENQSIDAIGLQDNEEESVSSRTVNNIQGGDVNKSRFLFETRSLDAIQNVS...SDIMKVHKI:1036

*Dr* XinβNA QLRSAQADDIQK....................................GNVGNCKWLFENQPIDEIS..ENPSELKGARMIQDVQGGNVDKGRFIFETYSLDKIQDSE...AEIGKLQKV:943

*Tn* Xinβ3 RLKLQQAEDIQR....................................GNVVNCTWMFENQPIDEIR..EEARDVR...TVTDVQGGNVDQGRFIFETYSLDQIKEES...SDMSKITSI:787

*Tr* Xinβ36 RLKLQQAEDIQR....................................GNVVNCTWMFENQPMDEIR..EEARDVR...TVTDVQGGDVDKGRFIFETYSLDQIKEES...SDMSKLTSI:1319

*Ga* Xinβ1 RLKTQQVEEIQK....................................GDVVNCTWMFEHHPIDNIR..DESADAKESRTVTDVKGGDVDKGRFIFETYSLDEIKEDST.ETDKSKITSI:1082

*Ol* Xinβ2 RLKTQQAEDIQK....................................GNVVNCTWMFENKPIDAIC..DAKERETR..TVIDVQGGNVDKGRFIFETYSLDQIKDESS.EANISQLTGV:1045

*Dr* Xinβ6 KLKCHQTEDIQK....................................GNVVNCTWMFENQSIDSIK..ANSEDFKESRTVTDIQGGNVDKGRFIFETYSLDKIQEESS.ETEISKLQSI:1375

*Dr* Xinβ19 KLKCHQTEDIQK....................................GNVVNCTWMFENQSIDSIR..ANSEDFKESRTVTDIQGGNVDKGRFIFETYSLDKIQEESS.ETEISKLQSI:1117

*Pm* Xin KLKTVQAEDIERGDVNKYTSLFETQPIGAIGEDPDAVKLRRTVTDVQGGNVHMCSWLFENRPMGTIG..EDPEAAQLVGTISDVVGGDVKSGRVVFETRSLDQIRGDG...SDVS...DA:940

**XR25**

**XR26**

**XR27**

*Hs* Xinα ........................................................................................................................:738

*Pt* Xinα ........................................................................................................................:738

*Mam* Xinα ........................................................................................................................:738

*Cf* Xinα ........................................................................................................................:742

*Ec* Xinα ........................................................................................................................:742

*Bt* Xinα ........................................................................................................................:740

*Mm* Xinα ........................................................................................................................:742

*Rn* Xinα ........................................................................................................................:741

*Md* Xinα ........................................................................................................................:736

*Gg* Xin QKDTMSKANVKSCTMLFESQPLYAIQDKEGGYHEVTSVQKEEIMKGDVKGARWLFETKPLDQIKKEEEVFVIRAVTQEDIKKGDVQAARWRFETEPLDSFPGGKISVPRTVDDVQK.GDV:953

*Ac* Xinα QEETMNKADIKSCTMLFETRPLYAIQDKEGEYHEVTSVKKEEIMKGDVKGARWQFETKPLDQIKKDEEVFVIRAVTQEDFKKGDVQAARWRFETEPLDSIAEEKRSVLKTIDDVQK.GDV:964

*Xt* Xinα QES.VTRGDVKSCMMLFETNPLYAIQDKGGEYHEVTSVKKEEVLKGDVRGAKWLFETKPLDKINKDEEVFVIRAVTQEDIDKGSVTAARWRFETEPLDSVTEKNKYTGKELEDVQK.GDV:847

*Tn* Xinα15 EEPVSN.VDVKSSTMMFESLPLYAIRDKEGQFHEVTTVKKEEVLSGDVREARWMFETKPLDAIKAENEVYVIRAVTQEDIKKGDVQSARWKFETQPLDSLTSRDEPTVRVVEDIGS.TNV:926

*Tr* Xinα296 EQPVSN.VDVKSSTMMFESLPLYAIRDKEGQFHEVTTVKKEEVLSHDVREARWMFETKPLDAIKAENEVYVIRAVTQEDIKKGDVQSARWKFETQPLDSLTSRDESPVRVMEDIGS.TSV:926

*Ga* Xinα3 EQLLTS.VDVKSSTMMFESLPLYAIRDKEGQFHEVTTVKKEEVMSGDVRGARWMFETKPLDAIKEDKEVYVIRAVTQEDVKKGDVKSARWKFETQPLDSLTSRDEPSVRVIEDLGS.RNV:934

*Ol* Xinα17 EEPVSN.VDVKSSTMMFESLPLYAIRDKEGQFHEVTTVKKEEVMSGDVRGARWMFETKPLDAIKAENEVYVIRAVTQEDVNKGGVESARWKFETQPLDSFGGQDEPSVSVSEDFGS.ISV:928

*Dr* Xinα2 EKPLSSSVNVKSNTMLFESQPLYAIRDKDGQFHEVTTVMKEEVMRGDVRGARWMFETKPLDTIQADKEIYVIRAVTQEDVHKGDVKSARWKFETQPLDSFTPHEGPSVRVVEDIGNEKCV:930

*Hs* Xinβ T.EEVIQGDVKSYRMLFETQPLYAIQDREGSYHEVTTVKKEEVIHGDVRGTRWLFETKPLDSINKSETVYVIKSVTQEDIQKGDVSSVRYRFETQPLDQISEESHNIMPSIDHIQG.GNV:1418

*Pt* Xinβ T.EEAIQGDVKSYRMLFETQPLYAIQDREGSYHEVTTVKKEEVIHGDVRGTRWLFETKPLDSINKSETVYVIKSVTQEDIQKGDVSSVRYRFETQPLDQISEESHNIMPSIDHIQG.GNV:1349

*Mam* Xinβ T.EEVMQGDVKSYRMLFETQPLYAIQDREGSYHEVTTVKKEEVIHGDVRGTRWLFETKPLDSINKSETVYVIKAVTQEDIQKGDVSSVRYKFETQPLDQISEESHNIVPTVDHIQG.GNV:1424

*Cf* Xinβ T.EEVMKGDVKSYRMLFETQPLYAIQDREGYYHEVTTVKKEEVIHGDVRGTRWLFETKPLDSINQSETVYVIKSVTQEDIQKGDVSSVRYRFETQPLDQISEESRDAMLTVDYIQG.GDV:1236

*Ec* Xinβ A.EEVIKGDVKSYRMLFETQPLYAIQDREGCYHEVTTVRKEEVIHGDVRGTRWLFETKPLDSINESENVYVIKSVTQEDIQKGDVSSVRYRFETQPLDQISEESRDIVPTVDYIQG.GNV:1190

*Bt* Xinβ T.EEVIKGDVKSYRMLFETQPLYAIQDREGFYHEVTTVKKEEVIHGDVRGTRWLFETKPLDSINESETVYVIKSVTQEDIQKGDVSSVRYRFETQPLDQIAKESRDIVPTVDCIQG.GDV:1238

*Mm* Xinβ L.GEIIKGDVKSYKMLFETQPLYAIQDHEGFYHEVTTVKKEETIHGDVRGTRWLFETKPLDSIHESEDVYVIKSVTQEDIQKGDVSSVRYRFETQPLDMISDKSHNIVPTVDYIQG.GNV:1190

*Rn* Xinβ T.EEVIKGDVKSYKMLFETQPLYAIQDQEGFYHEVTTVKKEETIHGDVRGTRWLFETKPLDSINASEDVYIIKSVTQEDIQKGDVSSVRYRFETQPLDMISDKSHNIMPTIDHIQG.GNV:1188

*Md* Xinβ SKDEIIKGDVKNYRMLFETQPLYAIQDKEGYYHEVTTVKKEEVIHGDVRGTRWLFETKPLDSINESENVYVIKSVTQEDIQKGDVSSVRYRFETQPLDTISKGAKVIVPTIDCIQG.GNV:1238

*Ac* Xinβ REDEITKGDVNSYRMLFETQPLYAIQDREGYYHEVTTVKKEEVIHGDVRGTRWLFETKPLGSINKSDNVYLIKSVTQEDIQKGDVSSVRYRFETQPLDTISDEEKIIVPTVDSVQG.GDV:1102

*Xt* Xinβ NEEEIIKGDVKNYTLMFETQPLYAIQDKEGFYHEVTTVKKEEVSHGNVRGTRWLFETKPLDSFQDSDKVYIIKAVTQEDIQKGDVNSVRYRFETQSLDTISEDTKSVFRTIEDVQG.GDV:1179

*Tn* Xinβ2 ICE.....................................................................................................................:457

*Tr* Xinβ46 ICEEEEKG.......................................................DDTDEVHIIKSVTQEDLQKGDVTSAKWKFETHPLDRITEEKKILIRTVDDIQG.GNV:1291

*Ga* Xinβ16 SRDEEERGDVRNYTMMFENQPLYAIQDKEGLYHEVTTVTSEEVTSGDVVGTRWKFETKPLDAIKDTDEVYIIKSVAQQDVHRGDVTSAKWKFETQPLDRITEGKETLVKTVNDIQG.GNV:1149

*Ol* Xinβ21 IRDEDERGDVRNYTMMFETQPLYAIQDKEGHYHEVTTVTSEEVKEGNVLGTRWLFETKPLDAIKDTDEVYVIKSVTQQDVQKGDVTSAKWKFETQPLDRIAEENKPLIKTVEDIKG.GNV:1155

*Dr* XinβNA IREDEEKGDVKTYAMMFETQPLYAIQDKEGHYHEVTTLTKEEIQRGDVVGARWLFETKPLDSIRETDEVYILKAVTQEDIHSGDVSSARWRFETQALDKIAEDAKISIKTVEDIQG.GDV:1062

*Tn* Xinβ3 FRDERERGDVKNYTMMFENQPLYAIRDKEGHYHEVTTVTKEEVMRGDVVGARWLFETKPLDSIRDSEEVYVIKSVTEEDINKGDVTSARWKFETQPLDEITDEIKVRSKTVADIQG.GDV:906

*Tr* Xinβ36 FRDEREKGDVKNYTMMFENQPLYAIRDKEGHYHEVTTVTKEEIMRGDVVGARWLFETKPLDSIRDSEEVYVINSVTEEGINKGDVSSARWKFETQPLDEITDEIKVRSKTVADIQG.GDV:1438

*Ga* Xinβ1 FREEVERGDVKNYTMMFETQPLYAICDKEGRYHEVTTVTKEEIIRGDVVGARWQFETKPLDSIRDSEEVYVIKAVTEEGINKGDVNTARWRFETQPLDEITEEGKVRLKSVADIQG.GDV:1201

*Ol* Xinβ2 FRNDMEKGDVKNYKMMFETQPLYAICDKEGHYHEVTTVTKEEIMRGDVVGARWLFETKPLDSIRDTEEVYVIKAVTEEGINKGDVNSARWRFETQPLDEITEEIKVKSKTVADIQG.GDV:1164

*Dr* Xinβ6 IHKEIEKGDVKNYTMMFETQPLYAIKDKEGHYHEVTTVTKEEILRGDVVGARWLFETKPIDSIKDTDEVYVIKAVTQEDIQKGDVSTARWRFETQPLDEIAEDMKVAVKTVADIQG.GDV:1494

*Dr* Xinβ19 IHKEIEKGDVKNYTMMFETQPLYAIKDKEGHYHEVTTVTKEEILRGDVVGARWLFETKPIDSIKDTDEVYVIKAVTQEDIQKGDVSTARWRFETQPLDEIAEDMKVAVKTVADIQG.GDV:1236

*Pm* Xin TKEEVIGGDVKSFTMLFETQPLYAIQDREGYFHEVVTVKKEEVMRADVSSARWLFETRPLDAIGAGQEVHVIRAVTQEDVQSGDVQTARWRFETQPLDTIAEDGKVLIRTVDDVQG.GNV:1059

**XR29**

**XR31**

**XR28**

**XR30**

*Hs* Xinα .......................................................................................................................:738

*Pt* Xinα .......................................................................................................................:738

*Mam* Xinα .......................................................................................................................:738

*Cf* Xinα .......................................................................................................................:742

*Ec* Xinα .......................................................................................................................:742

*Bt* Xinα .......................................................................................................................:740

*Mm* Xinα .......................................................................................................................:742

*Rn* Xinα .......................................................................................................................:741

*Md* Xinα .......................................................................................................................:736

*Gg* Xin QSNKQLFESQQVGQKKYVRMVSVSDVQRGDVRTSTWLFENQPVDSLYGDADRSSSISTVQREDSQKGDVKRCTWLFETQPMDTLKDPEVTVSTGT..QEPIPRADVKSTTWLFESTPLD:1070

*Ac* Xinα QSNKQLFESDQASQRKYVRMVSVSDVQHGDVRTSTWLFENQPIDSLKGESESTSCLSTVQREDIHKGDVKRCTWMFETQPMDSLKDPEAPANVET..LRVAPQADVKSTTWLFESTPLD:1081

*Xt* Xinα QLNKQLFESQQLNQKKYVRLVSVSDVQKGNVRTSTWLFENQPIDSLKGDSDEHPGIVTVQREDNQRGDVKRCTWLFESQPLDSLKDTDIPTAQG...QEEIPQANVKSTTWLFESTPLD:963

*Tn* Xinα15 QVNKEKFESDQ..ASRFVRMVSVTDVQHGDVRTSTWLFENQNIDSLKGDSQEQSPVKTVHREDSQKGDVRRCTWLFESQPLDKIKESEDAVVQGS..EEDIPKADVKCTTWLFETTPLD:1041

*Tr* Xinα296 QVNKEKFESNQ..CSKFVRMVSVTDVQHGDVRTSTWLFENQSIDSLKGDSQEESPVKTVHREDSQKGDVKRCTWLFESQPLDKIKESEDTLVQGS..EEAIPKADVKCTTWLFETTPLD:1041

*Ga* Xinα3 QLNKQIFESEQS.CQKFMRMVSVTDVQHGDVRTSTWLFENQTIDSLKGEPQEQGLVKTVHREDSQKGDVKRCTWLFESQPLDKIKDPEEVSTQRS..EDEYLSADVIWHYLGFFKPLHW:1050

*Ol* Xinα17 QENKKLFESEPS.NQKFVRTVSVTDVQRGDVRTSTWLFENQTIDHLKGEPEEQSPVKIVHREDSQKGDVKRCTWLFESNPLDKIKDSENTTVQG...VEEIPKTDVKCTTWLFETTPLD:1043

*Dr* Xinα2 QQSRQLFETEQASQKKFVRMVSVTDVQQGDVRTSTWLFENQPIDTLKGEPDEQNNLTAVHREDNTKGDVKRCTWLFESQSLDKIKDNKPTEELVSS.REEIPKADVKSTTWLFETTPLD:1048

*Hs* Xinβ KTSRQFFESENFDKNNYIRTVSVNEIQKGNVKTSTWLFETHTMDELRGEGLEYENIKTVTQEDVQKGDVKQAVWLFENRTFDSIMEAHKGITKMTK..EEIPPSDVKTTTWLFETTPLH:1535

*Pt* Xinβ KTSRQFFESENFDKNNYIRTVSVNEIQKGNVKTSTWLFETHTMDELRGEGLEYENIKTVTQEDVQKGDVKQAVWLFENRTFDSIMEAHKGITKMTK..EEIPPSDVKTTTWLFETTPLH:1466

*Mam* Xinβ KTSKQFFESENFDKNNYIRTVSVNEIQKGNVKTSTWLFETHTIDELRGEGLEYENIKTVTQEDVQKGDVKQAVWLFENQTFDSIMEAHKGVTKVTK..EEIPPSDVKTTTWLFETTPLH:1541

*Cf* Xinβ KAGRQFFESENSGKNTYVRTVSVSEIQKGNVKTSTWLFETHTIDELRGEGSEYENIKTVTQEDMQKGDVKQAVWLFENQTLDSIREADESTTKVIK..EEIPPSDVKTTTWLFETTPLH:1353

*Ec* Xinβ KTSKQFFESENFGKNTYIRTVSVNEIQKGNVKTSTWLFETHTIDELRGEGSEYENIKTVTREDMQKGDVKQAVWLFENQSLDSIKGADESITKLTK..EEIPPSDVKTTTWLFETTPLH:1307

*Bt* Xinβ RKSKQFFESENLDKNTYVRTVSVNEIQKGNVKTSTWLFETHTLDELRGEGSEYENIKTVTQEDMQKGDVKQAVWLFENQTLDSIKEADESITKITK..EEIPPADVKTTTWLFETTPLH:1355

*Mm* Xinβ QMNKQLFESEGGNKKNYVRTVSVNEIQKGNVKTSTWLFETHRIDEL.GEESRYENIKTVTQEDVQKGDVKQAVWLFENQTLDSINELDENDTKMTK..EEIPPSDVKTTTWLFETTPIH:1306

*Rn* Xinβ QMNKQLFESEGGDKKNYVRTVSINEIQKGNVKTSTWLFETHSIDEL.GEVSTYENIKTVTQEDVQKGDVKQAVWLFENQTLDSIKELDESDTKITK..EEIPPSDVKTTTWLFETTPIH:1304

*Md* Xinβ KYHKQLFESEESEKRTHIRTVSVNEIQQGNVKTSTWLFETHTLDELRGEGSEYEHIKTVTKEDVQKGDVKQAVWLFENQTLDSIKETDEYFTEVTR..EEIPHSDVKTTTWLFETTPLH:1355

*Ac* Xinβ KANKKLFESEEAQEGMYVRTVSVSEIQHGNVKTSTWLFETHTIDEIRGEESEYKGVKTVTKEEVQEGDVQHSVWLFENQPLDSIKETDESDGKIDK..EEIPQADVKTTTWLFETTPFH:1219

*Xt* Xinβ KGNKQRFEEEDDTNNKYVRTVSISEIKQGNVKTSTWLFETHSVDDI..HEEDYQNIKTVRKEDINKGDVKEAVWLFENQNLDSLKEEVENVREIEK..ESIPHADVRTTTWLFETTPLH:1294

*Tn* Xinβ2 .......................................................................................................................:457

*Tr* Xinβ46 RKNKDHFESDALSQG.SVRTVNVSEIQKGDVRTAKWRFETQSIDKIRSMSSENL.IETVKTEEVAKGDVKHSVWLFEKNPLDHIKEVDEDEDHRTVTQEEISKADVKTTMWLFETTPFD:1408

*Ga* Xinβ16 RINKDRFESDRGFQE.SVRTVNVSEIQKGDVRSAKWRFETQSIDKIRSMSSENL.IETVKKEEVERGDVKHSVWLFEKNPLDHIKEVDEDQGTTSTSPEAIFKSDVKTTAWLFETTPFD:1266

*Ol* Xinβ21 RLSKHQFETDGLSQG.SVRTVNVSEIQKGDVRTAKWKFETQSIDKIRSMSSENV.VETVKKEEFEKGDVKHSVWLFEKKPLDHIKQADEDENKPAT.QEEIPKADVKTTTWLFETTPFD:1271

*Dr* XinβNA KTNKQRFESDAMSQK.LVRTVSMSEIHKGDVRTAKWMFETHTIDQIHAEKSEEE.MKTVVLEEQLKGDVKQSVWLFEKNPLDHINESEEQHIQVVS..EDIPKADVKSTTWLFETTPFT:1177

*Tn* Xinβ3 KTNKQRFETDEMAQK.YIRTVSVSEIQKGDVRSATWMFETRTIDEIHGKGVEYDGMETVTKEEVMKGDVKQSVWLFEKQPLDSIKDSDGTELIVTK..EEIPQADVKTTTWLFETTPFH:1022

*Tr* Xinβ36 KSSKERFESDEMSQK.YIRTVSVSEIHKGDVRSATWMFETRTIDEIHGKGVEYDGMETVTKEEVMKGDVKQSVWLFEKQPLDSIKDSDGTEIVVTK..EEIPQADVKTTTWLFETTPFH:1554

*Ga* Xinβ1 KTNKQRFETDEMSQR.YVKTVSVSEIQKGDVRSATWMFETRTIDEIRGEGAEYDGMERVTKEEVMKGDVKQSVWLFEKQPLDTIKETDGTELVVTK..EQIPQADVKSTTWLFESTPFN:1317

*Ol* Xinβ2 KTNKQRFETDETSQK.YVRTVSVSEIQRGDVRSATWMFETRTIDEIRGDGVEYDGMEKVKKEEVMKGDVKQSVWLFEKHPLDSIRESEDSETAVSK..EEVLRGDVKTTTWLFETTQLH:1280

*Dr* Xinβ6 KTNKQRFETDDLSEK.YVRTVSVSEIQRGNVRSSTWMFETRTIDKINAEGSEYEGMEKVMREEVVKGDVKQSVWLFEKEPLDRIKDVDDTETVISR..EEIPKADVKTTTWLFETTPLT:1610

*Dr* Xinβ19 KTNKQRFETDDLSEK.YVRTVSVSEIQRGNVRSSTWMFETRTIDKINAEGSEYEGMEKVMREEVVKGDVKQSVWLFEKEPLDRIKDVDDTETVISR..EEIPKADVKTTTWLFETTPLT:1352

*Pm* Xin QSNAQLFESEGVDRRKFVRTVSVSDVQRGDVRTSRWLFETKNIDEIGESASEKLDFTKVIREDVKSGDLKNSLWLFENHPLDKIKEQDEETTVRAE...EIQGGDVKTTTWLFERTPLH:1175
