# Supplementary material for: Emergence of Xin Demarcates a Key Innovation in Heart Evolution
Source: PLoS One. 2008 Aug 6;3(8):e2857. doi: 10.1371/journal.pone.0002857 (PMC2478706; doi:10.1371/journal.pone.0002857)
Supplement: Figure S2 — Multiple sequence alignment of the beta-catenin binding domain of Xin proteins. Located within the conserved Xin repeat region, the beta-catenin binding domain previously characterized from aa#535–636 of mXinalpha was used to identify the homologous region in other Xin proteins and aligned with CLUSTAL W. The positions of the 17th–19th Xin repeat units (XR17–XR19) are highlighted above the aligned sequences. Identical residues are highlighted in black with white letters, conserved residues are highlighted in dark grey with white letters, and similar residues are highlighted in light grey with black letters. (0.05 MB DOC) [file pone.0002857.s002.doc]

______XR17 _____ ______XR18______ ______XR19__

*Hs* Xinα IDVVRGITRQEVVAGDVGTARWLFETQPLEMIHQREQQERQKEEGKSQGDPQPEAPPKGDVQTIRWLFETCPMSELAEKQGSEVTD...PTAKAEAQSCTWMFKP:632

*Pt* Xinα IDVVRGITRQEVVAGDVGTARWLFETQPLEMIHQREQQERQKEEGKSQGDPQPEAPPKGDVQTIRWLFETCPMSELAEKQGSEVTD...PTAKAEAQSCTWMFKP:632

*Mam* Xinα IDVVRGITRQEVVAGDVGTARWLFETQPLEMIHQREQQERQKEEGKSQGDPQPAAPSKGDVQTIRWLFETCPMSELAEKQGSEVTD...PTAKAEAQSCTWMFKP:632

*Cf* Xinα VDVVRGITRQEVVAGDVGTARWLFETQPLEVIHQRERQGRQEEEGKSQAGSQPEALPKGDVQTIRWLFETCPMSELAEKQGPEVTE...PTAKAKPRSCTWMFAP:637

*Ec* Xinα VDVVRGITRQEVVAGDVGTARWLFETQPLEVIHQREQQERQEEEGKSQAGPEPDAPLKGDVQTIRWLFETCPMSELAEKQGSEVTD...PTTKAKERSCTWMFTP:637

*Bt* Xinα VDVVRGITQQEVVAGDVGTTRWLFETQPLEVIHQREQQEREEEEGKPQGGPQPEIPHKGDVQTIRWLFETCPMSELAERQGSEVTD...LTSKAR..SCTWMFAP:634

*Mm* Xinα IDVVRGITRQEVVAGDVGTTRWLFETQPLEMIHQQEQQKPEEEEGKGPGGPPPELPKKGDVQTIRWLFETYPMSELAEKRESEVTD...PVSKAETQSCTWMFGP:636

*Rn* Xinα VDVVRGITRQEVVAGDVGTTRWLFETQPLEMIHQQEQQKREEEEGRGPGGPPPELPQKGDVQTIRWLFETYPMSELAEKQESEVTG...PVSKAEAQSCTWMFGS:635

*Md* Xinα VDVIRGITRQEVMAGDVSSAHWLFETQPLEVIHRQKKEPEEKE.......PPEAAPPKCNVQTVRWLFETQPIHALAQEPRPEVPG...TSPAADVRSCTWMFDM:629

*Gg* Xinα VDIIRGITKQEVVAGDVRTAKWLFETQPMDVIHHQATQGEEHPSMK......REISQRGDVKTCRWLFETQPMHTLYEKAEKKQEE.DVSVPQADVKSYTWMFET:669

*Ac* Xinα VALVKGITKQEVVAGDVKTAKWLFETQPIDVIHSQMNPADQDISEK......GEATQKGDVKTCRWLFETHPMDTLYEKEGEKQDG.EECVPQGDVKSCTWMFET:675

*Xt* Xinα VDIIRGITKEEIVSGDVGTAKWLFETQPVDIVHQQSDATEKHSSVQ......KQVLQKGDVKKCKWLFETQPIDKLYDKSEQTKDT.EAQV.QGDVKSYTWMFES:566

*Tn* Xinα15 VEMIKGITRQEQEIGDVKMVKWLFETQTIDGVQSRLKQRDDGAS..........GGKRGDVQTCKWFFETEPMNILHDETEKRNDN..QATKGAGDRTTTWLFES:640

*Tr* Xinα296 VEIIKGITRQEQETGDVRMAKWLFETQTIAGIQAKFNQRDDNAS..........EVEKGDVKTRKWLFETKPMKILHDKTEKPNDN..EPPKGADVKSVTWLFES:640

*Ga* Xinα3 VEVIKGITREEDTMGDVKMAKWHFETQTIDGIHSKFNQTERDAS.........VEHRKGDVNNCKWLFETQPMNIVDEKSEKMNDK..EATDNTNVKSITWLFES:649

*Ol* Xinα17 VEVIKGITRQEECTGDLNMAKWLFETQTIDGIHCKVNQTEKTSSDR.......EELCKGDVKTCKWLFETQSMDVQCEKDERLKDQ..DLTDGTNVRSITWLFES:644

*Dr* Xinα2 VELIKGITRQEDMTDDTRTAKWMFETQPLDCMS..LNSRTDVDSTQ.......KEFKKSNVKTCKWLFETKPMDMLYEKSEGKQDV..EPVPKADVKSHTWLFET:649

*Hs* Xinβ FQIIRGISAQEIQTGNVKSAKWLFETQPLDSIKYFSDVEETESKTE.....QTRDIVKGDVKTCKWLFETQPMESLYEKVSLMTSS..EEIHKGDVKTCTWLFET:1133

*Pt* Xinβ FQIIRGISAQEIQTGNVKSAKWLFETQPLDSIKYFSDVEETESKTE.....QTRDIVKGDVKTCKWLFETQPMESLYEKVSLMTSS..EEIHKGDVKTCTWLFET:1064

*Mam* Xinβ FQIIRGISAQEIQTGNVKSAKWLFETQPLDSIKYFSDVEETESKTE.....QARDIIKGDVKTCKWLFETQPMESLYEKVSLMTSS..EEIHKGDVKTCTWLFET:1139

*Cf* Xinβ FQIIRGISAQEIQTGNVKSAKWLFETQPLDSIKYFSNMEEVESKTE.....QVTDIVKGDVKTCRWLFETQPMESLYEKVSLMTGS..EEIHKGDVKACTWLFET:951

*Ec* Xinβ FQIIRGISAREIQTGNVKSAKWLFETQPLDSIKYFSNMEEVESKTE.....QATDIVKGDVKTCRWLFETQPMESLYEKVSLMTGS..EEIHKGDVKACTWLFET:905

*Bt* Xinβ YQIIRGISAQEIQTGNVKSAKWLFETQPLDSIKHFSNMEEVERKTE.....QVTDIVKGDVKTCRWLFETQPMESLYEKVSLMTGS..KEIHKGDVKACTWLFET:953

*Mm* Xinβ FQIIRGISAQEIQAGNVKSARWLFETQPLDSIKYFSNVEETDSKTE.....QSTDIVKGDVKTCKWLFETQPMESLYEKASLMTNS..EDIHKGDVRTCMWLFET:905

*Rn* Xinβ FQIIRGISAQEIQAGNVKSARWLFETQPLDSIKYFSNVEETDSKTE.....QSTDIVKGDVKTCKWLFETQPMESLYEKASLMTNS..EDIHKGDVRTCMWLFET:903

*Md* Xinβ YQIIKGISSREIQSGDVKSAKWLFETQPLDSIKHFNSMEDSESTKQ.....QTMDVVKGDVKAYTWLFETQPMETLYDKTELITNN..EEIQKGDVKTCTWLFET:952

*Ac* Xinβ IEIIKGISSQEVQSGDVKTAKWLFETQPLDSIKYFAQMEDEESTEES....QATEVVKGDVKTCRWLFETQPMETLYDKETIESDS..EEIHKGDVKTCTWLFET:814

*Xt* Xinβ YQIIKGISSEQIQSGDVKTGKWLFETQPLDSIKYFSNAEDEEIITE.....NQTDIVKGDVKMCKWLFETQQMEDLYEKQDKISAD..VDIQRGDVKTCTWLFET:895

*Tn* Xinβ2 YHVIKGISKQQIESGDVKTAKWLFETQPLDSIKYFSNIEDEEVVET.....NNIDILKGDVKTCKWLFETKPMDILYQKVELEGEG.STEVQKGDVKTCTWLFET:308

*Tr* Xinβ46 YQVIKGISKHQIESGDVKTAKWLFETQPLDSIKYFSNIEDDEAVGTS....NNIDIVKGDVKTCKWLFETKPMDILYEKVKLEDEG.SEEIQKGDVKTCTWLFET:1063

*Ga* Xinβ16 YQVIQGISKQEIESGDVKTAKWLFETQPLDAIKYFSNIEDEEVTGAK....INLDQVKGDVKTCKWLFETKPMDVLYERAELKGENESEEMQKGDVKTSTWLFET:864

*Ol* Xinβ21 YQIIKGISKQEIESGDVKTAKWLFETQPLDAIKYFSNIEDEETVGGI....KSLDVVKGDVKNCRWLFETKPMDALYERVEKESIDETEEVRKGDVKTCTWLFET:866

*Dr* XinβNA FQIIKGITQQEVESGDVKTAKWLFETQPLDAIKYFSNIEDDECVTK.....ETADIVKGDVKTCKWLFETKPIDTLYERKELESTKDSDEVQKGDVKTCTWLFES:775

*Tn* Xinβ3 FQIIKGISKQEIQSGDVKTARWLFETQQLDAIKYFNNFEDEEHKIK.....EGGEIEKGDVNTCRWLFETHPLDVLYEKVEKNEAD.IKEVEKGDVKTCTWLFET:620

*Tr* Xinβ36 FQIIKGISKQEVQSGDVKTAKWLFETQQLDAIKYFNNFEDEEHEIK.....EGTEIEKGDVKTCRWLFETRPLDGLYDKVEKNEAD.IEEVAKGDVKTCTWLFET:1152

*Ga* Xinβ1 FQIIKGISKQEVESGDVKTAKWLFETQALDSIKSFGQCEDEEHACK.....EGIEIEKGDVKTCRWLFETQPMDALYEKMEKSEVD.VEEVQRGDVKTCTWLFET:911

*Ol* Xinβ2 FQIIKGISKQEIESGDVKTAKWLFETQPLDAIKYFSNAEVKEQKTK.....E..EIEKGDVKTCRWLFETQPMDVLYEKVDRSETD.VKEVHKGDVKTCTWLFET:875

*Dr* Xinβ6 FQIIKGISKEEIQSGDVKTAKWLFETQPLDGIKYFN.LEEEDNRKN.....ESIEIQRGDVKTCRWLFETQPMDVLYEKIETKTED.TTDIQKGDVKTCTWLFET:1204

*Dr* Xinβ19 FQIIKGISKEEIQSGDVKTAKWLFETQPLDGIKYFN.LEEEDNRKN.....ESIEIQRGDVKTCRWLFETQPMDVLYEKIETKTED.TTDIQKGDVKTCTWLFET:946

*Pm* Xinα IQIIKGISKEELQAGDVKTAKWLFETHPLDTIKNAD...EESVTQQ......RTEVHRGDVKMCRWLFETRPMDALYEKVEKREGE..EEVMKGDVKTCTWLFEN:737
