# Supplementary material for: Emergence of Xin Demarcates a Key Innovation in Heart Evolution
Source: PLoS One. 2008 Aug 6;3(8):e2857. doi: 10.1371/journal.pone.0002857 (PMC2478706; doi:10.1371/journal.pone.0002857)
Supplement: Figure S3 — Multiple sequence alignment of the Mena/VASP-binding domain and putative DNA-binding domain sequences. The Mena/VASP-binding domain (EDLPLPPPPALED) was previously mapped to aa#18–46 of hXinalpha and two acidic residues each at the N- and C-termini were essential for its binding activity. Based on this characteristic, the Mena/VASP-binding domain is a derived trait found only in the placental mammal Xinα lineage that seems to have arisen from a deletion within the terrestrial Xinα N-terminal proline-rich region. Meanwhile, the putative DNA-binding domain (#47–69 of mXinalpha) identified by the high similarity with the Myb-A and Myb-B DNA-binding domains [1] appears to be an ancestral trait that is highly conserved in most Xin proteins, except Xt Xinalpha28, Tn Xinbeta2, Tn Xinbeta3 and Tr Xinbeta36. Identical residues are highlighted in black with white letters, conserved residues are highlighted in dark grey with white letters, and similar residues are highlighted in light grey with black letters. (0.05 MB DOC) [file pone.0002857.s003.doc]

Mena/VASP-binding domain DNA binding domain

*Hs* Xinα .........TAEDLPLPPPP................ALEDLPL...PPPKESFS......KFH...QQRQASELRRLYRHIHPELRKNL:69

*Pt* Xinα .........TAEDLPLPPPP................ALEDLPL...PPPKESFS......KFH...QQRQASELRRLYRHIHPELRKNL:69

*Mam* Xinα .........TAEDLPLPPPP................ALDDLPL...PPPKESFS......KFH...QQRQASELRRLYRHIHPELRKNL:69

*Cf* Xinα .........AAEDLPLPPPP................ALDDLPL...PPPKESFS......KFH...QQRQASELRRLYRHIHPELRKNL:69

*Ec* Xinα .........TAEDLPLPPPP................ALEDLPP...PPPKESFS......KFH...QQRQASELRRLYKHIHPELRKNL:69

*Bt* Xinα .........ATEDLPLPPPP................ALEDLPP...PPPKESFS......KFH...QQRQASELRRLYKHIHPELRKNL:69

*Mm* Xinα .........TEEDLSLPHPS................APEGLPP...PPPKETFS......KFQ...QQRQASELRRLYKHIHPELRKNL:69

*Rn* Xinα .........TGEDLPLPPPS................ALEGLPP...PPPKESFS......KFQ...QQRQANELRRLYKHIHPELRKNL:69

*Md* Xinα .........SAMTFPKPGED.................APPLP.....PPKEAFS......KFY...QQRQASELRRLYRHIHPELRKNL:58

*Gg* Xin .........MEDDLPPPPIP...DSIQVIA..PASQDPNPLPV...PPPKQAFS......KFY...QQRQVNELKRLYRHMHPELRKNL:77

*Ac* Xinα .........IDENLPPPPPPPSQDSAPGAASIARSQNSSPLPP...PPPKETFS......KFY...QQRQVNELKRLYRHMHPELRKNL:84

*Tn* Xinα15 .........DEEDLPPPPPPLVPPRPLDHEGP..GS..STPPP...PPPKETFA......TFC...QQRQKSELKRLFKHIHPELRAHL:78

*Tr* Xinα296.........DEEDLPPPPPPLVPPRPLDYEGP..EL..STPPP...PPPKETFT......AFC...QQRQKSELKRLFKHIHPDLRAHL:78

*Ga* Xinα3 ...........DDLPLPPPPPVPPKPHDYEGP..SA.ITNLPL...PPPKETFS......TCY...QQRQKSELKRLFKHIHPDLRASL:80

*Ol* Xinα17 ...........EDLLPPPPPPVPPRPTDYED...A...TALPV...PPPKETFS......TFY...QQRQKSELKRLFKHIHPDLRAGL:77

*Dr* Xinα2 .........GDEDFPPPPPPLPRPQVLESLQK..DLSQNFLPV...PPPKETFS......EIY...QQRQKSELKRLFKHIHPELKMTV:81

*Hs* Xinβ ..NATSSGMTEEFPPPP.PDVLQTS.......VDVTAFSQSPEL..PSPPRRLP..VPKDVYS...KQRNLYELNRLYKHIHPELRKNL:513

*Pt* Xinβ ..NATSSGMTEEFPPPP.PDVLQTS.......VDVTAFSQSPEL..PSPPRRLP..VPKDVYS...KQRNLYELNRLYKHIHPELRKNL:444

*Mam* Xinβ ..NATSSGMTEEFPPPP.PDVLQTS.......VDATAFSQSPEL..PSPPRRLP..VPKDVYS...KQRNLYELNRLYKHIHPELRKNL:519

*Cf* Xinβ ..NATPMGKTEEFPPPP.PDIFQTP.......VDVTAFSQSPEC..PNPPRIPL..VPKELYS...KQRNLYELNRLYKHIHPELRKNL:332

*Ec* Xinβ ..NATPLRKTEEFPPPQ.PDILQTP.......IDVTAFSQSPEV..PSPPRIPP..VPKELYS...KQRNLYELNRLYKHIHPELRKNL:284

*Bt* Xinβ ..NATPSEKTEEFPPPP.PDMLQPP.......VDVTAFSQSPEL..PNPPRKPP..VPKELYS...KQRNLYELNRLYKHIHPELRKNL:332

*Mm* Xinβ ..NGTPSGKMEEFPPPP.PDVCQTP.......MDVTAFSQSPEF..PSPPRRLP..MPKDLYS...KQRNLYELNRLYRHIHPELRKNL:284

*Rn* Xinβ ..KGTPSGKMEEFPPPP.PDVFQTP.......MDVTAFSQSPEF..PSPPRRLP..MPRDVYS...KQRNLYELNRLYRHIHPELRKNL:282

*Md* Xinβ ..NTTNFGKTEDFLPPP.SKVVPTL.......EDVTAFSQSPEC..PKPPGKCP..IPKDLYS...KQRNLYELNRLYKHIHPELRKNL:330

*Ac* Xinβ ..TSSKYGSTEEFPPPPSPDLLQAP.......SEMTGFSQSPEP..SPSPSKQP..FPKDVYS...KQRNLYELKRLYKHIHPELRKNL:192

*Xt* Xinβ ....ASFGSLEEFPPPPPPNILET........EELADFSQSPEP..PSYAEQQPCAMSKELYA...KQRNLYELKRLYKHIHPELRKNL:283

*Tr* Xinβ46 ..PPPPPAEDADYLPPPPPDLLQMP..ESED.VPACQDFPEPPEPLNPFKDPFNR-...EAFC...KQRRMNELKRLYKHIQPDISNIE:344

*Ga* Xinβ16 ...PPPPPEDSGYLPPPPPDLLEMPS.DSEN.IPACHYSPQPPEPTKLSKYPINK....EAYC...KQRSMAELKRLCKHIHPEVRKNI:266

*Ol* Xinβ21 ..............PPPPPDLLQIP..ESEH.IPESHFSQEPPNPVNPFKQSHNR....EAYV...KQRGMSELKRLYKHIHPEVRKNI:144

*Dr* XinβNA DDQYIDDEEDLEYLPPPPPDLLEEP.......SDDAEIFPEPP..LQPVKHTVNR....EQHF...RQRELMELKRLCKHIHPDVRKDL:162

*Ga* Xinβ1 ASASSFAYELTDHFPPPPSNLP........QDTPDHVSS.QPQEPASQHKHANT....KEQYF...KHKSTAELKRLYKHINPEVRKNL:287

*Ol* Xinβ2 SSASSMHNEATDPFSLPSSHLQ........VKTGESRS..QSQESASRESHTGS....TEQYF...KHKNMAELKRLYKHIHPEVRKNL:258

*Dr* Xinβ6 SSMASSQYEAVEHFSQPISATQQFQ...ETSQVPERCPSPKPTDASTSSKYAVN....KEQYS...KQRNLYELKRLYKHIHPEVRKNL:582

*Dr* Xinβ19 SSMASSQYEAVEHFSQPISATQQFQ...ETSQVPERCPSPKPTDASTSSKYAVN....KEQYS...KQRNLYELKRLYKHIHPEVRKNL:324

*Pm* Xin ..............PEP..GFLTVP.......QERAAMAPSPE......PHARV.....AYR....QQQQLNELRRLYKHIHPEVRKNL:132
