# Supplementary material for: Emergence of Xin Demarcates a Key Innovation in Heart Evolution
Source: PLoS One. 2008 Aug 6;3(8):e2857. doi: 10.1371/journal.pone.0002857 (PMC2478706; doi:10.1371/journal.pone.0002857)
Supplement: Figure S4 — Multiple sequence alignment of the C-terminal region immediately downstrean of the Xin repeat region. The entire C-terminal region of the Xin proteins (sequence immediately after the Xin repeats) was aligned to identify regions of high conservation. From this alignment, the sequence region containing aa#743–1,083 of mXinalpha and aa#1,307–1,705 of mXinbeta and their homologous regions from all Xins revealed a high conservation among all the Xinβ proteins and the putative ancestral Pm Xin. There is a high degree of similarity in this region among mammalian Xinalpha proteins, which is much divergent from the same region of all Xinbeta proteins. Interestingly, there is a G(D/N)(V/I/L) motif repeated 10 and 13 times within the conserved C-terminus of all Xinβ proteins and the teleost Xinβ respectively (indicated by * below the alignment). Unexpectedly, this G(D/N)(V/I/L) motif is very similar to the first three amino acids of the Xin repeat unit. (0.22 MB DOC) [file pone.0002857.s004.doc]

*Hs* Xinα ..SLAAESIQGGN.LLEEQPMSPSGNRMQESQETAAEGTLRTLHATPGILHHGGILMEARGPGELCLAKYVLSGTGQGHPYIRKEELVSGELPRIICQVLRRPDVDQQGLLVQEDPTGQ:854

*Pt* Xinα ..SLAAESIQGGN.LLEEQPMSPSGNRMQESQETAAEGTLRTLHATPGILHHGGILMEARGPGELCLAKYVLSGTGQGHPYIRKEELVSGELPRIICQVLRRPDVDQQGLLVQEDPTGQ:854

*Mam* Xinα ..SLAAESIRGGN.LLEEQPMSPSGNRMQESQETAAEGTLRTLHATPGILHHGGILMEARGPGELCLAKYVLSGTGQGLPYIRKEELVSGELPRIIRQVLRRPDVDQQGLLVQEDPTGQ:854

*Cf* Xinα ..SLAAESIRGGN.LPEEQPTGPSGSRVPERQETGAEGTLRTLHTLPGLLHDGGILMEVRGPGELCLAKYVLPGPGEGGPRVRKEELVSGELPRIVRQVLRRPDVDQQGLLVQEDPAGQ:858

*Ec* Xinα ..SLAAESIRGDN.LQEEQPVGISDNGVLERQKTIAEGTLWTLHATPGILHHGGILMEARGPGELCLAKYMLPSPGQGGPYIRKEELVSGELPRIVRQMLRRPDVDQQGLLVQEDPTGQ:858

*Bt* Xinα ..SLAAESIRGGN.LQEEQPVGPSGKGVPERQETAAEGTLRTLHATPGVLHHGGILMEARGPGELCLARYVLPCPGQDSPHVRKEELVSGELPRIVRQVLRRADVDQQGLLVQEDPAGQ:856

*Mm* Xinα ..SLAAESIRGDN.LQEEQPKGSAGHGTPERQETAAERTLRTLHATPGILHHGGILMEARGPGELCLAKYVLPSPGQGRPYIRKEELVCGELPRIVRQVLRRTDVDQQGLLVQEDTAGQ:858

*Rn* Xinα ..SLAAESVSGGN.LQEEQPKVSADPGTPERQETAAERTLRTLHATPGILHHGGILMEARGPGELCLAKYVLPSPGQGRPCVQKEELVCGELPRIVRQVLGRTDVDQQGLLVQEDTAGQ:857

*Md* Xinα LEKAATEGIQEMPPDQDAAATAQAGKRFPEPRETAAAETLRALGSSPGVLQRGGILIEAHGARTVRMAKYQLPSPGEGQPQVQKEEVVLGQLPRILSRVMRGPNVAPQGLLVQEDEAGL:856

*Gg* Xin ..................KFSASECSRETELKERTMRETLETLCT.CQAIQHDGILIEANDTESVKMVKYQLSSPGA..PEILKEEIVRGHLQGIMLQLLHRTNVEPQSVLVEEDREGK:1168

*Ac* Xinα ..................KLSTTRHCAEKDATQRAITSTLEVLFS.RQVVKHGGILIEANDTESITMAKYHLGPHGS..PEVQKEEVMGGNLQNILMQLLQRSNIEAKGELVQEEENGE:1179

*Xt* Xinα .....................KFERATSFEQTEVNVKDTLEKCLS.YKIIEHKGIAIESVDERSVKMVKYQPTHAG...TLIQKEETLGGNLQRILLQLLHRADLEQHGMLVEENNMGE:1057

*Tn* Xinα15 ....................................VDTLSYLCQ.RGFVHSSGIIIEAKESRNVNMAKYQVDNNKG..VQIQKEELVRGNIINIMLQLLIEPTLKPQISLLREAEKGK:1128

*Tr* Xinα296.............................KITAHTVADTLYDLCQ.LGFIHSSGIIIEANESRNVNMAKYQFDSNNG..VQIQKDELVGGNIVNIMLQLLIKPTLKPQISLLREGEKGK:1128

*Ga* Xinα3 .............................AYQLQNIVQKIDELLV.QDKDLKKGIIMQETAGGQAELSVYSLIGNSE..IKMEGHAVERGDIKSTIGNLLSSASTQRTAVSCRVDENEK:1141

*Ol* Xinα17 .............................KITANSVADTLSYLHQ.NHFVQSSGIIIETNEGKSITMAKYLLESNTG..VQIQKEDVVQGNISNIMLQLLLTPTLAPQITLLRQTEKGE:1130

*Dr* Xinα2 .............................KITVESVTDILYRLCH.NSFIHSSGIIIQANDYKYVNMAKYQIMKDEG..PKVLKEEVVEGNIRNLMLQLLFKPNIKPMVVLLKEDEQGK:1135

*Hs* Xinβ .................EFNET..RVEKIEIIGKSIKETLEDLYSQKVIQA.PGIIIEADEIGDVRMAKYKLMN.QAS.PEIQKEEIIRADLRNIMVNLLSKRDCTEREILISEEEKGN:1632

*Pt* Xinβ .................EFNET..RIEKIEIIGKSIKETLEDLYSQKVIQA.PGIIIEADEVGDVRMAKYKLMN.QAS.PEIQKEEIIRADLRNIMVNLLSKRDCTEREILISEEEKGN:1563

*Mam* Xinβ .................EFNEN..RIEKIEIIGKSIKKTLEDLYSQKVIQA.PGIIIEADEVGDVRMAKYKLMN.QAS.PEIQKEEIIRADLRNIMVNLLSKRDYTKREILVSEEEKGN:1638

*Cf* Xinβ .................EFNES..RVEKVEIIGKSIKETLEELYSQKVIEA.PGIIIEAGEVGDVRMAKYKLMN.QAS.PEIQKEEIIRVDLRNIIMNLLSKRDCTRREIWVSEEEKGN:1450

*Ec* Xinβ .................EFNEN..RVEKVEIIGKNIKETLEELYSQKVIEA.PGIIIEADEVGDVRMAKYKLMN.QES.PEIQKEEIIRVDLRNIIMNLLSKRDCTKREILVSEEEKGN:1404

*Bt* Xinβ .................RFTEN..RVEKVEIIGKSIKETLGELYSQKVIEA.PGIIIEADEVGDVRMAKYKLMN.QAS.PEIQKEEIIKVDLRNIMVNLLSKRDCKKREILVSEEEKGN:1452

*Mm* Xinβ .................EFNKT..RVEKEEIIGKSIKETLEDLYSQRVVEA.PGIIIEADEVGDVRMAKYKLMN.QTT.PEIQKEEVIRADLGNIMMNLLSQRDCTKKEIFVSEEEKGN:1403

*Rn* Xinβ .................EFNET..RIEKEEIIGKSIKETLEDLYSQRVVEA.PGIIIEADEVGDVRMAKYKLMN.QRT.PEIQKEEVIRADLGNIMMNLLSQRDCTKKEIFISEEEKGN:1401

*Md* Xinβ .................EFNEN..KVEKEEIIGKSIKKTLEELYSQKMIES.HGIIIEENEVGDVRMAKYKLMN.QES.PEIQKEEVIRGDIRTIMMNLLSKRDDAKREILVSEEEKGN:1452

*Ac* Xinβ .................EFNES..NVQKEEIIGKSVKETLKELYSHKVVET.HGIILESDEIGDVRMAKYKLMN.QEP.PEIQKEEIIKGDLANIILNLLSRPSTEEKENKVNEDEKGN:1316

*Xt* Xinβ .................EFNEH..AFEKQEIIGKSIQGTLKELYSHKIVES.HGIILEADEIGDIRMAKYNLMN.QDS.PKIQKEEIIRGDLQSIMMNLLSENSSAKRIIMLNDEEKGN:1391

*Tn* Xinβ2 .......................................................................................................................:457

*Tr* Xinβ46 .................SFNES..KIERTEILGKSVKGTLEELYSQKMVRS.KGILIEADEIGDVRMAKYQLMN.KQA.PEIQREEVIKCDIQTVMMNLLNTQVKQKQHISIDTEEKGN:1505

Ga Xinβ16 .................DFNET..KVEKTEIVGKSVKGTLAELYSQQMVKS.KGILIEADEIGDVRMAKYQLMN.KEA.PEIQREDVIRGDLQTIMMNLLNRGETHEQQIVIDSDEMGN:1363

*Ol* Xinβ21 .................EFHEA..KTEKTEILGKSVKETLEELYSQKMVRS.KGILIEADEIGDVRMAKYQLMN.KEA.PKIQREEVIRGDLQNIMMNLLNTKETKEQQIVIDSEEKGN:1368

*Dr* XinβNA .................EFNES..RVEKTEIVGKSVKATLDELYTQKMVDS.KGIIIEADEIGDVRMAKYNLMN.KDA.PEIQKEEIIKGDLQNIMINLLNRQEKTEEKIIINAEERGD:1274

*Tn* Xinβ3 .................DFNET..KTEHKEIIGKSIKETLEELYCQKMVES.QGILIEADEIGDVRMAKYKLMN.QEA.PQIQKEEIIRGDLSNIMMNLLTRREFAEKGITIDKEERGN:1119

*Tr* Xinβ36 .................DFNET..KAEQKEIIGKSIKETLEELYCQKMVDS.QGILIEADEIGDVRMAKYKLMN.QEA.PQIQKEEIIRGDLSNIMMNLLTRREISEKGITIDKEERGN:1651

*Ga* Xinβ1 .................EFNES..EMKRTEIMGKSIKDTLEELYSQKMVES.QGVLIEADEIGDVRMAKYNLMN.QKA.PQIQREETIRGDLSNIMMNLLSRREVTERGITIDSEERGN:1414

*Ol* Xinβ2 .................QFNES..SVEKTEIIGKSIKETLEELYSQKMVES.QGVLIEADEIGDIRMAKYKLLN.QEA.PQIQKEDIIRGDLSNIMMNLLNRRETSERGITIDKEERGD:1377

*Dr* Xinβ6 .................EFNES..SMERTEIVGKSIKTTLEELFSQKMVDS.QGILIEADEIGDVRMAKYKLLN.QDT.PEIQREEVIHGDLNNIMMNLLNRRETTERGITINMQERGN:1707

*Dr* Xinβ19 .................EFNES..SMERTEIVGKSIKTTLEELFSQKMVDS.QGILIEADEIGDVRMAKYKLLN.QDT.PEIQREEVIHGDLNNIMMNLLNRRETTERGITINMQERGN:1449

*Pm* Xin .................RLGDSGTYVEKPEIVGKNIRETLRSLIDCNVVDSSRGILLESSDVGNVRMAMYNLMNPEAQ.PQIQKDEIIRGDMHSILQQLLSKKREDHQSIVIDSEERGN:1276

*** *** **

*Hs* Xinα LQLKPLRLPTPGSSGNIE.......DMDPELQQLLACGLGT.SVARTGLVMQETEQG.....LVALTAYSLQP................................RLTSKASERSSVQLL:929

*Pt* Xinα LQLKPLRLPTPGSSGNIE.......DMDPELQQLLACGLGT.SVARTGLVMQETEQG.....LVALTAYSLQP................................RLTSKASERSSVQLL:929

*Mam* Xinα LQLKPLRLPTPGSSGSIE.......DMDPELQQLLACGLRA.SVARTGLVMQETEQG.....LVALTAYSLQP................................RLTSKASERSSVQLL:929

*Cf* Xinα LRLKPLRLPAPGGSGNIE.......DTDSEFQQLLACGLGT.SVARTGLVMQETEQG.....LVSLTAYSLQP................................QPNSRAPERGSVQLL:933

*Ec* Xinα LRLKPLRLPAPGSGGNVE.......DMDPEFQQLLACGLGT.SVARTGVVMQETDQG.....LVSLTAYSLQP................................RLTSRAPERSSVQLL:933

*Bt* Xinα LHLKPLKLPAPGSSGSIE.......DMDPEFQQLLACGLGT.SAGRTGLVMQETERG.....LVALTAYSLQP................................WLASRAPERSSVQLL:931

*Mm* Xinα LQLHPLTLPGPGDPGNIE.......DMDPELQQLLACGLGV.SVSKTGLVMQETGQG.....LVALTAYSLQP................................QLTSRAPERSSVQLL:933

*Rn* Xinα LRLHPLKLPGPGDPENIE.......DMDPELQQLLACGLGV.SMAKTGLVMQETGQG.....LVALTAYSLQP................................QPTSRGPERSSVQLL:932

*Md* Xinα KLEPLELSALADG............CGHEVWDLLAQSN...AGARTGLVIQEAEQR.....LVKLVVYSLRLP...PG.........................VQAAGGAAEKGNVQLL:928

*Gg* Xin IKVSSLQLLDQSEAIKGK......EDLSGNVAKALQSLLSQDASIKKGMVIQETKSESVKMTLYSLLFHSVQQK.......VVKGDVKSTIGNLMASSQEQRATVTVKREDNEKGNVQLF:1275

*Ac* Xinα IRVRPVQLLDTGKAEKSR......EDLKDDVAKALQDLLSQGTSIKKGLLMQETETGSAKITIFSLLHRIDQDH...S...LVKGDVKSTIGNLLASSQEQKATATIRREDNEKGNVQLY:1287

*Xt* Xinα LHRTNLQLIQREGQEEDR......Q.VQDNVSKALQILLNEDVLVKTGIIMEETEKGSVRIIIYSVSKYIQRDT...SGESIVKGDVKSTIGNLLHN.QERERKVSVTREQHERGNVQLY:1166

*Tn* Xinα15 INTAVVELPVYQSTGSINLQ...SDQRFQNIVQMINELLSQD.SMTTGIIMQETSGGQAEMSVYSLICNSSTKT...EN.........................................:1200

*Tr* Xinα296 INTTVVELPVYQSTATVNLE...SDQRVQNIVQMINDLLIQD.SMTTGIIMQETTGGQAEMSVYSLICNSETKT...ESYVRERGDVKSTIGNLLATANIQKSVASCKVDENEKGNVNLF:1241

*Ga* Xinα3 GNVNLYKSCIEK.......................GDLDYLK.SLQADATADEVD................HSH...LA.........................................:1177

*Ol* Xinα17 ANTTVVELPVLELTDSTTFE...SDQRARNIVQTIDQLLIPNKNLKKGIIMQETGEGRAEMSVYSLYINKETRT...ESHVTEKGDVKSTIGNLLASAQSQRPAVSCRVDESEKGNVNLL:1244

*Dr* Xinα2 MHSTVLEIPFQQPGSATNPE...AECKTQEAVKIIENLLVQQKEIKTGLVMQESEGGQPEMTVYSLHCES.SLT...ESQTITRGDVKSTIGNLLATVHSQQTKQSCRMEEIERGNVNLY:1248

*Hs* Xinβ VNLTKTQLLNRSTEFHAEKEEIVKGDVQQAIKNLFSEERSV....KKGILIQEDEKGDINMTIYCL.LHENDGD.TIEREEVIGGDVKRTIHNLLSSTSNNKISERAKIDASERGNVQFF:1746

*Pt* Xinβ VNLTKTQLLNRSTEFHAEKEEIVKGDVQQAIKNLFSEERSV....KKGILIQEDEKGDINMTIYCL.LHENDGD.TIEREEVIGGDVRRTIHNLLSSTSNNKISERAKIDASERGNVQFF:1677

*Mam* Xinβ VNLTKTQLLNKSTEFHAEKEEIVKGDVQHAIKNLFSEERSV....KKGILIQEDERGDINMTIYCL.LHENDGD.TIEREEVIGGDVRRTIHNLLSSTSNNKISERAKIDASERGNVQFF:1752

*Cf* Xinβ VNLTKTQLLNRSTEFHAEKEEIVSGDVQQAIKNLFSEDRSV....KKGILIQEDERGDVNMTIYCL.LHENTGD.TIKREEVIGGDVKRTIHNLLSSISNNKISERAKIDASERGNVQFF:1564

*Ec* Xinβ VNLTKTQLLNRSTEFHTEKEEIVSGDVQQAIKSLLSEESSV....KKGILIQEDERGDINMTIYCL.LHENAGD.TIEREEVIGGDVKRTIHNLLSSISNNKISARAKIDASERGNVQFF:1518

*Bt* Xinβ VNLTKTQLLNRSTEFHAEKEEIVSGDVQQAIKNLFSEERSV....KKGILIQEDERGDINMTIYCL.LHENAGD.TIKREEIVGGDVKRTIHNLLSSISNNKISERAKIDASERGNVQFF:1566

*Mm* Xinβ VNFTKTQLLNRSMEFHAEKEEIVRGDVKQAIQKLFSEERRA....KKGILIQEDEKGDINMTIYCL.LHENAGD.KTEREDILGGDVRRTIHNLLSSASNGKISERTKIDASERGNVQFF:1517

*Rn* Xinβ VNFTKTQLLNRSMEFHAEKEEIVRGDVKQAIQKLFSEERCA....KRGILIQEDEKGDVNMTIYCL.LHENAGD.KTKREDILGGDVRRTIHNLLSSASNDKISERTKIDASERGNVQFF:1515

*Md* Xinβ VSLTKAQLLNRSTEIQSEKEEIVRGDIQQAIKNLFSEERAV....KQGIIIQEDERGDINMTIYCL.LHENDNDCKLEHEEIIGGDVKRTIHNFLSSAANYEIAKKTKIDASERGNVQFF:1567

*Ac* Xinβ VNLTKQQLLNRSTDVHIEKEEIVRGDIQQAIKNLFSKDSSV....KHGILIQENERGDINMTVYSL.FHKKDGN.NIKQDEIIGGDVKRTIHSLLSSAMNNEILERPKIDDSERGNVQFF:1430

*Xt* Xinβ VSLTKSQLFNRTQDVEEQREEIIGGDIQDTIKNLLNIQNFS....KQGILIQESEKGDIKMTVYSL.LNQSDQN.TMQRDEVIGGDIKRTISNLKSSGESSENRERVKIEDSERGNVQFY:1505

*Tn* Xinβ2 ..............................................................................KQEIVRGDIKSALQ.KLSGSDKSDQAVKIVVDASERGNVNFY:498

*Tr* Xinβ46 ISSTVKQLFNQERCTSIEKEEIIRGDIQEAVKNLFNENDSG....KHGILIQEDEMGDVQMTIYSL.LNKEENV.NVEKQDIVRGDIKSALQ.KLSGSDKSDQAVKITVDASERGNVNFY:1618

*Ga* Xinβ16 ISSTVHQLFNQESDSSVEKEEIVRGDIREAINKLFDQSGSA....KHGILIQEDEKGDVRMTLYSL.LNKQENV.NVDREDIVRGNIKSALQ.SLSSSDKNDQAMKIKIDETEKGNVNFY:1476

*Ol* Xinβ21 ISSTVEQLFSQERSSNVEKEEIMRGDIQEALHSLFNENSST....KRGILIQEDEKGDVQMTIYSL.LNKHDSM.SVEKEDIVKGDVRSVLE.RLSSPDSLDQAVKIKIDDAEKGNVQFY:1481

*Dr* XinβNA ISGTVQQLLQEHSGTSVEKEEIMRGDIQEAINNLLKEESTG....KRGILIQEDERGDIRMTIYSL.FNSHEES.STEKEYIVRGNVKGCLE.RLCNPDT.EEVARIKVEETERGKVSFY:1386

Tn Xinβ3 VNTTVKQLFNQERGLNIEKEEIIAGDIQEAINSLLKNEGSS....KRGILIQENEKGDVKMTIYSL.LNQGETT.CMEKEDIIHGNVSRTLHRLLSSTAEDE.CKKIRVGDTERGNVSFY:1232

Tr Xinβ36 VNNTVKQLLNQERGINIEKEEIIAGDIQEAISSLLRNEGSS....KHGILIQEDEKGDVKMTVYSL.LNKGETT.CVEKEDIIHGNVSRTLHRLLSSTAEDE.CKKIRVGDMERGNVSFY:1764

Ga Xinβ1 INTTVRQLFNQERGINVEKEEIVRGDIQEAVNNLLKSEGSS....KRGILIQEDEKGDVKLTIYSL.LNKGERA.SMEKEDIVQGNVSRTLHRLLSNSGGED.SKKIRVGDKERGNVSFY:1527

Ol Xinβ2 INTTVKQLLSQERGINVEKEAIIRGDIQEAMTNLLRTEGSS....KRGILIQEDEKGDVKMTIYSL.LNKGEKA.SMEKEDIIQGNVSKTLHRLLSNSGEEE.SKKIRVGEIERGNVSFY:1490

Dr Xinβ6 INSTVQQLFNQDKSGNVEKEEIIRGDIQEAINNLLKQEGSS....KRGILIQEDEKGDVRMTIYSL.LNKETES.SVEKEDIISGNIRGTMHRLLGNTDAKDPSTKITVAEAERGNVSFY:1821

Dr Xinβ19 INSTVQQLFNQDKSGNVEKEEIIRGDIQEAINNLLKQEGSS....KRGILIQEDEKGDVRMTIYSL.LNKETES.SVEKEDIISGNIRGTMHRLLGNTDAKDPSTKITVAEAERGNVSFY:1563

Pm Xin LRQTLERLMHQSSEASTEREEVIRGDIRQAIDQLFSQSDASG..LRRGILIQEGETGNVKMTIYSL.LNREEDS.SRREEDVVAGNVRGAINSLMTSAQSDSRHQQVQQDEIQGGNVQFF:1392

* *** *** *** ***

*Hs* Xinα ASCIDKGDLSGLHS.............LRW...EPPADPSPVPASEG...........AQSLHPTESIIHVPPLDPSMGMGHLRASG.ATPCPP..........................:995

*Pt* Xinα ASCIDKGDLSGLHS.............LRW...EPPADPSPMPASEG...........AQSLPPTESIIHVPPLDPSMGMGHLRASG.ATPCPP..........................:995

*Mam* Xinα ASCIDKGDLSGLHS.............LRW...EPPADPNPVPASKG...........GQSLPPAESIIHVPPLDPSMGMGHLRASGQAIPCPP..........................:996

*Cf* Xinα ASCIDKGDLSGLHS.............LRW...EPPADPSPVPASEG...........APKLPPTENIIHVPPLDPSMGLGHLRGPG.GTSHLP..........................:999

*Ec* Xinα ASCIDKGDLSGLHS.............LRW...EPPADSSPVPTSEG...........AQKLPLTESIIHVPPLDPSMGMGHLRGPG.ATSCPP..........................:999

*Bt* Xinα AGCINKGDLSGLHN.............LRW...EPPADSSPVPASKG...........AQKLPPAESIIHVPPLDPGKGMGHLRGPG.ATPCAP..........................:997

*Mm* Xinα ASCIDKGDLHSLHS.............LRW...EPPTDPSSGPATEE...........SQRVPQTESIIHVTPLDSTMEMGQLRISG.STPCPPP.........................:1000

*Rn* Xinα ASCIDKGELHSLPS.............LRW...EPPTDSSSVPVTEE...........TQRLPPTESIIRVTPLDSTMKMGHLRVSG.STPCPPL.........................:999

*Md* Xinα ASCIEKGDLDSLKS.............LQW...EPPSENGCPAEGAA...........APSQTPPMVSSVNKVIHVALPDGGLDCPRGTVPTPT..........................:995

*Gg* Xin ASCIEKGDLDYLKN.............LQQ...ESEIQSLISAQAEQGAAESAPRALQSTNTHVLANKEQVEKVMAEAKSGALEGAKMVFACESTGKEGALEREVVHAVGVTGTTVQCLG:1379

*Ac* Xinα TSCIEKGDLDYLKN.............LQR...ESEIESLASSQVQE...EPMETVQQAKVTAQLPEGKLVP.QMAGVVLGDISGTKQVFLCKS..KEGTLEREA..E..IG.STEHCL.:1379

*Xt* Xinα TSCIEKGDLGYLRS.............LQD...ESEIEALMLSQSED......A..KASTEQAKKFSEKDGSVQVAEGQRSTIKGKESMCTTSN...................PAIHCMD:1243

*Tn* Xinα15 .....................EVDG.LLG..QEHI.EIVHGDVKEA...........K..RSLHQQKEQVERTVSDVLPGDVKNTKKVFSSESP.........................:1256

*Tr* Xinα296 KTCIEKGDLKSI....HTSSADEVDS.LLA...QEQIEIVHGDVKEA...........K..RSLCQQKEQVERTISDVLPGNVKSTKKVFSSEAS.........................:1315

*Ga* Xinα3 .................................EEYIEIVHGDVKEA...........K..RSLCLQKEQVERTISDVLPGDVKNTKKVFSSEFS.........................:1226

*Ol* Xinα17 KSCIEKGDLEYLRSLYAEASECEADGGLLV...KEQFDTVHGSVKEA...........K..RSLHQQKEHMERSICDVLPGDVKNAKKVFSSECA.........................:1323

*Dr* Xinα2 KSCIEKGDLKSLQR....ELS.EEDLVTSC...RDQIEIVQGDVKEA...........M..RHLSQQREQVERTILDVVPGDVKNVKKVFSDVCTD........................:1323

*Hs* Xinβ TTCIEAGALDYLKQLHTESNE.T....LTAKKQEGEKEIIGGDVEGT...........KLLLKKRQSLVERTVSETDIIPGDVHNTVKVFMTEPQST.......................:1827

*Pt* Xinβ TTCIEAGALDYLRQLHTESNE.T....LTAKKQEGEKEIIGGDVEGT...........KLLLKKRQSLVERTVSETDIIPGDVHNTVKVFMTEPQST.......................:1758

*Mam* Xinβ TTCIEAGALDYLKQLHTESNE.T....LTAKKQEGEKEIIGGDVEGT...........KLLLKKRQSLVERTVSETDIIPGDVHNTVKVFMTEPQST.......................:1833

*Cf* Xinβ TTCIETGALDYLKQLQEGSNE.T....LTARKQ.GEEEIIGGDVEGT...........KLLLKKRQSQVGRTVNEADIIPGDVQNTVKVFMTEPQTT.......................:1644

*Ec* Xinβ TTCIETGALDYLKQLQTGSNE.T....LTTRKQEREEEIIGGDVEGT...........KLLLKKRESQVERTVNETDIIPGDVHNTVKVFMTEPQNT.......................:1599

*Bt* Xinβ TTCIETGALDYLRQLQTGSNE.I....LTGRKQEKEEEIIGGDVEGT...........KLLLKKRQSQVERTVNETDIIPGDVRNTVKVFLTEPQNT.......................:1647

*Mm* Xinβ TTCIETGALDYLKQLQTGSNEST....LTASKQEGEEEIIGGDVEGT...........KFLLKKRQSSFERTVSETDIIPGDVRHTVKVFMTEPQSS.......................:1599

*Rn* Xinβ TTCIETGALDYLKQLQTGSNE.T....LTARKQEGEEEIIGGDVEGT...........KFLLKKRQSSIERTVSETDIIPGDVRNTVKVFMTEPQSA.......................:1596

*Md* Xinβ TTCIEAGALDYLKLLQTGSNE.T....VTTGKQEGEEEIIGGDVEGT...........KLLLQKRKSQIERTVNETDIIPGDVHNTVKVFMTEPLST.......................:1648

*Ac* Xinβ TTCIEAGALDYLKLLQTGTNETF....STRNQEEEEEEIIGGDVEGT...........KLLLKKKKSKIQRTVNEADIVPGDVCNTVKIFMTEPQNT.......................:1512

*Xt* Xinβ TTCIESGALDYLRQLQKSTDESV....EIK....EHEEIIGGDVEGT...........KLQLKRQQFQIERTVDETDIIPGDVYNTVKVFMTEPEHK.......................:1583

*Tn* Xinβ2 STCIESGSLDYLKQLHLGPEEPL....QCI...AEKEKIIGGDVKST...........KLLLSCNQTLIERTV..EDVIPGDVHNTVKVFMSEPTL........................:574

*Tr* Xinβ46 STCIESGSLDYLRQLNLGSEESL....QCT...AEKEKIIGGDIKST...........KLLLGCNRTPIERTV..EDVIPGDVHNTVKVFMLEPTL........................:1694

*Ga* Xinβ16 STCIESGALDYLKQLHVGSDETL....PDR...VEKEKILGGDIKGT...........KLILGRSQTQVDRTV..EDVVPGDVQNQVKVFMSEPTLS.......................:1553

*Ol* Xinβ21 STCIESGALDYLKQLHLEPEETL....LDT...DKKEKIVGGDIKGT...........KLILGRHQTQVERTV..EDVIPGDVHNTVKVFMSEPTTS.......................:1558

*Dr* XinβNA STCIESGALDYLKKLQVEPDEGD....LGK...IEKEEIIGGDIKET...........KLILTRNQAQIGRLVDREDIVPGDVHNTVKVFMTEPQVS.......................:1465

*Tn* Xinβ3 STCIESGALDYLKQLQCEPDEA.....QEK...VEKECIIGGDIEGT...........KILLRKNQQQIGRTVAEDDIVPGDVHNTVKVFITEPAVT.......................:1310

*Tr* Xinβ36 STCIESGALDYLKQLQCEPDES.....QEK...VEKECIIGGDIEGT...........KILLRKNQQEIGRTVAEDDIVPGDVHNTVKVFITEPAVT.......................:1842

*Ga* Xinβ1 STCIESGALDYLKQLQYEPGET.....QEE...SRKENIIGGDIQET...........KILLRKSQQEIGRTVAKHEIVPGDVHSNVKVFMTEPAVT.......................:1605

*Ol* Xinβ2 TTCIESGALDYLKQFQSESSEE.....HEE...VQKEHIIGGNVEET...........KMLLRKNQQQVERTVAEDDIVPVDVNSIVQVFMTEPSVT.......................:1568

*Dr* Xinβ6 STCIESGAMDYLKQLQVGADEI.....VEE...KVKEQIIGGDVEHT...........KQILRKSQQIIGRTVAEDDIVPGDVNNTVQVFMMEPVLS.......................:1899

*Dr* Xinβ19 STCIESGAMDYLKQLQVGADEI.....VEE...KVKEQIIGGDVEHT...........KQILRKSQQIIGRTVAEDDIVPGDVNNTVQVFMMEPVLS.......................:1641

*Pm* Xin TTCIESGALDYLKLLQKQGAEE..........................................DDDESTRRAQEIDIVPGDVQKTKQLLELSAQQA.......................:1447

*** ***

*Hs* Xinα .............QAIGKAVPLAGEA.AAPAQLQNTEKQEDSH...........SGQKGMAVLGKSE.......GATTTPPGPGAP.......DLLAAMQSLRMATAEAQSLHQQVLNKH:1076

*Pt* Xinα .............QAIGKAVPLAGEA.AAPAQLQNTEKQEDSH...........SGQKGMAVLGKSE.......GATTTPPGPGAP.......DLLAAMQSLRMATAEAQSLHQQVLNKH:1076

*Mam* Xinα .............QSIGKAVPLAGEA.AAPAQLQNTEKQEDSH...........SGQKGMAVLGKSE.......GATTTSPGPGAP.......DLLAAMQSLRMATAEAQSLHQQVLNKH:1077

*Cf* Xinα .............QTMEKPVPLAGE...........KKQEDSH...........TGQKGMAAWGKSE.......ETTTTPLGPGAP.......DLQAAMQDLRMATAEAQSLHQQVLNKH:1070

*Ec* Xinα .............RAIGKAVPPAGE........E...KQEDIC...........SGQKGKAALRQS........GATSTAPGPRIP.......DLQASRQSLRMATAEAQSLQQQVLNKH:1069

*Bt* Xinα .............QAAGKAVSLAGE...........EKQESRC...........TGQKGTAALGKSE.......GAMTMPPGPRFP.......ALQVTMQSRRTPTAQAQSLQQQARSKH:1068

*Mm* Xinα S............RAAGKVVLPNGKPVAQAPLQEARKKTDISH...........AGQKGKAASGRPE.......GTIASPLGSGAP.......DLQEAMQNLRLATAEAQSLHQQVLSRH:1083

*Rn* Xinα P............RAAGKVVLPNGRP..GAPLQEARKQVDISH...........AGQKGKAASGRPE.......GTIAPPLGSGAP.......NLQEAMQNLRLATAEAQSLHQQVLSRH:1080

*Md* Xinα ..............GLLTDVPPEPQ..............................GQLKQEGPRESP.......AILGDPQGGPDP.......DLQVAMQNLRLATAEAQSLQHQVQSKH:1057

*Gg* Xin K.PQNLPTGMEKEEIMSGGLKVTTKSIQRVADVSKNTEKEESISACLKEPKATMQGIAQAKVTAERNEVVGEQQSLVTEQASQKQSEEKVLGNDLQAAMQSLRLATAEARNIQHHVQSKL:1498

*Ac* Xinα ..VQSLKPVVVEGNQVPGQVKFPIESVQKAQDDSNRVVREEKIILGERE.....EGSATTKVTEQMKVTSGTTHSSPLGTVDQPQTP..ALGSDLQAAMQSLRLATAEAQSLRHQFQSKL:1490

*Xt* Xinα PGAKGMKCCVAADGTPLKPTEMARNQCILETNVTKNEEKED.............NCARQTTS............TLPPE..GASSR.......DLHAALLDLRQATAEAKTIQKQVECKF:1329

*Tn* Xinα15 ...FSVENCVPKEAIIPGDVLSAKQQL..ALQPPIKVEKEDIVAGDIKAMMESLERAKQQSMHVEREVIKPG..TIYDMDLSTQAPEED....E..SQPQKEVIISGDVKAAKRSLEIAK:1363

*Tr* Xinα296 ...FSVENCVPKEEIIPGDVLSAKQQL..AVQPPLKVEKEDIVAGDIKAMMESLERAKQQSMYVEREIIKPG..TIYDMDLSADAPEGN....E..SQTQKEVIISGDVKAAKRSLEIAK:1422

*Ga* Xinα3 ...LCVENCPPKEEIIPGDVSSAKQQL..AVLPPVVVEKEEIVAGDVKATMQSLERAKQQSMCVEREIVQPG..TIYDMNLSGPDIEG........TQAQKEVIISGDVRAAKKSLEMAK:1331

*Ol* Xinα17 ...LSIESCILKEEIIPGDVSSAKKQL..FVKQPIILDKEEVVPGDIKATMESLELAKEQSRHMEREIIVPG..TIYDIDLSTHGPVEE....G..NQAQKEVIISGNVKAAKRSLEMAK:1430

*Dr* Xinα2 ...LSIGNCVPREEIVRGDILSAKQQLGEAVKQQVMVQKEEIVSGDIKATLESLERAKQQSMQVEREVIKPG..TIYDLNVEAEEMCSE....ENESKLVKEEIIPGDIKAAKRSLERAK:1434

*Hs* Xinβ ......FGKIPKEEIIKGDLTSTLNSLSQAVNQKTVTKTEEIIKGNMLATLKSLKESSHRWKESKQPDAIPG..DIEKAIECLEKAT......NTKTEILKKELLKDDLETSLRSLKEAQ:1933

*Pt* Xinβ ......FGKIPKEEIIKGDLTSTLNSLSQAVNQKTVTKTEEIIKGNMLATLKSLKESSHRWKESKQPDAIPG..DIEKAIECLEKAT......NTKTEILKKELLKDDLETSLRSLKEAQ:1864

*Mam* Xinβ ......LGKIPKEEIIKGDLASTLNSLSQAVNQKTVTKTEEIIKGDMLATLKSLKESTRRWKESKQPDAIPG..DIEKAIECLEKAT......NTRTEILKKELLKDDLETSLRSLKEAQ:1939

*Cf* Xinβ ......FCKTPKEEIIKGDLQSTLNSLSQAINQKTVAKTEETIKGDMLTTLKSLRESSHQWRESKQPNAIPG..DIEKAIECLEKTA......STRTEILKKELIRDDLEISLRNLKEAQ:1750

*Ec* Xinβ ......SCKTPKEEIIKGDLKLTLNSLSQAINQKVVAKTQEIVKGDMLATLKSLKESSHQWKEPKQSDAIPG..DIEKAIECLEKTA......NTRTEILKKELIRDDLEASLKNLKEAE:1705

*Bt* Xinβ ......SCKLPKEEIIKGDVKSTLNSLSQAVSQKTVAKTEEIIKGDMLTTLKSLKESSQKWKDSKQPDVVPG..DIEKAIECLEKTA......RTRTEILKKELILDDLEASLRNLKETQ:1753

*Mm* Xinβ ......SYKTVKDEIIKGDLKSTLNSLNQAMNQKTVAKAEEIVKDDRLAILKSLKESGDRQKEPKQSGGMSR..DIGQAIECLERAT......NTRTEILKKELILDDLKTSLRSLKEEQ:1705

*Rn* Xinβ ......SFKTAKEEIVKGDLKSTLNSLNQAMNQKVVAKTEDIMKDDKAAILKSLKESGGRQKEHKQSASISS..DIGQAIECLEKAT......NTRTEILKKELILDDLKTSLRSLKEEQ:1702

*Md* Xinβ ......SCQVPKEEIIKGDLKATLNSLSEAINQKTVTKREEIMKADMYATMKSLEEARHQWKGTEKTDIIPG..DIKQTIESLEKAV......NTKTEILKKELIRDDLESTLRTLKEDQ:1754

*Ac* Xinβ ......SCHVCKEEIIKGDLKAALNSLSQAINQTTVAEKEEIIKADILAILQSLKEAAYNLKETEKPDVIPG..DIKQAIESLEKAR......NTKNEILRQEVVRSDLESTLRSLKAAQ:1618

*Xt* Xinβ ......SFNVNKEEVVKGNLRETLNSLSQAVNQTVIAQKEEIIKADLPATLKSLSESQYQTRETEKADVIPG..DIQGTINSLEKAA......NIKQELVKEEVVRGNLEATLKSLQEAQ:1689

*Tn* Xinβ2 .......EKPQKEEIIKGDLQAALTSLSKTANHTIVVEKEEVVKGDIPKALRSLERAHKKYKEVEKPEIVPG..NIKGAMRSLERSS......TSKVETIVD.LVPGDVKATLKSLELAK:678

*Tr* Xinβ46 .......ERPHKEEIIKGDLRAALTSLSESANQNVVVEKEEVVKGDIPKALRCLERAHKQYKEVEKPEIVPG..NIKGTMRSLEKSS......TSKAETVVEDLVQGDVKGMLKSLELAK:1799

*Ga* Xinβ16 ......SERLQKEEVVKGNLKAALNLLSESANQTVVVEKEEVVKGNIPKALRSLEKAQKRHKEVEKPDIVRG..NIKGALRSLEKSS......TSRVEAALEDLVPGDVRATLKSLELAK:1659

*Ol* Xinβ21 ......AEKIQKEEIVKGDLRAALNSLSESGKQTVVQ.KEEVVKGNIPKALRCLERAQRNHKEVEKPDIVPG..NVRGAMRSLEKSA......TSRVECLIEGLIPGDVKATLRSLEQAK:1663

*Dr* XinβNA ......FEHLQKEEIVRGDLQAALNSLTQSINQAVVLEKEEVVKADLTMTLRSLEEAQNQPKEVEKPEIIPG..NIKGALQLLKDSS......STKVEVVVEDLVPGDIKGTLKSLEEAK:1571

*Tn* Xinβ3 ......YRNLEKQDIVKGDLCAALDSLTQAINQKVVNRER.................................................................AGGER..........:1349

*Tr* Xinβ36 ......YKNLEKQDIVKGDLCAALDSLTQAINQKVVIEKEQVVKGDIPSTLKCLEEAQHQAKEMEKPEIVKG..DIRGALQSLEKSV......SSSVEATIEDLVAGDVKGTLKSLEEAK:1948

*Ga* Xinβ1 ......YRNLEKRDIVKGDLNAALDSLTQAMNQKVLIEKEEVVKGDIPTTLKSLEEAQHQAKEMEKPEIIRG..DIKGALESLEKSA......TTNTEVTVEDLVPGDIKGTLKSPEEAK:1711

*Ol* Xinβ2 ......YKNVETKDIVKGDLSAAMDSLNQAITQKVVIEKEEVVKGNIPTTLKSLEEAQHQAKEMEKPEIIRG..DIRGALESLEKSA......SVKTEATVEDLVPGDVKGTLRSLQEAK:1674

*Dr* Xinβ6 ......LHNLQKEEIVKGDLRAALDSLTQAVNQCVVIEKEDIVKGDINTTLRSLEEAQNQSKGIEKPEIVPG..DIRGALESLEKST......SAKTEVIIEDLVPGDIKGTLKSLEEAK:2005

*Dr* Xinβ19 ......LHNLQKEEIVKGDLRAALDSLTQAVNQCVVIEKEDIVKGDINTTLRSLEEAQNQSKGIEKPEIVPG..DIRGALESLEKST......SAKTEVIIEDLVPGDIKGTLKSLEEAK:1747

*Pm* Xin .......MVVEKEEVTRGDLQATLRSLEDAVNAKTSVVREDVMRADLRATLQSLQEAVN...................................TKVEVAKEDIVRGDLRATLQSLEEAL:1525

*** *** * ** ***
