# Supplementary material for: Emergence of Xin Demarcates a Key Innovation in Heart Evolution
Source: PLoS One. 2008 Aug 6;3(8):e2857. doi: 10.1371/journal.pone.0002857 (PMC2478706; doi:10.1371/journal.pone.0002857)
Supplement: Figure S5 — Multiple sequence alignment of the C-terminal proline-rich regions of the Xins. The amino acids residues from #1,245 to 1,334 of mXinalpha and from #1,961 to 2,069 of mXinbeta are highly enriched in proline and then defined as the C-terminal proline-rich regions. Although both Xinalphas and Xinbetas contain this C-terminal proline-rich sequence, the two regions are highly divergent. (A) Sequence alignment of the C-terminal proline-rich region of Xinalphas. A high sequence homology was observed among all mammalian Xinalphas. (B) Sequence alignment of the C-terminal proline-rich region of Xinbetas. A high sequence homology was observed among all mammalian Xinbetas. The conserved proline residues are indicated with a star (*) at the bottom of the alignments. (0.09 MB DOC) [file pone.0002857.s005.doc]

**A.** Multiple sequence alignment of the proline-rich region of Xinα proteins

*Hs* Xinα SPHPHNAFVPPPPTLPAAVTG..PDFPAGAHRAEDSIQQASEPLK..............DPLLHSHSSPAGQRTPGGSQTKTPKLDP........TMPPKKKPQLPPKPAHLT.......:1334

*Pt* Xinα ................AAVTG..PDFPAGAHRDEDSIQQASEPLK..............DPLLHSHSSPAGQRTPGGSQTKTPKLDP........TMPPKKKPQLPPKPAHLT.......:1258

*Mam* Xinα SPHPHNAFVPPPPTLPAAVTG..PDFPAGARRDEDSIQQASEPLK..............DPLLHPHSSPAGQKTPGGSQTKTSKLEP........TMPPKKKPQLPPKPAHLT.......:1335

*Cf* Xinα SLHSRNASVPPPPLLPAAVTG..PDFPAQARLDEDSIRQDSK...................LLPSHGSPASQRTPGEAQTRTPILEP........KMPPRKKPPLPPKPAHLS.......:1319

*Ec* Xinα SLHSHNASVPPPPPLPAAVTG..PDFPAHAHHDEDSIRQASEPLQ..............DTLLHSHNSPAGQRTPGGSRTKTSKLEP........TMPPRKKPQLPPKPAHLS.......:1332

*Bt* Xinα SLHSRNASVPPPPPLPASVTG..PDFPAQPRHDENSIRQTSKPTQ..............DPLLHSHNSPAGQKSPGQPQTKTLKPEP........PTHLRKKPQLPPKPAHLS.......:1314

*Mm* Xinα SLHSHNASVPPPPTLPAAVTED.PDHPTQGHHQEDSIQQAPEPLQ..............EPLLHIHNRPSGQKTPEGSETKPSKAES........TMLPRKKPPVPPKPAHLS.......:1334

*Rn* Xinα SLHSHNAS...PPTLPAAVTEE.PDHPAQGHHQKDSVQQASKPLR..............EPLLHSHNRPAGQKTLEGSETKLSKAES........TVLPRKKPPVPPKPAHLS.......:1328

*Md* Xinα GPQALNAS..PPP..PAAVTG..PELPPLAGPEDNTLPRSAAP.Q..............KPPARLLGPASGQRPPGRPEAELPRQETS.......AMAARKKPALPPKPAHLGGP.....:1304

*Gg* Xin SGSQADNDFPPPPPVAVMKAEHCPPSTKATREGAPPLLTSKDEAPGCFSPLQTPLPPPPSLSCKPSDQNSTEKPKIPPKPEITAPLRK......KPVPPPKPEHLLHEAYSASTN.NSTN:1828

*Ac* Xinα SGSQAINDCPPPSPASVMGTECQPSKQKE....AKALRCSSLAQP......HVPKAPPPAP.TKPSDQKALEKPQLPPKPEVIAPPRR......KPVPPPKPEFLLKEALPHPA...SCK:1797

*Xt* Xinα LVPPSDNDFPPPPAAVIEQEHCLPSKIEAK...RDDTMEVQEPAP..........IFMDSAANKASDTILDENANTLATPQPLMTQRK......KPVPPPKPKHLLPGACPPPHL.RAKC:1638

*Tn* Xinα15 SMPPSDTKLSPSISVTCEGQPP...ITTQNSPSNAVTNGNARSST...SEAQTAPPPTPPTPRPQKTREKLQKPALAPKPQWTNSVVVH....QQNISLPSAPEVSQTNDP.......PR:1646

*Tr* Xinα296 SMPPSDTKLSPSISVTCEGQPP...ITTQNSPSNAVTNGNAKSST...SETQTAPPPPPPFS..QKTCEKLQKPALAPKPQWIKSVKE.....QPNILLPSAPEVAHPSQAN.....TKQ:1704

*Ga* Xinα3 SMPPSDTVLSPSISVTCEGQPS...VTTQNSTPDPVANGISKSS....SPASVTPPPLPQKK...IQKPQELKPALPPKPQWTKSVVVE....EPNISAAPEVTCHTKDNMKSATIPSKQ:1597

*Ol* Xinα17 SVPPSDTTLSPSISVTCEGQPA...IATQSPSPNPIAHGNTSVS....WSEGVTPSLPPEKG....EKPQEQKPALPPKPQWMKSVAP.....ERPTPAPSAASPQTQKQP.........:1705

*Dr* Xinα2 HIPSSHTQLSSSVSVTRSEHPTSLALNSESVSSNADNSKNSSAFT...GKDEHPPPILPKTG....HQVKDQKPVIPPKPLHITTSSPPLFTETSNMCPNSTVSINDTQQTPAIPLKVTP:1727

  * **** * * * * ****** * * ** * * * ** * ** *

*Hs* Xinα ...QSHPPQRLPKPLPLSPSFSSEVGQREHQRGERDTA....IPQPAKVPTTVDQGHIPLARCPSGHSQPS.........LQHGLSTTAPRPTKNQATGSNAQSSEPPKLNALNHDP:1435

*Pt* Xinα ...QSHPPQRLPKPLPLSPSFSSEVGQREHQRGERDTA....IPQPANVPTTVDQGHIPLARCPSGHSQPS.........LQHGLSTTAPRPTKNQATGSNAQSSEPPKLNALNCDP:1359

*Mam* Xinα ...QSHPPQRLPKPLPVSPSFSSEVGQREHQRGETDAA....IPQPVKVPTTVGQGHIPLARCPSGHSQPS.........PQHGFSTTAPRPTNNQATGSNAQSSEPPKLNALNRDP:1436

*Cf* Xinα ...QICPPQRLPTPSALSPSSCKESGQGEHKPGERDAT....TLRPANIPTTAGQGCVSLSGGPSEQNQPS.........PQSGPSTMASRPTESQAARCHDQSPKSPKFSALSSHP:1420

*Ec* Xinα ...QIRPPQRPPKPLALSPGSSKEVGQGEHKQGERDAA....ILPSAKVPTTAGQGRVPLAGCPGGQSQPG.........SQHGHSTMATKPTRGQAAGSNTQSPEPPKVSALSSDP:1433

*Bt* Xinα ...QLPLPRWLSKPAALAHSAAEEGGQGKHKQGETATAN..HDPRPHRVSIAADQGRVSLPQGPAGQSQPS.........PQHGPSTVAPSPTKSQAIGSNNHSPDPLRLSALSSHP:1417

*Mm* Xinα ...QIHPPQRLPKPLAGSARAS.EAGQ.DHKPGEPGIAN....PGSDKAPTIAGQDCLPLAESSKGQKQPA.........HQRPLSSMASRPSRGQITSSNSQSPESPKLNVLNNDS:1433

*Rn* Xinα ...QVHPPHRLPKPLAASASIS.EAGQ.DHKPGEPGVAN....PGSAKAPTIAGQGCLPLAECSKGQKQPM.........RQYPLSTTASRPSGGQTSNSNSQSPESRKLTILNNDN:1427

*Md* Xinα ..APILPPQLPPKHPALPSSPVGTGAP..GEVQAPTSG...........HLDG....RPLPGRPSSTHKSSE........RQAVVSGVQAVSPRRPPCPSTTLQDDPAALQGPRGLP:1394

*Gg* Xin RSTKSVPPPVPPKPPGLREISMPKP.....PPAELQLSCTEVCEQSDHRESQDKCCTLESSMDKSITVHGPE........RKLPKYTAKTPLQMAEERYKARK..GGQGKFELD...:1927

*Ac* Xinα KGTLGIETTQLSSPKPLTEVEQSKSKTLPIPDCAKAAFGTEIWEPSPCQQHSTVAPVAPSSVSTVVEDFRSE........KKPRRDMVKTPLQVAEEKYKAKK..EEQSKVELGDQK:1904

*Xt* Xinα QTKSTSSQNSRSTNDCSSKITISKMTN.EQWQQQFPSESAYASANRKEQESEMFACTANEQLKPELCLNNNC........ESSEISTSKNDHGQANDFHTQIQ..PLENLVTDT...:1741

*Tn* Xinα15 NRNLPS....AGVQPSGHGETHDEFFDQTHQQNWADETVQDANRKSECVSALLKFKKMNEEETAPPTLPKSDYTAILNSNETGMEKNVIGKISAAEEMRMCMKNYANAGQPETNTRL:1759

*Tr* Xinα296 SKHFPAPF..AGKTQSGDG.TNVEFFHQTHQQNWNDKMVQYSNGKSECVSALLTSQKINIEETAPSTLSKSD....VNVNEREMEKNVIEKINAAEEIKTCMKHYANAGQHEMNTSL:1814

*Ga* Xinα3 SKPIPMPFPDKLERAMSHGKIYKEAGQKTSQQNCVEAS.QDSNQRNERVSTDSKAQETDTGNKSQCRVMMSN....S...SVEMNRNVIQKINAADEIQMCMRNYAGDGKYEMNMSL:1706

*Ol* Xinα17 ..VPTSP.........QSKQ..QDIPTHRTMNKYADEV..DINQTIDSKIEETQMLSITKR.KADFQLPEND.........DGLIK....KINAAEEIQMWKRNHAGEGKEEMNTSF:1793

*Dr* Xinα2 SNKMFTHETEIAKTSNKIKDKESKIHEQVQRTNLTDPTDFQRMQYTEQWVQNSHMQITDTPSVNKTDSFKNGS...FPGDSIGMEKNVVQRINAAEEIRMCYS....KDNDELNKGF:1837

  ** * * * * * * * * * * * * ** *

**B.** Multiple sequence alignment of the proline-rich region of Xinβ proteins

*Hs* Xinβ DTSNVTEMKVSEKSHNTFKATNKKRE.....TDVHLKSQDFLMKTN......TSTGLKMAMERSLNPINFNPENNVKESECPLPPPSPPPPPPSNASSEIEFPLPPPPPLMMFPEKNGFL:2326

*Pt* Xinβ DTSNVTEMKVSEKSHNTFKATNKKQE.....TDVHLKNQDFLMKTN......TSTGLKMAMERSLNPINFNPENNVKESECPLPPPSPPPPPPSNASSEIEFPLPPPPPLTMFPEKNGFL:2231

*Mam* Xinβ DTTSVTEVKVSEKNHNTFKTTNKKQE.....TDVHLKSQDFLMKTN......TSTDLKTAMERSLNPINFNPENNVKESECPLPPPSPPPPPPSNASSEIEFPLPPPPPLMMFPEKNGFL:2316

*Cf* Xinβ DIANITEVKVSPKNHSKFKATDKKQ......ADVHLRSQDFLMKTN......TSADLKMAMEMSLNSTNCNPENNVEESECSLPPPSPPPPPPSNASSEIEFPLPPPPPLMMLPEKNGFP:2137

*Ec* Xinβ DISNVTEAKVSQKSHNKFKATDKKQ......TDVHLKSQDFLMKTN......TSTDLKKAMEMSFNPINFNPENNVKESEWPLPLPSPPPLPPSNASSEIEFPLPPPPPLMMLPEKNGFP:2078

*Bt* Xinβ DMSNVTEVKVSQKSHNQLKAIDKKQ......TDIHLKSQDFLMKTN......TSKDLKMAMEMSFNPIKFNPENNAKENEFPLPPPSPPPPLPSNASSEIEFPLPPPPPLMMLPEKNVFP:2110

*Mm* Xinβ EVHSVPGVTVSGKNHKRIQATDKRQK.....TDVCLESQDFLMKTN......TSKELKMAMERSFNPINLHPECGIKENEDSLPPPSPPPLPPSNASSEIEFPLPPPPPLMLLPGKNESP:2069

*Rn* Xinβ DAHSAPGVTVSGKNHKRTQAPDKKQR.....IDVCLESQDFLMKTN......TSKELKMAMERSFNPVNLYPDCGVKENEDALPPPSPPPPPPSNASSEIEFPLPPPPPIMLLPEKNEFP:2088

*Md* Xinβ DIQSVSEEKATKGNRGKFKGTTERNTM....IDVHQKNENFQTRVD......ASTNLKMTAGKSYNPGKLATVSNVTETHSFLPPPSPPPPPPSNASSEIEFPLPPPPPLMMFSEKHEFS:2158

*Ac* Xinβ NIKTNQKVRTTQEMHTQITESERKQRC....NVIQPSEKSMLRFRGKATVEGAESDVKVMVKNPLNPNKNPKGPDRSEIDSPPPPPPPPSPPISVTSSDADFPLPPPPPPLTHLITASDS:2028

*Xt* Xinβ NIENTEKIQISEKNIQMPKEIKNLEVS....NVIK..KKSMEEVTVKRKFGSSKSGHHLQMKNHPQPAILRNPKEKPEIYSPPPPPPPPPPPVTET.LTAQLPLPPPPPPLPLMKSNNEP:2079

*Tn* Xinβ2 DLPPPPPP..PPSVDSDIEQLPPLP.....PPPPLASEQDFLPPPP......SQQELENMPKQATAPLPVGVKKMTVKNVKGPALHLVPKLEPKVST..TQHVEVELEKVVETTRSQSKA:1029

*Tr* Xinβ46 DLPP......PPSVDSDIEQLPPPP.....PPPPLAGEQDFLPPPP......SQQELESMPKQATPPSPVIAKKMTVKKVKGPALHPVPKLEPKVELSKTRHVEHKSEKVEEISWSQSKA:2148

*Ga* Xinβ16 DLPP......PPSVDSDIDCLPP.......PPPPLNSEQDFLPPPP......SQQELERMPTQAIHRSPAKAKKMTVKKVKAPVLLPVPKLEPKVEFSKTEQVELTSKKIIEISQNQSKA:2004

*Ol* Xinβ21 DILPPPPSPLPPSADTDIDHLPP.......PPPPLTPEQDVLPPPP......SQQELESMPTADIQPFPAKAKRMTVKKVKAPTLHPVPKLEPKVEVIKSQQVQVTSEKRKEMSQKTQES:2018

*Dr* XinβNA DLPLPPTPPPGDPAKTELDHLPPPPSPSPLPPAPPTAELDLLPPPP......TEQELDILPLPSLTPS..KPTKMTVKPVKAPMLCKVPKLEPAISF.EQINVHSVEESRKNVTTSSAKS:1951

*Tr* Xinβ36 NTSQMNISNKQICETKTVKQQELNAMP...RLTPTKPASRPLFKVP......QPPETQKQPVHCTPKW.....QRKQPTPALSQLRSDQASTTSTVCKKETKVEEKKESVQQIETVNQTE:2284

*Ga* Xinβ1 NIRTSTHTNIS...SKQVCETKQTAAE...QTSLVQAENRALLQKH......NAKNLNSGHRSLATRG.....DGGTKKVKPEIHFPPPPSSPPPP.SESELSLPPPPSP..EGLARPPS:2056

*Ol* Xinβ2 SASQWSNSNVQMSQMTTIKQVQTAVTE...ETVTQKQNHTSQKKSG......ALIEKQNPKSDRKNIN.....KGAIKNVKAEIHFPPPPPTSPPPLSESDLSLPPPPSPVMESPLPPPS:2020

*Dr* Xinβ6 EASQTNISMKKTEETKTVTQVQTTMTT...ESTTISQKQN.IKNLK......SEKNVKSLNRNLSPKG.....MIKKTKPQPEIHFPPPPSSPPPP.SESELSLPPPPSPVAESEVQPPL:2358

*Dr* Xinβ19 EASQTNISMKKTEETKTVTQVQTTMTT...ESTTISQKQN.IKNLK......SEKNVKSLNRNLSPKG.....MIKKTKPQPEIHFPPPPSSPPPP.SESELSLPPPPSPVAESEVQPPL:2100

  * * * * * *** ****** * ***** * *

*Hs* Xinβ PSLSTEKIKAEFESFPGLPLP..........PPPVDEKSERESSSMFLPPPPPP.T.PSQKPAHLLSSSAPEKHSGDFMQQYSQKEASNS..QNSQAKIITGKTG.VLPPPTLPKPKLPK:2431

*Pt* Xinβ PSLSTEKIKAEFESFPGLPLP..........PPPVDEKSERESSSMFLPPPPPP.T.PSQKPAHLLSSSAPEKHSGDFMQQYSQKEASNS..QNSQAKIITGKRG.VLPPPTLPKPKLPK:2336

*Mam* Xinβ PSLSTEKIKAEFESFPGLPLP..........PPSVDEKSERESPSMFLPPPPPP.T.PSQNPAHLLSSSAPEKHSGAFMQQYSQKEASNS..QNSQAKIITGKSG.VLPPPTLPKPKLPK:2421

*Cf* Xinβ PSLSPEMIKAEFESFPGLPLP..........PPPEDEKSERDCLSAFPTPLPPP.T.PSRKPAHLLSSSVQGKHTGTFIQ.HSQEEAFSSQQTHSQAKVLKGKSGVLLPPPTLPKPKFPK:2244

*Ec* Xinβ PSLSTETRKAEFESFPGFPLP..........PPPEDEKSERECLSMFPPPPPPP.T.PLPKPAPCLSSSVPEKHSGAFMQ.YSQEEASRSQQTHSQAKIITGKSGVVLPPPTLPKPKFPK:2185

*Bt* Xinβ PSLSTEKIQAEFENFPGLPLP..........PPPEDEKFERECLSTFPPPPPPPPA.PALKPAHLLSSSVQEKHNGTFIQ.YSQEEAS....S.SQAKITTGKSGGRLPPPTLPKPKFPK:2213

*Mm* Xinβ PSSPTEKTRTEFESLSTLPLP..........PPPVDEKAEQECLSTTLPPPPPP...TPCQPGHLLPSSVLGHHREAFLQQFSQKEALGVHLPHSQAKILTGKS....PPPTLPKPKLPK:2172

*Rn* Xinβ PSSPTEKSRAELESLPTLPLP..........PPPGDEKSDQECLPTSLPPPPPT...APSQPAHLLSSSVLEHHSEAFLQQYSRKETLDSHQLHSQAKILTGKS....PPPTLPKPKLPE:2191

*Md* Xinβ SLPSTEKIKGEFDGFPCLPPP..........PPLVDDRSEGECPSTFLPPPPPL...S.QNPINFSSSTIQTKGSGENVQQLCQESVQ....THSRSRSLAGKSTAIPPSPKFPKPKFFR:2260

*Ac* Xinβ QSFPSPPPPVGQGKMESEHFP..........PPPFPVEVKGESELAYAPSPLPLPQ.PSAIPQPSQKKDHFLKHLDKTLQTSIQHHDSLKGSRGSYIKSHLQMES.......AK..DLET:2128

*Xt* Xinβ EYFPSPPPPVT.DKMDNEIFP..........PVTTTLLTEHKEIVNKKPYHKQKTQ.VFSNNYSTNQLESRQKSANEQTIKYSEKQRPDVPPK.SKLPVFQHKKI.......SP..TFKS:2177

*Tn* Xinβ2 T.....DTSKK.FSVEFPAPP..........KSPNPVNKVHMSPVRFTPPPSPPPP..IRGPISKFSTPLIKAEEKYRKLKE............TNTPPTTPVP................:1103

*Tr* Xinβ46 S.....DTSRT.FSLEFPAPP..........QSPKPVNKVHVTPVRFTPPPSPPPP..MRGPISKFSTPLIKAEEKYRKLKE............ASTPPTTPVP................:2222

*Ga* Xinβ16 TT....HTTKT.VSCETPPLP..........ESPKPPKK..VSPVKFTPPPSPPPS..VRGKMTKFNTPLIKAEGKYRKLVE............DNAPPTTPTA................:2077

*Ol* Xinβ21 K.........T.TSPEVTFLP..........ESPRPLKKVYITPVKFTPPPSPPPF..MRGKMTKFNTPLIKAEEKYRKLKE............ENTPPNTPPP................:2088

*Dr* XinβNA TM....VSSKI.QSAFTKQPP..........ESPRPPKK.VFTNLMLTPPPSPPPV..CKSPVSKFKTPLIQAEQKYRQQRE............ESCTPPPPPSRSPAF...........:2030

*Tr* Xinβ36 S......EVVT.KNTPVAKPV........QGKSPKPPKKVFVPPIKLPPTPEPVPASKPRPYACKFKTPLMLAEERYRQQKMEKEKTET....STVTTPISPPINILPC...........:2374

*Ga* Xinβ1 V......MRQD.SDLKGNPEA........EFFPLPQPPAPQPSVSGHDLTTPTLRSWKLTKPSNVFGHEILEESLLLRPCTMPHTINKRKKNLSTHYITIDPELATACYGLRIDVYYSLH:2161

*Ol* Xinβ2 I......TRQD.SDLPPPPPP........PMEFFPPPPPDFLPP...PPSPQELDALPQPPPARPFGKPLFKVHKQPEPPKQPAPVKPK....WQKKAPPPPSSQVFPL...........:2107

*Dr* Xinβ6 LPRPPLAMRQD.SDLPPPPPPPPIMETDTEFFPPPPPPQDFLPP...PPSQQELSS..VAQPAKPKGRALFKVP.TPEPPKQPMPKTFKWQKKQTSPTPPPPPPPSVAE...........:2460

*Dr* Xinβ19 LPRPPLAMRQD.SDLPPPPPPPPIMETDTEFFPPPPPPQDFLPP...PPSQQELSS..VAQPAKPKGRALFKVP.TPEPPKQPMPKTFKWQKKQTSPTPPPPPPPSVAE...........:2202

  * * * * *** * * ******** * * * * *** * * *
