# Supplementary material for: Emergence of Xin Demarcates a Key Innovation in Heart Evolution
Source: PLoS One. 2008 Aug 6;3(8):e2857. doi: 10.1371/journal.pone.0002857 (PMC2478706; doi:10.1371/journal.pone.0002857)
Supplement: Figure S6 — Multiple sequence alignment of the filamin c-binding region of Xinalpha. The filamin c-binding region was previously mapped to the last 158 amino acid residues (aa#1,685–1,843) of one of spliced variant from hXinalpha gene [10], which is the largest but minor Xinalpha in the human heart, equivalent to mXinalpha-a isoform in the mouse heart. Although the major isoform from hXinalpha and mXinalpha did not contain this filamin c-binding region, we have recently shown by yeast 2-hybrid assay that mXinalpha is able to interact with a more ubiquitous isoform of filamin, filamin b. An alignment of all Xins putative filamin c-binding region did not result in overall conservation (alignment not shown). However, the strongest identity to each other was observed throughout the entire filamin c-binding region of all mammal Xinalphas. Thus, the filamin c-binding region appears to be a derived trait in the mammal Xinalpha. (0.05 MB DOC) [file pone.0002857.s006.doc]

*Hs* Xinα TPSFKGNPDVSVKSTQLAQDIGQ..AL.............................................LHQKGVQDKTGKKDITQCSVQPEPAPPSASPLP:1742

*Pt* Xinα TPSFKGNPDVSVKSTQLAQDIGQ..AL.............................................LHQKGVQDKTGKKDVTQCSVQPEPAPPSASPLP:1666

*Mam* Xinα TSSFKGNPDISVKSTQAAQDIGQ..AL.............................................LQQKGVQDKAGKNDITQCSVQPEPAPPSASPLP:1743

*Cf* Xinα APSPRGIFEVSVKSTHLAQAG...................................................LHQKGLQDNAGKKEVTQCPWQPEPAPALASPLP:1722

*Ec* Xinα APSLRGSPGVSVKSTHLAQDVGH..AL.............................................LHQKGVQDKAEKKEATQCSGQPETAPASASPLP:1742

*Bt* Xinα EASPRGNPEISVKRAHFTQDECQ..TQ.............................................PHQKDIWHKAGEKEAPQLSGPPPPGPAAASALP:1723

*Mm* Xinα ASSPQGSHYISGKNTHLGQDIGQ..AL.............................................LYQRDIQDQAGTKEMCIEG...........AVL:1719

*Rn* Xinα ASSPQGSHSISGKNTHLAQDISQ..AL.............................................LYQRDIQDQAGTKEMAIKG...........SVP:1728

*Md* Xinα SPSPGDSPDSPRLSRDGAHPLPQRHPE.............................................LADEPPDGAAADRGLREARQGRPEPSPVASPLF:1701

*Gg* Xin .........................................................................................................:1941

*Ac* Xinα SPQMSRSQYTRGKEVSAQDIFSSLPRKSVRSDDCNLAFSEGEYAPVQMQKGTGLIKQQHLSSPEVLHRHVGTNGDKEQDASQHLIQGAGPEHASKASLSALSPSN:2414

*Xt* Xinα NTSCASSPPKPKALNNGQTPEDSLSKRP...............................................YSPQE................ATSQNSPLN:2081

*Tn* Xinα15 QTKAPMSTFKP.................................................................................TAEDGTQGCRGPA:2084

*Tr* Xinα296 QTKPPMSTFKP.................................................................................TADDCPPSCNSPA:2153

*Ga* Xinα3 QPKLTMSTFKPRTESHSKNGHDANVDLR.............................................................PKAAANEASKQGQPPA:2071

*Ol* Xinα17 QPKPTMMTFKP.................................................................................QADGANQSLDRPQ:2121

*Dr* Xinα2 SPTDKPKTNADQSNAGSS....S....................................................................SQNSSASHICSPPS:2187

*Hs* Xinα RGWQKSVLELQTGPGSSQHYGAMRTVTEQYEEVDQFGNTVLMS..STTVTEQAEPPR.......NPGSHLGLHASPLLRQFLHSPAGFSSDLTEAETVQVSCSYSQPAAQ..:1843

*Pt* Xinα RGRQKSVLELQTGPGSSQHYGAMRTVTEQYEEVDQFGNTVLMS..STTVTEQAEPPR.......NPGSHLGLRASPLLRQFLHSPAGFSSDLTEAETVQVSCSYSQPAAQ..:1767

*Mam* Xinα RGRQKSVLELQTGPGSSQHYGAMRTVTEQYEEVDQFGNTVLMS..STTVTEQAEPPR.......SPGSHLGLHASPLLRQFLHSPAGFSSDLTEAEMVQVSCSYSQPATQ..:1844

*Cf* Xinα TGQQKNVLELQTGLDGSRCYEATGTVTQQCDGVDQCRSPVLLS..STTVTEQAEMTK.......GPGPHLELHASPLLRQFLRSPARLSGALAEAETVHAPCGYSQPGAQ..:1823

*Ec* Xinα TGQQKSLLELQTGPGGSHHYGAMRSMTEQCEGVDHCRNTVLSS..STSVTEQAEPPR.......GPGPHLGLHACPLLRQFLRSPAGLSGGLAEAEMAHVPCSHSQPAAQ..:1843

*Bt* Xinα T.RQKSALEPQTAPGGSRHDGATGAGTERVG...QCRTTALVS..PTTVTEPAEPPR.......GPGPHLGHHTSPLMRQFLHSQTGLSTGLAEAEMLRVPCGHPTPTAQ..:1820

*Mm* Xinα TGQPKNVLEFQTGSTTSKSYGAMRTVTEQYEEMDQFGNTVLTS..STTITQHADPLT.......DPRPQLCLHTSPMLRQLLHSPSRLNSDLAEAEITWTPCNNFHPAAQ..:1813

*Rn* Xinα TGQQENVLEFQTGSATSKSYGATKTVTEQYEEMDQFGNTVLTS..STTITQQAEPLM.......DPRPQLGLYTFPMLRQFLHSPSRFNSDLAGAEIPWVPCSHFHPAAQ..:1822

*Md* Xinα PRRQKSVLELQTGPRGSQLYGTTRTVTEQYEEVDPFGNKIITS..STTVTKQADGPA.......GRGRAYEVSTSPLLRRYLQNSSRANGSLQDAGVVCVTFGNSRAATK..:1802

*Gg* Xin ................................................................................................................:1941

*Ac* Xinα PRRQKSILELQTNHDGSKLFGATRTVTEQYEEVDEFGNKIITS..STTVTKQSETQTSSTCDMVSCPTRYEVTASPVLRRYLNSPADFPSNEGHQEAGVVFVTFSNSKPAKK:2524

*Xt* Xinα PQRQKSILELKQGQRVQNSLAPP.........................................................................................:2103

*Tn* Xinα15 QRN.LSVLEVQSGPEQPDGIVGTKTVSETYQETDGFGNVFLSSVTSTVFTKHSDTQSSPLFDVDASPSTYGVGTSPLIRRSGRPFQDKVASAASAEG.TVFVSFGQPKEKR.:2193

*Tr* Xinα296 QRN.VSVLEVKTAPEEPEGIIGTKTVRETYEETDGFGNVFLSSVTSTVFTKHSDTKSSALFDIDGNPTRYAATASPLIKRSGRPFEDEVANTSNDKG.MVFVSFSQPKEKR.:2262

*Ga* Xinα3 HRN.VSVIEVKTAPEPPAGIVGTKTVSETYEETDGFGNVFLSSVTSTTVTKQSDSKSSALFEVVGNPARYEVMTSPLIRRSGR.TREKVLNHTNEGG.TVFVTFSQPKEKH.:2179

*Ol* Xinα17 QQ..VSVLEVKTLPEKPAGIIDTKTVRETYEESDGFGNVFVSSVTSTFVSNHSDSKASALFEAMGSPTRYEAVTSPLMRRPRHPFDDKVFSRPKEEG.TVFVTFSRPNTDQT:2230

*Dr* Xinα2 PRRKVSVLEVQRVPEVPSGIFGTKTVSEKYEETDCFGNTYYSSKRSTFVTRQSETELSSSYDVVTSPRRSEGMTSPVLQRSGQSFSSNSLSKGKDR..KVFVTFGHPNTEKH:2297
